# Acute-on-chronic liver failure (ACLF): the ‘Kyoto Consensus’—steps from Asia

**DOI:** 10.1007/s12072-024-10773-4

**Published:** 2025-02-17

**Authors:** Ashok Choudhury, Anand V. Kulkarni, Vinod Arora, A. S. Soin, Abdul Kadir Dokmeci, Abhijeet Chowdhury, Abraham Koshy, Ajay Duseja, Ajay Kumar, Ajay Kumar Mishra, Ajay Kumar Patwa, Ajit Sood, Akash Roy, Akash Shukla, Albert Chan, Aleksander Krag, Amar Mukund, Ameet Mandot, Amit Goel, Amna Subhan Butt, Amrish Sahney, Ananta Shrestha, Andrés Cárdenas, Angelo Di Giorgio, Anil Arora, Anil Chandra Anand, Anil Dhawan, Ankur Jindal, Anoop Saraya, Anshu Srivastava, Anupam Kumar, Apichat Kaewdech, Apurva Pande, Archana Rastogi, Arun Valsan, Ashish Goel, Ashish Kumar, Ashwani K. Singal, Atsushi Tanaka, Audrey Coilly, Ayaskanta Singh, Babu Lal Meena, Barath Jagadisan, Barjesh Chander Sharma, Bikrant Bihari Lal, C. E. Eapen, Cesar Yaghi, Chandan Kumar Kedarisetty, Chang Wook Kim, Charles Panackel, Chen Yu, Chetan R. Kalal, Chhagan Bihari, Chien Hao Huang, Chitranshu Vasishtha, Christian Jansen, Christian Strassburg, Chun Yen Lin, Constantine J. Karvellas, Cosmas Rinaldi Adithya Lesmana, Cyriac Abby Philips, Debbie Shawcross, Dharmesh Kapoor, Dhiraj Agrawal, Diana Alcantara Payawal, Dibya Lochan Praharaj, Dinesh Jothimani, Do Seon Song, Dong Joon Kim, Dong-Sik Kim, Duan Zhongping, Fazal Karim, Francois Durand, Gamal E. Shiha, Gennaro D’Amico, George K. Lau, Girish Kumar Pati, Graciela Elia Castro Narro, Guan-Huei Lee, Gupse Adali, Guru Prasad Dhakal, Gyongyi Szabo, H. C. Lin, Hai Li, Hari Kumar Nair, Harshad Devarbhavi, Harshvardhan Tevethia, Hasmik Ghazinian, Hemamala Ilango, Hong Ling Yu, Irsan Hasan, J. Fernandez, Jacob George, Jaideep Behari, James Fung, Jasmohan Bajaj, Jaya Benjamin, Jennifer C. Lai, Jidong Jia, Jin Hua Hu, Jin Jun Chen, Jin Lin Hou, Jin Mo Yang, Johannes Chang, Jonel Trebicka, Jörg C. Kalf, Jose D. Sollano, Joy Varghese, Juan Pablo Arab, Jun Li, K. Rajender Reddy, Kaiser Raja, Kalpana Panda, Kamal Kajal, Karan Kumar, Kaushal Madan, Kemal Fariz Kalista, Kessarin Thanapirom, Khin Maung Win, Ki Tae Suk, Krishnadas Devadas, Laurentius A. Lesmana, Lubna Kamani, Madhumita Premkumar, Madunil A. Niriella, Mamun Al Mahtab, Man Fung Yuen, Manal HEl Sayed, Manasa Alla, Manav Wadhawan, Manoj Kumar Sharma, Manoj Sahu, Manya Prasad, Mark Dhinesh Muthiah, Martin Schulz, Meenu Bajpai, Mettu Srinivas Reddy, Michael Praktiknjo, Ming Lung Yu, Mithra Prasad, Mithun Sharma, Mohamed Elbasiony, Mohammed Eslam, Mohd. Golam Azam, Mohd. Rela, Moreshwar S. Desai, Mukul Vij, Nadim Mahmud, Narendra Singh Choudhary, Navin Kumar Marannan, Necati Ormeci, Neeraj Saraf, Nipun Verma, Nobuaki Nakayama, Norifumi Kawada, Oidov Baatarkhuu, Omesh Goyal, Osamu Yokosuka, P. N. Rao, Paolo Angeli, Pathik Parikh, Patrick S. Kamath, Paul J. Thuluvath, Philipp Lingohr, Piyush Ranjan, Prashant Bhangui, Pravin Rathi, Puja Sakhuja, Puneet Puri, Qin Ning, R. K. Dhiman, Rahul Kumar, Rajan Vijayaraghavan, Rajeev Khanna, Rakhi Maiwall, Ravi Mohanka, Richard Moreau, Rino Alvani Gani, Rohit Loomba, Rohit Mehtani, Ruveena Bhavani Rajaram, S. S. Hamid, Sachin Palnitkar, Sadhna Lal, Sagnik Biswas, Sakkarin Chirapongsathorn, Samagra Agarwal, Sanjeev Sachdeva, Sanjiv Saigal, Santhosh E. Kumar, Sargsyan Violeta, Satender Pal Singh, Satoshi Mochida, Saurabh Mukewar, Seema Alam, Seng Gee Lim, Shahinul Alam, Shalimar, Shantan Venishetty, Shikha S. Sundaram, Shiran Shetty, Shobna Bhatia, Shweta A. Singh, Shyam Kottilil, Simone Strasser, S. M. Shasthry, Soe Thiha Maung, Soek Siam Tan, Sombat Treeprasertsuk, Sonal Asthana, Steffen Manekeller, Subhash Gupta, Subrat Kumar Acharya, Sudhamshu K.C., Sudhir Maharshi, Sumeet Asrani, Sunil Dadhich, Sunil Taneja, Suprabhat Giri, Surender Singh, Tao Chen, Tarana Gupta, Tatsuo Kanda, Tawesak Tanwandee, Teerha Piratvishuth, Ulrich Spengler, V. G. Mohan Prasad, Vandana Midha, Venera Rakhmetova, Vicente Arroyo, Vikrant Sood, Vinay Kumar BR, Vincent Wai-Sun Wong, Viniyendra Pamecha, Virendra Singh, Vishwa Mohan Dayal, Vivek A. Saraswat, WRay Kim, Wasim Jafri, Wenyi Gu, Wong Yu Jun, Xiaolong Qi, Yogesh K. Chawla, Yoon Jun Kim, Yu Shi, Zaigham Abbas, Guresh Kumar, Shuichiro Shiina, Lai Wei, Masao Omata, Shiv Kumar Sarin, APASL-ACLF Research Consortium (AARC) for APASL-ACLF working party

**Affiliations:** 1 Department of Hepatology, Institute of Liver and Biliary Sciences, New Delhi, 110070 India; 2 Asian Institute of Gastroenterology Hospitals, Hyderabad, India; 3 Medanta-The Medicity Hospital, Gurugram, Haryana India; 4 Ankara University School of Medicine, Ankara, Turkey; 5 Institute of Post-Graduate Medical Education and Research (IPGMER), Kolkata, West Bengal India; 6 VPS Lakeshore Hospital and Research Center Ltd, Kochi, Kerala India; 7 Post Graduate Institute of Medical Education and Research (PGIMER), Chandigarh, India; 8 Govind Ballabh Pant Hospital, New Delhi, India; 9 Sanjay Gandhi Post Graduate Institute (SGPGI), Lucknow, Uttar Pradesh India; 10 King George’s Medical University, Lucknow, India; 11 Dayanand Medical College, Ludhiana, India; 12 Apollo Multispeciality Hospital, Kolkata, India; 13 Seth G S Medical College and K E M Hospital, Mumbai, Maharashtra India; 14 Sir HN Reliance Foundation Hospital, Girgaon, Mumbai, Maharashtra India; 15 Queen Mary Hospital, The University of Hong Kong, Hong Kong, China; 16 University of Southern Denmark, Odense, Denmark; 17 Global Hospital, Mumbai, India; 18 Aga Khan University Hospital, Karachi, Pakistan; 19 BLK-Max Super Specialty Hospital, New Delhi, India; 20 Alka Hospital, Kathmandu, Nepal; 21 Univerity of Barcelona Institut d’Investigacions Biomèdiques August Pi-Sunyer, Barcelona, Spain; 22 Hospital Papa Giovanni XXIII, Bergamo, Italy; 23 Sir Ganga Ram Hospital, Rajender Nagar, New Delhi, India; 24 Kalinga Institute of Medical Sciences (KIMS), Bhubaneshwar, Orissa India; 25 King’s College Hospital, London, UK; 26 Prince-of Song-Kla University, Songkhla, Thailand; 27 Fortis Hospital, Greater Noida, Uttar Pradesh India; 28 Amrita Institute of Medical Sciences and Research Centre, Amrita Vishwa Vidyapeetham, Kochi, India; 29 Christian Medical College (CMC), Vellore, India; 30 University of Louisville School of Medicine, Trager Transplant Center and Jewish Hospital, Louisville, KY USA; 31 Teikyo University School of Medicine, Tokyo, Japan; 32 Centre Hepato-Biliaire, Paul Brousse Hospital, Paris-Saclay University, Villejuif, France; 33 IMS and SUM Hospital, SOA University, Bhubaneswar, Odisha India; 34 Saint Joseph University, Hôtel-Dieu de France University Medical Center, Beirut, Lebanon; 35 Gleneagles Global Hospitals, Hyderabad, Telangana India; 36 The Catholic University of Korea, Seoul, Korea; 37 Aster Medicity, Ernakulam, Kerala India; 38 Capital Medical University, Beijing, China; 39 Nanavati Max Super Specialty Hospital, Mumbai, Maharashtra India; 40 Linkou Chang-Gung Memorial Hospital, Taoyuan, Taiwan; 41 Department of Internal Medicine I, University of Bonn, Bonn, Germany; 42 Linkou Medical Centre, Chang Gung Memorial Hospital, Keelung, Taiwan; 43 University of Alberta, Edmonton, AB Canada; 44 Dr. Cipto Mangunkusumo National General Hospital, Medical Faculty Universitas Indonesia, Jakarta, Indonesia; 45 Medistra Hospital, Jakarta, Indonesia; 46 The Liver Institute, Rajagari Hospital, Aluva, Kerala India; 47 PACE Hospital, Hyderabad, India; 48 Fatima Medical University Hospital, Valenzuela Metro Manila, Philippines; 49 Dr. Rela Institute and Medical Centre, Chennai, India; 50 Hallym University, Chuncheon, Republic of Korea; 51 Korea University College of Medicine, Seoul, Republic of Korea; 52 Sir Salimullah Medical College, Mitford Hospital, Dhaka, Bangladesh; 53 Université de Paris, AP-HP, C, DMU DIGEST, Centre de Référence Des Maladies Vasculaires du Foie, FILFOIE, ERN RARE-LIVER, Centre de Recherche Sur L’inflammation, Inserm, Paris, France; 54 Mansoura University, Mansoura, Egypt; 55 Azienda Ospedaliera Ospedali Riuniti Villa Sofia-Cervello, Palermo, Italy; 56 Clinica La Maddalena, Palermo, Italy; 57 Humanity and Health Medical Center, Hongkong, SAR China; 58 Hospital Médica Sur, Mexico City, Mexico; 59 Instituto Nacional de Ciencias Médicas y Nutrición “Salvador Zubiran”,, Mexico City, Mexico; 60 Latin-American Association for the Study of the Liver (ALEH), Santiago de Chile, Chile; 61 National University Hospital, National University of Singapore, Singapore, Singapore; 62 University of Health Sciences, Ümraniye, Istanbul Turkey; 63 Jigme Dorji Wangchuck National Referral Hospital, Thimphu, Bhutan; 64 Beth Israel Deaconess Medical Center and Harvard Medical School, Boston, MA USA; 65 Taipei Veterans General Hospital, Taipei, Taiwan; 66 School of Medicine, Renji Hospital, Shanghai Jiao Tong University, Shanghai, China; 67 Ernakulam Medical Center (EMC), Kinder Multispeciality Hospital, Kochi, Kerala India; 68 St. John’s Medical College and Hospital, Bangalore, India; 69 Yerevan Medical Scientific Center, Yerevan, Armenia; 70 MIOT International Hospital, Chennai, India; 71 University of Barcelona, IDIBAPS and CIBEREHD, Barcelona, Spain; 72 Storr Liver Centre, Westmead Institute for Medical Research, Westmead Hospital and University of Sydney, Sydney, NSW Australia; 73 Pittsburgh Liver Research Center, University of Pittsburgh Medical Center, Pittsburgh, PA USA; 74 Virginia Commonwealth University, Richmond, VA USA; 75 University of California, San Francisco, San Francisco, CA USA; 76 The Fifth Medical Center of Chinese, PLA General Hospital, Beijing, China; 77 Nanfang Hospital, Southern Medical University, Guangzhou, Guangdong Province China; 78 Medizinische Klinik B, Universitätsklinikum Münster, Münster, Germany; 79 Department of Medicine, Cardinal Santos Medical Center, Manila, Philippines; 80 Gleneagles Global Hospital, Chennai, Tamil Nadu India; 81 Escuela de Medicina, Pontificia Universidad Católica de Chile, Santiago, Chile; 82 Schulich School of Medicine, Western University, London, ON Canada; 83 The First Affiliated Hospital, Zhejiang University School of Medicine, Hangzhou, People’s Republic of China; 84 University of Pennsylvania, Philadelphia, PA USA; 85 King’s College Hospital London, Dubai, United Arab Emirates; 86 Mahatma Gandhi Medical College, Jaipur, Rajasthan India; 87 Max Super Specialty Hospital Saket, New Delhi, India; 88 Chulalongkorn University, Bangkok, Thailand; 89 University of Medicine, Yangon Ministry of Health, Yangon, Myanmar; 90 Government Medical College, Thiruvananthapuram, Kerala India; 91 Liaquat National Hospital, Karachi, Sindh Pakistan; 92 University of Kelaniya, Ragama, Sri Lanka; 93 Bangabandhu Sheikh Mujib Medical University, Dhaka, Bangladesh; 94 Ain Shams University, Cairo, Egypt; 95 Goethe University Clinic Frankfurt, Frankfurt, Germany; 96 Kaohsiung Medical University Hospital, Kaohsiung, Taiwan; 97 College of Medicine, National Sun Yet-Sen University, Kaohsiung, Taiwan; 98 VGM Gastro Hospital, Coimbatore, India; 99 Endocrine and Metabolic Disorder (BIRDEM) Shahbad, Bangladesh Institute of Research and Rehabilitation in Diabetes, Dhaka, Bangladesh; 100 Baylor College of Medicine, Texas Children’s Hospital, Houston, TX USA; 101 İstanbul Health and Technology University, Istanbul, Turkey; 102 Saitama Medical University, Iruma-Gun, Japan; 103 Graduate School of Medicine, Osaka Metropolitan University, Osaka, Japan; 104 Mongolian National University of Medical Sciences, Ulaanbaatar, Mongolia; 105 Graduate School of Medicine, Chiba University, Chuo-Ku, Chiba, Japan; 106 Department of Medicine (DIMED), University of Padova, Padua, Italy; 107 Zydus Hospital, Ahmedabad, Gujarat India; 108 Mayo Clinic College of Medicine and Science, Rochester, MN USA; 109 Mercy Medical Center, Baltimore, USA; 110 All India Institute of Medical Sciences (AIIMS), New Delhi, India; 111 Topi Wala National (TN) Medical College and BYL Nair Charitable Hospital, Mumbai, India; 112 Tongji Medical College, Huazhong University of Science and Technology, Wuhan, China; 113 Changi General Hospital, Singapore, Singapore; 114 European Foundation for the Study of Chronic Liver Failure (EF CLIF), Barcelona, Spain; 115 Centre de Recherche Sur L’Inflammation (CRI), INSERM and Université Paris-Cité, Paris, France; 116 Assistance Publique-Hôpitaux de Paris (APHP), Hôpital Beaujon, Service d’Hépatologie, Clichy, France; 117 University of California, San Diego, La Jolla, CA USA; 118 Amrita Institute of Medical Sciences and Research Centre, Faridabad, Haryana India; 119 University of Malaya, Kuala Lumpur, Malaysia; 120 Deenanath Mangeshkar Hospital and Research Centre, Pune, India; 121 Phramongkutklao Hospital and College of Medicine, Bangkok, Thailand; 122 Violeta Medical Centre, Yerevan, Armenia; 123 Midas Multispeciality Hospital Pvt. Ltd, Nagpur, Maharashtra India; 124 Digestive Health Institute, Children’s Hospital, Aurora, CO USA; 125 Kasturba Medical College, Manipal Academy of Higher Education, Manipal, Karnataka India; 126 National Institute of Medical Sciences, Jaipur, India; 127 University of Maryland School of Medicine, Baltimore, USA; 128 Royal Prince Alfred Hospital, Sydney, Australia; 129 Selayang Hospital, University of Malaysia, Batu Caves, Selangor, Malaysia; 130 Aster CMI Hospital, Bengaluru, Karnataka India; 131 KIIT University, Fortis Escorts Hospital, Okhla, New Delhi, India; 132 Bir Hospital, National Academy of Medical Sciences, Kathmandu, Nepal; 133 Sawai Man Singh (SMS) Medical College and Hospital, Jaipur, Rajasthan India; 134 Baylor Simmons Transplant Institute, Dallas, TX USA; 135 Dr Sampuranand Medical College (SNMC), Jodhpur, Rajasthan India; 136 Pandit Bhagwat Dayal Sharma Post Graduate Institute of Medical Sciences, Rohtak, Haryana India; 137 Graduate School of Medical and Dental Sciences, Niigata University, Niigata, Japan; 138 Siriraj Hospital, Mahidol University, Bangkok, Thailand; 139 Astana Medical University, Astana, Kazakhstan; 140 Mazumdar Shaw Medical Centre, Bangalore, Karnataka India; 141 Faculty of Medicine, The Chinese University of Hong Kong, Hong Kong, China; 142 Punjab Institute of Liver and Biliary Sciences, Mohali, Punjab India; 143 Indira Gandhi Institute of Medical Sciences, (IGIMS), Bely Road Patna, Bihar, India; 144 Stanford University, Stanford, CA USA; 145 Medical School, Zhongda Hospital, Southeast University, Nanjing, China; 146 Seoul National University College of Medicine, Seoul, Republic of Korea; 147 Ziauddin University Hospital Karachi, Karachi, Pakistan; 148 Juntendo University, Tokyo, Japan; 149 Changgung Hospital, Tsinghua University, Beijing, China; 150 Yamanashi Central Hospital, Yamanashi, Japan

**Keywords:** AARC, ACLF, Acute decompensation (AD), Cirrhosis, Non-acute decompensation (NAD), Organ failure, Recompensation

## Abstract

Acute-on-chronic liver failure (ACLF) is a condition associated with high mortality in the absence of liver transplantation. There have been various definitions proposed worldwide. The first consensus report of the working party of the Asian Pacific Association for the Study of the Liver (APASL) set in 2004 on ACLF was published in 2009, and the “APASL ACLF Research Consortium (AARC)” was formed in 2012. The AARC database has prospectively collected nearly 10,500 cases of ACLF from various countries in the Asia–Pacific region. This database has been instrumental in developing the AARC score and grade of ACLF, the concept of the ‘Golden Therapeutic Window’, the ‘transplant window’, and plasmapheresis as a treatment modality. Also, the data has been key to identifying pediatric ACLF. The European Association for the Study of Liver-Chronic Liver Failure (EASL CLIF) and the North American Association for the Study of the End Stage Liver Disease (NACSELD) from the West added the concepts of organ failure and infection as precipitants for the development of ACLF and CLIF-Sequential Organ Failure Assessment (SOFA) and NACSELD scores for prognostication. The Chinese Group on the Study of Severe Hepatitis B (COSSH) added COSSH-ACLF criteria to manage hepatitis b virus-ACLF with and without cirrhosis. The literature supports these definitions to be equally effective in their respective cohorts in identifying patients with high mortality. To overcome the differences and to develop a global consensus, APASL took the initiative and invited the global stakeholders, including opinion leaders from Asia, EASL and AASLD, and other researchers in the field of ACLF to identify the key issues and develop an evidence-based consensus document. The consensus document was presented in a hybrid format at the APASL annual meeting in Kyoto in March 2024. The ‘Kyoto APASL Consensus’ presented below carries the final recommendations along with the relevant background information and areas requiring future studies.

**Supplementary Information:**

The online version contains supplementary material available at 10.1007/s12072-024-10773-4.

## Introduction

Acute-on-chronic liver failure (ACLF) is recognized globally as a severe ailment associated with high short-term mortality in patients with chronic liver disease and cirrhosis. Multiple definitions of ACLF exist, each proposed by four major consortia, including the APASL ACLF Research Consortium (AARC), the European Association for the Study of the Liver-Chronic Liver Failure (EASL-CLIF), North American Association for the Study of the End Stage liver Disease (NACSELD), and Chinese Group on the Study of Severe Hepatitis B (COSSH); and one from the society: the American Association for the Study of liver disease (AASLD). Additionally, national societies in Japan, Korea, China, and Mexico have developed their own definitions [1–7]. These variations have led to challenges in the diagnosis, management, and research of ACLF due to inconsistencies across regions and populations. While there has been significant progress in the understanding of this acutely fatal disease, the treatment is largely limited to bridge therapies and liver transplantation (LT). However, access to LT is restricted for many patients, highlighting the urgent need for collaborative research focused on non-transplant therapeutic strategies. One of the major hindrances to improving the outcomes of these sick patients is the lack of a unified definition. In response to this challenge, the APASL, through its ACLF Research Consortium (AARC), initiated a global collaboration to develop a new consensus on ACLF that would be relevant to patients worldwide. The AARC invited scientists and opinion leaders from around the world, including those not affiliated with any existing consortium, to contribute unbiased perspectives. A systematic, multilayered methodology was employed to identify and address the most critical and controversial aspects of ACLF. A unified definition is crucial for research in liver-directed therapies, which can lead to improved outcomes for these sick patients. Based on a series of virtual meetings, 16 important questions and areas were identified. Subsequently, the experts for each area were selected from various countries for global representation. Accordingly, data collection and draft preparation for each of the sections were undertaken. Each section had three members in charge of drafting. Every section was further divided into several subgroups, each of which was led by a senior investigator and two experts. The level of evidence (LoE) and grade of recommendations were based on the GRADE system [8, 9] (Table 1). The documents and the recommendations were prepared by the section chiefs after detailed deliberations with the members. These recommendations were then collated and circulated to the entire group. These recommendations were thoroughly reviewed, refined, and presented at the APASL Annual Conference in Kyoto in March 2024. The result is the Kyoto Consensus, a globally unified framework for ACLF that incorporates the collective knowledge and expertise of nearly 200 scientists from five continents.

**Table 1:** Level of evidence and grade of recommendation used for the guideline development

	Level of evidence ^a,b^	Confidence in the evidence
High	Information obtained from meta-analyses or systematic reviews, or from numerous randomized trials that have high-quality data	It is improbable that additional research will significantly alter our level of confidence in the assessment of potential benefits and risks
Moderate	Information obtained from either a singular randomized controlled trial (RCT) or various non-randomized studies	Additional research, if conducted, may potentially alter our estimation of the benefit and risk and have an impact on our level of confidence in the estimate
Low	Studies of limited sample size, observational studies conducted retrospectively, and registries	There is a degree of uncertainty associated with any estimate of the effect
	Recommendations – Grade ^c^	Wording associated with the grade of recommendation
Strong	The strength of the recommendation was influenced by several factors, such as the quality of the evidence, the presumed outcomes that are important for the patient, and the cost implications	“must”, “should”, or “we recommend
Weak	The recommendation may be made with less certainty and may result in higher costs or resource consumption due to variability in preferences and values, or increased uncertainty	“can”, “may”, or “we suggest

^a^To make the GRADE system more objective, the type of studies from which the evidence are derived have been mentioned in the Level of Evidence

^b^Level was graded down if there was a poor quality, strong bias or inconsistency between studies; level was graded up if there was a large effect size

^c^Recommendations reached by consensus of the members and included the quality of evidence, presumed patient-important outcomes and costs

## The concept of hepatic reserve and the liver failure

### Natural history of Cirrhosis and decompensation and causes of death

**Fig. 1:**
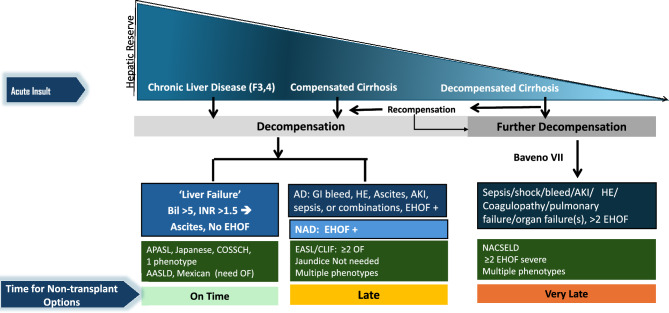
Natural History and Outcomes of CLD and cirrhosis based on hepatic reserve, nature and degree of acute insult. Abbreviations: OF- organ failure, EHOF- extrahepatic organ failure, NAD- non-acute decompensation

The natural history of cirrhosis is divided into two distinct stages; the compensated and the decompensated stage. The compensated stage is an asymptomatic period with a median survival of 10 or more years. The second stage, known as decompensated cirrhosis, is characterized by the presence of symptoms and a median survival of 1–2 years. The term “decompensated cirrhosis” is typically used to describe the presence or history of specific conditions, such as ascites, bleeding, hepatic encephalopathy (HE), or jaundice (Fig. 1), and broadly denotes hepatic reserves and functions which are unable to maintain the required body functions. The transition from compensated to decompensated cirrhosis leads to increased mortality risk [10–12]. A recent study by D’Amico and colleagues evaluated the impact of further decompensation in patients with decompensated cirrhosis [13]. The term decompensation here included bilirubin > 3 mg/dl, clinically detectable ascites, overt HE, or portal hypertensive bleeding. ‘Further decompensation’ was defined as: (a) second portal hypertensive-driven decompensating event (ascites, HE or acute variceal bleed [AVB]), and/or jaundice; (b) recurrent variceal bleeding, worsening ascites (requirement of ≥ 3 Large volume paracentesis (LVP) within 1 year), recurrent encephalopathy, and (c) development of spontaneous bacterial peritonitis (SBP) and/or acute kidney injury (AKI)/hepatorenal syndrome. The last three complications were considered as further decompensating events because they only occurred after the development of ascites [13]. Therefore, jaundice is considered a decompensating event in the natural history of cirrhosis. In recent years, increasing attention has been given to new entities whose roles in the natural history of liver cirrhosis remain to be characterized, namely, first decompensation, acute decompensation (AD), ACLF, Non-AD (NAD), and further decompensation and recompensation [14]. In a recent study from the US in 2018, there were 85,807 deaths from underlying chronic liver disease (CLD). Liver-related mortality was the leading cause of death for all types of CLD followed by death from cardiac causes [15].

A retrospective cohort study of 202,022 adult patients with cirrhosis from 2000 to 2017 reported compensated cirrhosis in 73% and decompensated in the remaining. After a median follow-up of 5 years, 81,428 (40%) patients died. Irrespective of the type of index decompensation (jaundice, ascites, HE, bleeding), the mortality at 10 years was not more than 45%, implying decompensated cirrhosis has better outcomes than ACLF who have high short-term mortality of up to 50% at 3 months. Furthermore, *liver-related deaths* accounted for up to 62% of patients with decompensated cirrhosis but only up to 17% with compensated cirrhosis. The most common cause of death was liver-related (32%), followed by non-hepatic malignancy (19%), and cardiovascular disease (CVD)-related causes (17%). Patients with compensated cirrhosis suffered more from non-liver-related deaths, such as non-hepatic malignancy or CVD-related death. Conversely, among those with either decompensated cirrhosis and/or hepatocellular carcinoma (HCC), liver-related deaths were the most common cause of death [16]. The cumulative mortality for patients with compensated cirrhosis was 9.5%, 19.6%, and 31% at 1, 5, and 10 years, respectively, compared to 30.6%, 56.8%, and 72.6% for those with decompensated cirrhosis [16].

### Further decompensation in a decompensated cirrhosis- defining AD, ACLF and decompensated cirrhosis

**Fig. 2:**
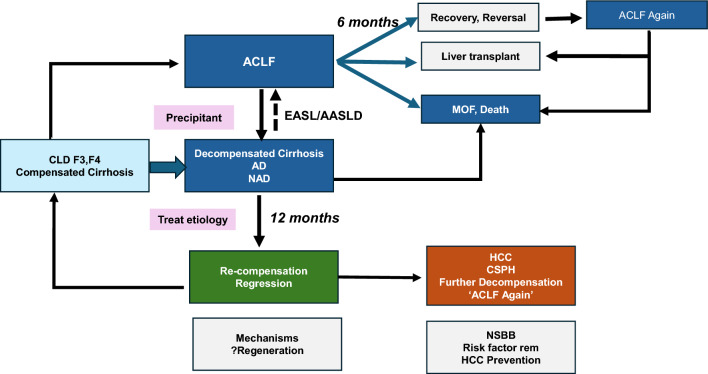
The spectrum of liver injury and liver failure in a patient of Cirrhosis

CLD is a progressive disease characterized by a gradual decline in liver function, including impairment in the synthesis of proteins and clotting factors, detoxification, and/or energy metabolism for more than 6 months [17]. The reason for this can be multiple, and the disease, when diagnosed, can be at any stage of fibrosis spectrum ranging from F1 to F4. Cirrhosis is the end-stage of fibrosis which is usually irreversible. Patients with cirrhosis are bound to develop complications of portal hypertension. AD is the development of either jaundice, ascites, HE, variceal bleeding, and/or infection, which requires medical attention but may not require hospitalization. Approximately 2/3rd of patients with cirrhosis have only one event of decompensation, and 1/3rd develop more than one event. [18] The time to develop these complications and the sequence is never uniform. Experts agree that the *time frame for the development of decompensation should be standardized to 4 weeks* (Table 2). Further decompensation refers to a stage in which the patient experiences a new episode of decompensating events, carrying an additional risk for mortality. A multicenter study in the European-Latin American region confirmed that additional decompensation events, whether single or multiple, negatively affect survival in patients who already have ascites as their first acute decompensation [19]. The natural course and outcome of patients with AD and ACLF vary depending on the nature and severity of the precipitating event and the stage of underlying liver disease [20, 21] (Fig. 2). In contrast, non-acute decompensation (NAD) describes a more gradual form of decompensation, in which symptoms develop slowly, often without the need for immediate hospitalization (Table 2).

**Table 2:** Spectrum of decompensation in CLD/cirrhosis

Parameter	Non-Acute Decompensation (NAD)	Acute Decompensation (AD)	Further Decompensation	ACLF
Acute insult	No	Yes	Yes	Yes
Identifiable insult	–	Around 40%	^*^Always	70%
Time from acute insult to presentation	–	Up to 3 months	Up to 3 months	Up to 28 days
Underlying cirrhosis	Always diagnosed	Diagnosed or undiagnosed	Always diagnosed	CLD with F3-F4 Fibrosis or Cirrhosis Diagnosed or undiagnosed
Prior decompensation	Not always	Not always	Always	^#^No
1-, 3-month mortality	10–15%	45–60%	45- 60%	30–70%
Regression/Recompensation	^@^Often	Limited	Less common	Often
Bridge therapy	Not needed	Limited use	Limited use	Often helpful

*NAD* non-acute decompensation, *AD* acute decompensation, *ACLF* acute-on-chronic liver failure

^*^Acute insult may or may not be detected. But it often leads to further decompensation in AD or NAD. # Prior decompensation is an exclusion for defining as per APASL. Other definitions inclusive of this. @Regression/Recomepenasation

### Recompensation in cirrhosis: the concept and implication for diagnosis of ACLF

The concept of recompensation in cirrhosis was introduced in Baveno VII and has since emerged as a significant pivot in the natural history of liver disease, suggesting the potential for regression from decompensated to a compensated or recompensated state [22, 23]. The recompensated phase is arbitrarily defined as a state wherein patients, due to effective control of etiology and management of complications, show an absence of clinical decompensation events for an extended period, with laboratory and clinical features akin to compensated cirrhosis [23]. The implications of recompensation for the diagnosis and prognosis of ACLF are profound and multifaceted. Firstly, it challenges the traditional perspective of decompensated cirrhosis as an irreversible endpoint, providing a new paradigm for patient management and prognosis. The identification of recompensation is contingent upon strict criteria, including the removal or control of etiology, resolution of clinical decompensations such as ascites and HE, the absence of variceal bleeding, and sustained improvement in liver function tests such as albumin, bilirubin, and the international normalized ratio. The recompensation concept stems from observational studies and clinical experiences indicating a sustained suppression or cure of the underlying etiology of liver disease, such as viral eradication in hepatitis C, can lead to the reversal of decompensated cirrhosis [24]. Similarly, abstinence from alcohol in patients with alcohol-related liver disease (ALD) and effective control of viral load in hepatitis B can lead to significant clinical improvements, including the resolution of ascites and HE, further supporting the possibility of recompensation [25]. The incidence of compensation rates has been variably reported based on etiology (18% in alcohol-related cirrhosis and 25% in HCV cirrhosis) [26, 27]. The pathophysiology of recompensation is still not fully understood but is believed to involve the reversal of processes that drive decompensation. These processes may include the reduction of portal hypertension, amelioration of systemic inflammation, improvement in gut permeability, and resolution of cirrhosis-associated immune dysfunction [22–24]. An enhanced understanding would provide a roadmap for therapeutic interventions to achieve recompensation, which includes treatments targeting specific etiologies of liver disease, anti-inflammatory, immune modulation, liver regeneration, and portal hypertension.

The current Baveno VII definition of recompensation mentions that the patient should remain compensated without the use of non-selective beta blockers. This is however, at variance with several existing guidelines which recommend the use of NSBB for prolonged periods for secondary prophylaxis of variceal bleeds. Additional clarity is required in this concept. In the definition of the recompensation, there should be inclusion of regression of the degree of portal hypertension whether spontaneously or while on NSBB. *Stopping NSBB should not be made a criterion for defining recompensation* as portal hypertension drives the future decompensating events. Portal hypertension could be assessed by HVPG or using non-invasive tools.

A recent study from China among 461 ACLF patients showed lesser severity, lower incidence of pulmonary failure, and better 1-year survival in patients developing ACLF with baseline recompensated cirrhosis as compared to patients with decompensated cirrhosis [28]. Importantly, clinical features and prognosis were similar between ACLF patients with recompensated and compensated cirrhosis [28]. On the flip side, recent studies from the AARC consortium and India revealed resolution of ACLF after an index ACLF event is possible and improves the prognosis with almost zero short-term mortality [29, 30]. Such improvements in disease course critically influence the management decisions such as delisting from liver transplantation. Nevertheless, there remains a continued need for surveillance for complications like hepatocellular carcinoma and recurrence of ACLF in these patients. Further studies should aim to delineate the natural history of recompensated cirrhosis, explore non-invasive biomarkers of recompensation, and elucidate the molecular mechanisms underlying the reversal of decompensation.

### Reversal of ACLF syndrome: timeline, predictor(s) and outcome

In the context of ACLF, it would be better to use the term ‘reversal of ACLF’ than recompensation, as a proportion of the patients may have advanced fibrosis and not cirrhosis as the underlying liver disease and to avoid confusion with recompensation of decompensated cirrhosis. While the current LoE for reversal of ACLF is still evolving, the need for careful prospective studies is evident. AARC defines the reversibility as a decrease of bilirubin below 5 mg/dL and reversal of coagulopathy to INR below 1.5 and no encephalopathy with or without resolution of ascites. From the AARC database, about 70% of the survivors had a reversal of ACLF by day 90, which was maintained for 1 year. The median time to reversal of syndrome, i.e., jaundice and coagulopathy, was 30 days. Baseline albumin, AARC score, and transient elastography predicted long-term reversibility [1]. Similarly, patients with HBV-related ACLF who achieve reversal of ACLF remain stable for up to 5 years [31]. Another multicenter study including 346 patients with ACLF from India reported complete reversal of ACLF among 47% of patients compared to 73% who received beta blockers [30]. The mean time to reversal was 2.3 months in the beta-blocker group compared to 2.66 months in those not receiving beta-blockers [30]. These patients, however, remain at risk of decompensation (noted in 5–20%) and HCC (noted in 4–12%) even after reversal [31]. In the CANONIC study, which had included decompensated cirrhosis, among patients with initial ACLF-1, the most frequent clinical course was resolution of the syndrome in 54.5%; the reversal rate decreased to 34.6% for baseline ACLF-2 and 16% for ACLF-3 [32]. The median time for resolution was 14 (1–28) days. Patients who recover from grade 1 ACLF have an increased risk of later developing Grade 3 ACLF compared to those who never experience grade 1 ACLF [33]. Reversible decompensations for grade 1 ACLF have a lower risk of progressing to grade 3 ACLF, while liver-related organ failures have a higher risk of grade 3 ACLF [33]. Irrespective of the definition, reversal of ACLF is possible, and those who achieve reversal have improved long-term survival [31].

The timeline and sequence of reversal were deliberated upon in detail. There was no data to identify the sequence of reversal, which needs to be assessed in future studies. However, with respect to time, the complete resolution would not be more than 6 months, and those achieving resolution by 90 days would be named as an “early reversal”. Some experts also suggested that 30 days should be named as early as most patients would have achieved final grade by then. However, such studies have not counted for the time from diagnosis to final grade; instead, they focused on time from medical attention to final grade [32]. Reversal of ACLF syndrome remains to be studied more and hopefully prospective data in the future can indicate predictors of reversal and the possibility of retaining the native liver.

### Regression of Cirrhosis- etiology, time frame, and implication for ACLF

A considerable reduction in fibrosis characterizes the regression of advanced fibrosis/compensated and early stage of cirrhosis. Extensive evidence supports the efficacy of etiology-specific treatment for chronic liver disease, demonstrating marked regression of liver fibrosis and, in some cases, reversal of compensated cirrhosis. Data is robust about chronic hepatitis B; long duration (> 1 year) of antiviral therapy resulting in viral load suppression has led to fibrosis regression and reversal of compensated cirrhosis in 27–88% [34] and 33–80%, respectively [35]. Marcellin et al. reported through paired liver biopsy assessment among 348 patients who were treated with TDF over 240 weeks, that 51% of these patients had regression of fibrosis, whereas 74% with baseline cirrhosis achieved reversal of cirrhosis [36].

Hepatitis C treatment with IFN-based regimens and direct-acting antivirals (DAAs) treatment showed reversal of cirrhosis among 24–83% of patients who achieved SVR [35, 37]. Pooled data from 4 RCTs by Poynard et al. demonstrated a reversal of cirrhosis in 49% with Interferon-based therapy [38]. Another similar meta-analysis reported a higher probability of a reversal of cirrhosis among those who achieved SVR [39]. Even patients with compensated cirrhosis, particularly those with subclinical portal hypertension who achieve SVR, have been noted to have a significant reduction in hepatic venous pressure gradient (HVPG) [37].

**Fig. 3:**
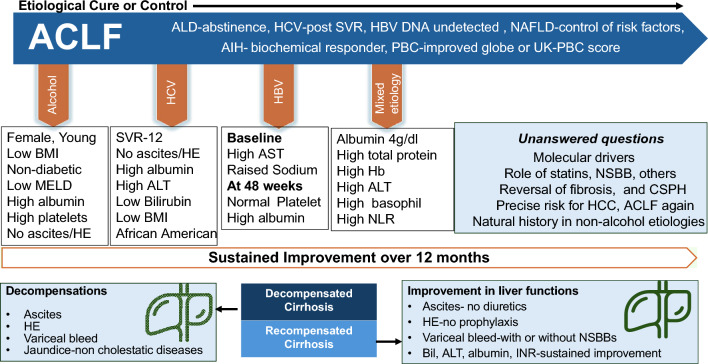
Recompensation of Cirrhosis after ACLF syndrome or Acute Decompensation

Effect of exercise with or without diet modification among MAFLD/MASLD patients shows improvement of NAS score [40]. Significant reduction in fibrosis among NASH/MASH patients with advanced fibrosis and compensated cirrhosis can occur in 26% with anti-fibrotic therapies such as FXR, PPAR, and anti-diabetic agents over 48 weeks of therapy [41]. Similarly, treatment of AIH and PBC with immunosuppressant (prednisolone ± azathioprine) and ursodeoxycholic acid, respectively, for at least 2 years has led to significant improvement in fibrosis score and even reversal of cirrhosis [34]. Data on the regression of fibrosis among ALD after alcohol abstinence is lacking but is associated with clinical improvement (lower risk of decompensation and improved survival) among all stages of cirrhosis [42] (Fig. 3).

Studies that utilized both liver biopsy and transient elastography to assess histological outcome post-treatment in chronic viral hepatitis concluded that there is a tendency of overestimation of regression of cirrhosis and fibrosis stage with TE [43]. The improvement of liver stiffness is more a reflection of the resolution of inflammation rather than the regression of fibrosis. Hence, a longer duration of post-treatment follow-up is required for the non-invasive fibrosis assessment to be a reliable marker of fibrosis regression. Long-term studies on improvement in liver stiffness to demonstrate the reversibility of cirrhosis or fibrosis in ALD are lacking. Current strategies to regress fibrosis in patients with MAFLD/MASLD are underway.

**Fig. 4:**
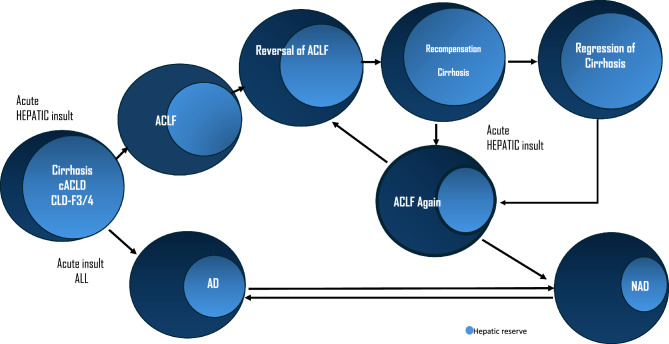
The concept of hepatic reserve and reversal, regression of ACLF and the “ACLF Again”

Reversal of advanced fibrosis/cirrhosis is likely to decrease the risk of progressive liver disease and, thus, ACLF. Thus, global efforts at identifying patients who are candidates for specific treatments and enhancing etiology-specific treatments, are likely to decrease the pool of patients at risk for ACLF. The main determinant of the reversal of ACLF and of recompensation of cirrhosis is the hepatic reserve (Fig. 4). There is a need for quantitative liver function tests in the presence of liver failure and decompensation.

1. **Recommendations: the concept of ACLF and Hepatic reserve**
  1.1. Natural history of cirrhosis and decompensation and causes of death
    1.1.1. There are two stages of cirrhosis: compensated and decompensated. (LoE-High, Recommendation-Strong).
    1.1.2. Decompensation is defined as the development of ascites, variceal bleeding, or HE in patients with cirrhosis (LoE: High, Recommendation: Strong).
    1.1.3. Mortality at 1, 5 and 10 years was 9.5%, 19.6% and 31% and 30.6%, 56.8% and 72.6%, respectively among patients with compensated and decompensated cirrhosis, which is lower than ACLF. (LoE-Moderate, Recommendation-Strong).
    1.1.4. The most common cause of mortality is liver-related decompensated cirrhosis. (LoE-High, Recommendation-Strong).
  1.2. Further Decompensation in a Decompensated Cirrhosis- Defining AD, ACLF and ESLD
    1.2.1. AD is the acute development of complications of cirrhosis like rapid development of ascites, gastrointestinal bleeding, and HE within four weeks of onset and requiring non-elective medical attention. (LoE-Moderate, Recommendation-Strong).
    1.2.2. Further decompensation is any episodes of AD in patients with prior decompensation (LoE-Moderate, Recommendation-Strong).
    1.2.3. Patients with AD or further decompensation have high mortality in the absence of recovery or liver transplantation in 3 months. (LoE-Moderate, Recommendation-Strong).
    1.2.4. Patients with AD and organ failure(s) have very high short-term mortality. (LoE-Moderate, Recommendation-Strong).
    1.2.5. The differentiating features between the various presentations of AD and ACLF should be carefully examined by including AARC, NACSELD, EASL-CLIF, COSSH, and various other databases. (LoE-Low, Recommendation-Weak).
  1.3. Recompensation in cirrhosis- The Concept and Implication for Diagnosis of ACLF
    1.3.1. Re-compensation can be defined as the removal or control of etiological factor(s) with sustained absence of decompensating events for ≥ 1 year in patients with decompensated cirrhosis coupled with improvement in liver function tests (LoE: Low; Recommendation: Weak).
    1.3.2. Removal or control of inciting etiology in defining re-compensation, which pertains to alcohol abstinence in alcohol-associated liver disease, suppression of viral load in hepatitis B, sustained virological response in hepatitis C, suppression of disease activity in autoimmune disease, and control of metabolic comorbidity in MAFLD/MASLD (LoE: High, Recommendation: Strong).
    1.3.3. The period during which there should be sustained absence of decompensation without treatment (diuretics for ascites, lactulose ± rifaximin for HE) should be at least one year. (LoE: Moderate; Recommendation: weak)
    1.3.4. Sustained reduction of portal pressure of 10% or more from the baseline should be achieved during the 12-month period. Non-invasive tools should be used for repeated portal pressure assessment. (LoE: Low; Recommendation: Weak)
  1.4. Reversal of ACLF syndrome- timeline, predictor, and outcome
    1.4.1. Reversal of ACLF is the reversal of parameters used for defining the syndrome ACLF. (LoE- Low; Recommendation-Strong)
    1.4.2. Patients with ACLF have high 28-day and 90-day mortality, with improved outcomes beyond 90 days. (LoE-Moderate, Recommendation-High).
    1.4.3. The timeline for reversal of ACLF syndrome may be considered at 6 months. Patients achieving reversibility by 90 days may be considered “early reversals”. (LoE-Low, Recommendation-Weak).
    1.4.4. Predicting a good outcome after ACLF, involves a combination of biomarkers, disease severity scores, precipitating cause, hepatic reserve, extrahepatic augmenting factors, reduction of portal hypertension (with or without beta-blockers), regression of organ failure(s), and unfavorable clinical indicators. (LoE-Moderate, Recommendation-Strong).
    1.4.5. The baseline ACLF grade and any shift in the grade in the first week can identify patients who are likely to reverse. (LoE-Low, Recommendation-Strong).
    1.4.6. Reversal of ACLF syndrome is an important therapeutic goal, and it can be considered for delisting patients from the transplant waitlist. (LoE-Low, Recommendation- Strong)
  1.5. Regression of cirrhosis- the etiology, time frame and implication for ACLF
    1.5.1. Successful etiology-specific treatment of chronic liver disease leads to regression of advanced fibrosis and even reversal of compensated cirrhosis. (LoE-High, Recommendation-Strong).
    1.5.2. Achieving SVR post-hepatitis C treatment, viral suppression in CHB, and biochemical remission in AIH and PBC significantly improves the probability of regression of advanced fibrosis and reversal of decompensated cirrhosis (LoE-Moderate, Recommendation-Strong).
    1.5.3. Alcohol abstinence plays an important role in avoiding/delaying liver-related events in ALD, but data on histological improvement post-abstinence in ALD is limited. (LoE-Moderate, Recommendation-Strong).
    1.5.4. Lifestyle modifications, medical, endoscopic, and surgical therapies for obesity, and therapies targeted at NASH with fibrosis demonstrate promising results in terms of improvement of NAS score and fibrosis regression, but more data is required. (LoE-Moderate, Recommendation-Weak).
    1.5.5. A longer duration of treatment increases the probability of regression of advanced fibrosis and reversal of compensated cirrhosis. (LoE-Low, Recommendation-Weak).
    1.5.6. Interpretation of LSM via transient elastography to assess fibrosis regression and reversal of cirrhosis should be made with caution, as there is a tendency for an overestimation in the improvement of the fibrosis stage, compared to liver biopsy. (LoE-Low, Recommendation-Weak).
    1.5.7. Reversal of cirrhosis/fibrosis may not preclude the development of ACLF. (LoE-Low, Recommendation-Weak)

## Infection and/or sepsis in the natural history of chronic liver disease

### Infection leading to organ failure in cirrhosis with or without liver failure

Infection in patients with cirrhosis is known to precipitate organ failures [44]. In patients with cirrhosis, factor VIII levels are elevated, while other features such as coagulopathy, thrombocytopenia, and elevation in D-dimers are frequent even in the absence of sepsis. There are several similarities between cirrhosis and sepsis with respect to hemodynamics and the impact on the coagulation system. Infection can precipitate organ failure and, even in the absence of hepatic failure, is associated with high mortality [3]. Thirty-day survival in patients with zero, one, two, three, and four extrahepatic organ failure is 90%, 72.6%, 51.3%, 36%, and 23%, respectively, for sepsis-precipitated organ failure. Sepsis-precipitated organ failure is largely driven by inflammation [45, 46]. Bacterial infections have been reported in the West as a major precipitant of ACLF [2, 3]. The common types of bacterial infections include SBP, urinary tract infection (UTI), pneumonia, spontaneous bacteremia, and skin and soft tissue infection. In a recent study that investigated regional differences in bacterial infection-related ACLF in patients with cirrhosis admitted to the hospital, 230 patients developed ACLF after diagnosis of infection, resulting in an overall rate of bacterial infection related-ACLF of 48%, with rates differing amongst different geographic regions (38% in Southern Europe vs. 75% in the Indian subcontinent). Bacterial infection related-ACLF more frequently developed in younger patients (55 ± 13 vs. 58 ± 14 years), males (73% vs. 62%), patients with alcohol-related cirrhosis (59% vs. 45%), and those with a higher baseline MELD (25 ± 11 vs. 16 ± 5) (*p* < 0.001). These studies defined ACLF as per EASL-CLIF criteria [45]. Early identification of infection and timely and judicious use of antibiotics can prevent extrahepatic organ failures and improve survival [47, 48].

### Hepatotropic and non-hepatotropic infections leading to ACLF

Liver failure in both CLD and cirrhosis can be caused by both hepatotropic and non-hepatotropic viruses. HBV is a common cause of acute hepatic insult in the Asia Pacific region. HBV reactivation, ALF due to HBV, and ACLF due to HBV are marked by subtle differences. While reactivation may have elevated enzymes with or without jaundice, as per AASLD, ALF patients can present with HE up to 26 weeks from the onset of jaundice and this is an exception for ALF definition where underlying liver disease can exist [49]. On the contrary, those with ACLF have ascites with or without HE and are more prone to secondary bacterial infections and organ failures. Irrespective of the type of presentation, early initiation of antiviral therapy is crucial [50]. Currently, no studies have identified predictors of ACLF and ALF development in patients with HBV reactivation. It is also unknown if disease-modifying agents or early initiation of antivirals can prevent the development of ALF/ACLF. It is important to differentiate ALF (or acute viral hepatitis) from ACLF due to HBV reactivation (Table 3).

**Table 3:** Differences between Acute liver failure/hepatitis vs. ACLF due to HBV reactivation

Variable	Acute liver failure/acute hepatitis B	ACLF due to HBV reactivation
Jaundice to encephalopathy interval	4 weeks (AASLD defines as 26 weeks)	4 weeks
Stigmata of chronicity of liver disease	No	± ascites with or without splenomegaly
HBV DNA levels	Lower	Higher
Concomitant bacterial infections	Usually very late	Early in the course/may be superimposed
Spontaneous recovery	Not uncommon	Rare
Antiviral	Unclear	Definitive role
Liver Transplant	Emergent	Urgent

The literature documenting hepatotropic viruses (A–E) superimposed on either CLD or cirrhosis is quite clear, and they portend a poor prognosis, including the development of ACLF [51–53]. Hepatitis B reactivation is a common cause of ACLF, especially in Asian countries [54]. The HBV re-activation could be spontaneous or induced by either chemotherapy, withdrawal of antivirals, or in the presence to control of con-commitment viral infection [55]. Studies primarily from the Indian subcontinent have also reported HAV superinfection as a common etiology (42%) of ACLF amongst children [56]. In another study, acute precipitant for ACLF in adults reported HEV, HAV, or both in 61%, 27%, and 6% of cases, respectively [53]. Similarly, reactivation of hepatitis C virus infection has also been reported, especially after immune suppressive therapy, and is rarely noted in clinical practice [57]. SARS-CoV2 virus might lead to liver failure and has been reported to trigger ACLF in patients with cirrhosis [58, 59]. Apart from viral and bacterial infectious agents, parasitic and fungal infections may affect the liver and may act as acute hepatic insults leading to ACLF, but the data on these infections are limited.

### Laboratory and clinical diagnosis of infection in cirrhosis with or without decompensation

Infections in cirrhosis are a driver of mortality and morbidity in the disease [60]. Cultures are commonly used to diagnose infections. However, in cirrhotic patients, only 57% presented with infection had positive cultures [61]. Routine examination of blood body fluids may help in detecting infections early. Infections can be challenging to diagnose in patients with liver cirrhosis. Only 56% of patients with liver cirrhosis having active infections manifest with fever as compared to 85.5% in non-cirrhotic patients [62]. As such, inflammatory markers such as serum CRP and procalcitonin have been proposed to aid in the diagnosis of cirrhosis. CRP has been shown to have questionable value in patients with cirrhosis. A systematic review demonstrated that moderate CRP increases may not predict infections in cirrhosis, but high CRP levels on presentation or persistently high CRP levels may provide better evidence of infection in these patients [63]. A subsequent single-center study, however, demonstrated a cutoff level of > 10 mg/L having an AUC of 0.82, with cutoffs dropping with higher Child–Pugh stages of cirrhosis [62]. Procalcitonin has been shown to have moderate diagnostic value for bacterial infections in cirrhosis, with an AUC of 0.80 [64]. Additionally, serum procalcitonin has been shown to be a good prognostic indicator for the course of hospitalization and could be associated with the grade of ACLF triggered by infections [65]. Leukocyte ratios such as monocyte–lymphocyte ratio (MLR) and neutrophil–lymphocyte ratio (NLR) have also been shown to be useful in predicting infections and deteriorations [66].

Fungal infections are present in approximately 4% of patients with cirrhosis as culture positive, with invasive candida as the most common (70–90%) followed by invasive aspergillosis (10–20%) [61, 67]. It is imperative to pick these infections up early. 1,3-β-D-glucan can be detected in an antigen-based assay and has a good negative predictive value assay and a lower limit of detection at 2.16 pg/mL [68]. Other novel tools to detect candida bloodstream infections include the T2Candida panel—however, data are limited in cirrhosis. The serum galactomannan helps detect invasive aspergillosis, with a sensitivity of 33–38%, and a specificity of 87–97% [67]. These biomarkers have limited accuracy at best, and data is still mixed to the value. While they are easily available, it would still be prudent to have a low threshold for investigating infections based on the clinical status of the patient. Any deterioration in the patient like new onset AKI, altered mental status, or new onset organ dysfunction/failure should trigger a search for infections [46].

It is challenging to diagnose sepsis (SIRS with proven infection) in cirrhotic patients. SIRS is defined by meeting any of the following two: (i) Body temperature over 38 or under 36 degrees Celsius; (ii) Heart rate greater than 90 beats/minute; (iii) Respiratory rate greater than 20 breaths/minute or partial pressure of CO_2_ less than 32 mmHg; or (iv) white cell counts > 12,000/mm^3^ or < 4000/mm^3^ or > 10% bands. However, patients with cirrhosis who commonly are on beta blockers and are unable to mount a white cell response cannot be identified by these traditional criteria. In ACLF, tachycardia and tachypnea are common due to inflammatory response and moderate to severe ascites, while white cell counts may be elevated in alcohol-related ACLF due to the disease per se. Several scores are available to diagnose sepsis and may be beneficial in cirrhosis and ACLF. These include the SEPSIS-3 criteria, which is based on the SOFA score, the CLIF-SOFA score, and the quick SOFA. Among these, the quick SOFA score has shown to be simple yet useful in predicting poorer outcomes in these patients [69–71].

2. **Recommendations: infection and/or sepsis in the natural history of chronic liver disease**
  2.1. Infection leading to organ failure in cirrhosis with or without liver failure
    2.1.1. In individuals with a definite hepatotropic acute insult, bacterial infection is unlikely to precipitate ACLF. (LoE-Moderate, Recommendation- Strong).
    2.1.2. Bacterial infection can be presumed as a precipitating event of organ failure in the absence of other identifiable causes. (LoE-Moderate, Recommendation-Strong).
    2.1.3. Extrahepatic organ failures and infections in patients with ACLF are associated with a poor response to treatment and worse 30-day survival. (LoE-High, Recommendation-Strong).
    2.1.4. There is limited evidence on the sequence of organ failure resulting from infection and further large cohort studies are needed. (LoE-Low, Recommendation-Strong).
    2.1.5. Reactivation of Hepatitis B virus (HBV) infection is a major cause of acute insults precipitating ACLF in HBV-endemic areas (LoE—High, Recommendation—Strong).
    2.1.6. Reactivation of Hepatitis B virus (HBV) infection can be spontaneous, or secondary to nonadherence to drugs, use of immunosuppressive therapies, or HCV direct-acting antivirals. (LoE- High, Recommendation- strong).
    2.1.7. Spontaneous reactivation of hepatitis B may be defined as serum HBV DNA > 20,000 IU/ml or re-appearance of serum HBsAg and/or HBV DNA, or a 2 log rise in HBV DNA from the baseline with an ALT flare over 5 ULN. (LoE- Moderate, Recommendation- weak).
    2.1.8. For HBV reactivation from occult HBV infection, chronic liver disease should be carefully evaluated for ACLF diagnosis (LoE—Low, Recommendation—Strong).
    2.1.9. Acute viral hepatitis due to HAV/HEV virus is a common cause of ACLF and we recommend early screening of suspected patients for these infections. (LoE- High, Recommendation- strong).
    2.1.10. HCV, HDV, Spirochetes, parasites, COVID-19 and fungi may precipitate ACLF. (LoE-Low, Recommendation- weak).
  2.2. Laboratory and clinical diagnosis of infection in cirrhosis with or without decompensation
    2.2.1. Hospitalized patients with ACLF should be monitored (both clinically and through relevant biochemical and microbiological tests) closely for the presence of infections to enable early diagnosis and treatment. (LoE: High; Recommendation: Strong)
    2.2.2. Any deterioration in the clinical or biochemical status should trigger a workup for infection. (LoE: High; Recommendation: Strong)
    2.2.3. Sepsis can be identified using the Sepsis-3 definition. Quick SOFA score can be used for initial sepsis identification (LoE: Moderate; Recommendation: Weak)
    2.2.4. There should be a low threshold to treat infections with broad-spectrum antibiotics early in the disease course based on clinical examination and/or the presence of deterioration, irrespective of biomarkers. (LoE: High; Recommendation: Strong)
    2.2.5. Culture sensitivities should guide antibiotic use, and empirical antibiotics should be de-escalated early in hospitalized patients with cirrhosis if clinically recovering and cultures are sterile. (LoE-High, Recommendation-Strong)
    2.2.6. Acute phase reactants such as CRP and procalcitonin can aid in the diagnosis of infection and assessment of the response to therapy. (LoE: Moderate; Recommendation: Weak)
    2.2.7. 1,3-β-D-glucan and serum galactomannan can aid in the diagnosis of fungal infections and have a good negative predictive value. (LoE: High Recommendation: Weak)

## Organ failure and its implication in patients with liver failure due to an acute insult

### Defining organ dysfunction (OD) and organ failure (OF)

**Fig. 5:**
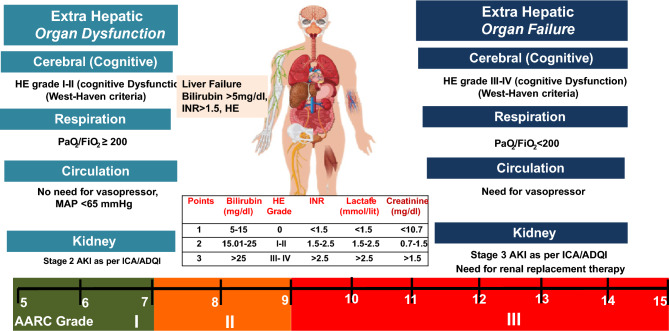
Organ Dysfunction (OD) and Organ Failure (OF) in ACLF

Organ dysfunction and organ failure can impact the outcomes of patients with ACLF. However, according to APASL, organ involvement, except cerebral and hepatic (jaundice and coagulopathy), is not part of the definition of ACLF [1]. Hepatic failure can be defined based on the existing criteria of bilirubin > 5 mg/dl, while there would be no hepatic dysfunction. The definitions of organ failure can be based on the AARC score, although there are no studies to assess the impact of these definitions on ACLF, except for renal and cerebral involvement. Among 1315 (of 3009 patients) patients with HE at admission, 75% had grade I-II HE and 25% had advanced HE [72]. Ammonia levels correlated with the development of HE, and the grade of HE and ammonia levels predicted 30-day mortality [72]. For cerebral involvement, grade I-II HE would be considered as dysfunction, and HE grade III-IV as a failure. The grading is based on West-Haven criteria (Fig. 5).

For coagulation, based on the AARC score an INR of 1.5–2.5 can be considered as coagulation dysfunction and > 2.5 as failure. Lactate is an important component of the AARC score. In fact, the AARC score was the first to include lactate in the assessment of the severity of liver failure. Lactate, an intermediate product of anaerobic metabolism, is a marker of several stress-related metabolic changes derived from multiple sources [73]. On an average, 20 mmol/kg/day of lactate is produced at rest and 60–120 mmol of lactate is cleared every hour [73]. Although the metabolism of lactate in cirrhosis and ACLF is unexplored, several studies have reported that lactate in combination with traditional severity scores such as MELD, MELD Na, and CLIF-C ACLF can accurately predict short-term mortality [74–78]. A higher proportion of patients with elevated lactate levels (> 2 mmol/L) require vasopressor support and mechanical ventilation compared to those with no elevated lactate levels [74]. Whether the lactate levels (as suggested by AARC scoring), in combination with circulatory failure and respiratory failure, can be utilized to define organ dysfunction and failure needs to be assessed in future studies. Till further data, we have included SOFA grading for identifying organ failures for pulmonary and circulatory organs.

### Hepatic vs. extrahepatic OD/OF in decompensated cirrhosis

Organ dysfunction and failure are the main reasons for recurrent admission in decompensated cirrhosis [79]. HE and AKI are common features in decompensated cirrhosis and are known to be associated with increased mortality [80]. Minimal HE is prevalent in 40–60% of patients with Child B and C cirrhosis, while overt HE is 25–30% in Child B and C cirrhosis. More than 60% of patients with cirrhosis develop stage ≥ 2 kidney injury during their lifetime, and AKI is associated with reduced survival in decompensated cirrhosis [81]. Pulmonary complications unique to decompensated cirrhosis include hepatic hydrothorax, hepatopulmonary syndrome, and porto-pulmonary hypertension, which are rarely reported in ACLF [82]. However, infections leading to lung failure are common in ACLF and DC. Rebalanced hemostasis is a characteristic feature of cirrhosis, while in ACLF, coagulopathy is common [83, 84]. The coagulation profile in both ACLF and DC is influenced by the severity of liver disease and sepsis. Organ failure is more rapid in ACLF, while DC has a more gradual progression and decline in organ functioning. Nevertheless, organ failure in DC and ACLF are associated with poor outcomes.

Data from 1343 hospitalized patients with cirrhosis and acute decompensation in 2011 at 29 liver units in eight European countries showed that the 28-day mortality rate among patients who had ACLF when the study began was 33.9%; among those who developed ACLF, it was 29.7%, and among those who did not have ACLF, it was 1.9%; the mortality rate within 28 days after enrolment was 32% in patients with two organ failures and 78.6% in those with three organ failures or more. It was only 14.6% of patients with one organ failure [2]. AKI in patients with ACLF is more likely to progress to CKD compared to cirrhosis [85].

### Renal dysfunction or failure: the course and implication in cirrhosis with predominant liver failure

Patients with ACLF or AD frequently develop AKI. Kidney dysfunction or failure is not considered a part of the ACLF definition by the Asia Pacific Association for the Study of Liver (APASL), but it is part of the severity assessment of ACLF as it is included in the AARC score [86]. On the other hand, the European Association for the Study of Liver Disease defines ACLF based on renal dysfunction [2]. The presence of single kidney failure, i.e. serum creatinine more than or equal to 2 mg/dl, is defined as ACLF grade 2. NACSELD also defines ACLF with the presence of two or more extrahepatic organ failures, wherein kidney failure was defined as a requirement of renal replacement therapy [87]. The challenge with the inclusion of renal dysfunction in the definition of ACLF is twofold; first kidney failure may be a late event as a result of the usage of diuretics or paracentesis used for treating ascites in cirrhosis patients. This may result in a delay in the diagnosis of ACLF. Second, kidney failure may be independent of liver failure and coagulopathy, as often seen in patients with cirrhosis related to alcohol and metabolic factors. Using the APASL definition, AKI is seen in 30–50% of patients with ACLF. The AASLD has recently put forth a revised definition of ACLF, as a syndrome characterized by an acute insult in patients with chronic liver disease, compensated or decompensated cirrhosis characterized by jaundice and coagulopathy followed by the development of at least one extrahepatic organ failure which included the kidneys [6].

**Fig. 6:**
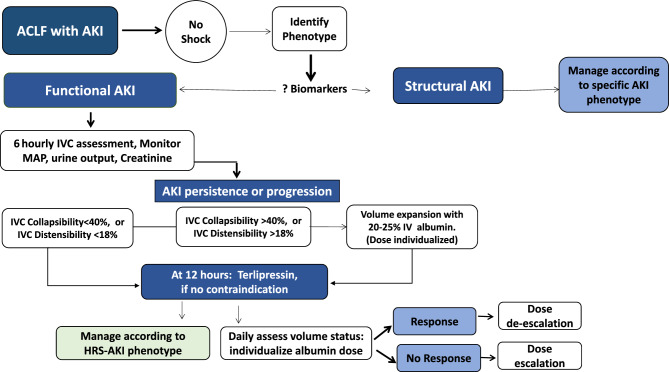
Managing ACLF with AKI

The other emerging concept is the change in the way renal dysfunction should be assessed in patients with ACLF (Fig. 6). Previous definitions used absolute values of serum creatinine for defining kidney dysfunction or failure. The experts unanimously agreed to the incorporation of the revised criteria by the International Club of Ascites and Acute Dialysis and Quality and Initiative Group for defining AKI incorporating urine output. Based on the revised criteria, it was agreed that kidney dysfunction should be considered if the patient has stage 2 AKI, while kidney failure in ACLF should be considered in patients with stage 3 AKI [88]. It was further proposed that a revised definition of hepatorenal syndrome (HRS) should be validated in patients with ACLF. Conceptually, even though HRS is a functional AKI, however, in patients with ACLF, HRS may frequently co-exist with cholemic nephropathy or occur in the presence of tubular dysfunction in the background of severe systemic inflammation [89]. Further, the rapidity of AKI progression and a reduced response to vasoconstrictors favor the diagnosis of HRS in patients with AKI persistence or progression despite appropriate volume expansion within the first 12–24 h of presentation. Oliguria with lesser elevations of serum creatinine may have worse renal and overall outcomes compared to patients who have a preserved urine output, especially in patients with stage 3 AKI in ACLF. Oliguria in patients of ACLF with stage 3 AKI on renal replacement therapy may especially be an indication for a simultaneous liver-kidney liver transplant vs. liver transplant alone in patients with ACLF [90].

### Circulatory and respiratory failures

Organ failures have prognostic implications in decompensated chronic liver disease and ACLF. The number of organ failures correlates with mortality and is widely used to grade severity [2, 91]. The type of organ failure, however, has different implications. While renal failure and high-grade HE are considered to be organ failures of utility and are of predictive value, circulatory and respiratory failures are reflective of the severity of illness and are indicators of futility [1, 92]. Respiratory and circulatory failure in ACLF and decompensated chronic liver disease have different implications based on the circumstances where it develops. While liver failure is central, brain failure and coagulation failure are mechanistically a part of liver failure. Kidney failure has been closely linked with liver failure and has been used in prognostic models like MELD and AARC scores [86, 93]. Respiratory and circulatory failure, on the other hand, are not mechanistically related to liver failure and are reflections of an overall intense systemic inflammatory state and immune paresis.

Incidence of isolated respiratory failure or circulatory failure without other organ failure is less common than renal and HE. In a validation study of CLIF-SOFA score, only 3% and 11% of patients had isolated respiratory and circulatory failure at presentation [94]. Stable or unstable decompensated liver disease developing lung injury (commonly due to pneumonia) escalating into respiratory failure will have a different course and prognosis as compared to those who already have renal failure and HE.

Multivariate analysis was done on the validation cohort of CLIF-SOFA; all organ failures had statistical significance in predicting mortality. The highest odds were observed for renal and liver failure, while the odds for respiratory and circulatory failure were the lowest [94]. However, in the original cohort of the Canonic study, renal failure was more frequently noted in lower grades of ACLF, while respiratory and circulatory failure were observed in higher grades of ACLF [2]. The current organ failure definitions are not precise and can often lead to placing the same patient in different categories and can lead to discordance in prioritizing patients for liver transplantation [95].

Due to the paucity of existing data, more studies to understand the sequence of organ failures and the reversibility of individual organ failures in ACLF and critically ill decompensated liver disease are needed. Nevertheless, respiratory failure (requiring mechanical ventilation) and circulatory failure (requiring high vasopressor support) are indicators of futility in patients with ACLF and decompensated cirrhosis and are less likely to recover [96, 97]. Therefore, these events are considered terminal and irreversible.

3. **Recommendations: organ failure(s) and its implication in patients with liver failure due to an acute insult**
  3.1. Defining the organ dysfunction (OD) and organ failure (OF)
    3.1.1. Overt cognitive dysfunction (cerebral dysfunction) is defined as grade I and II HE and advanced cognitive dysfunction (cerebral failure) as grade III and IV encephalopathy in patients with ACLF and this should be considered as part of liver failure. [LoE- High, Recommendation-Strong]
    3.1.2. Liver failure in ACLF is defined as serum bilirubin ≥ 5 mg/dl and INR ≥ 1.5. [LoE- High, Recommendation-Strong]
    3.1.3. Respiratory dysfunction is defined as patients with ACLF requiring non-invasive oxygen support for hypoxemia with a P/F ratio > 200. [LoE- Low, Recommendation-Weak]
    3.1.4. Respiratory failure is defined as the requirement of mechanical ventilation for hypoxemia and/or P/F ratio < 200 [LoE- Low, Recommendation-Weak].
    3.1.5. Circulatory failure is defined as patients with ACLF requiring vasopressors to maintain MAP > 65 mmHg [LoE- Low, Recommendation-Weak].
    3.1.6. Lactate is an integral component of the AARC score and provides a reliable assessment of the degree of liver failure in ACLF. [LoE- High, Recommendation-Strong].
  3.2. Hepatic vs. extrahepatic OD/OF in a decompensated cirrhosis
    3.2.1. Extrahepatic organ failures impact the outcomes of patients with ACLF and decompensated cirrhosis. Prompt recognition of extra-hepatic organ failure is essential to management and improving outcomes (LoE- High, Recommendation- strong).
    3.2.2. The progression of extrahepatic organ failures is more rapid in ACLF than in decompensated cirrhosis. (LoE- Moderate, Recommendation-Weak)
    3.2.3. Risk prediction models ACLF should include markers of both hepatic and extra-hepatic organ dysfunction and failure in an effort to be generalizable and applicable to all patients with liver disease (LoE- High, Recommendation- Strong).
    3.2.4. Blood ammonia level is elevated in HE in ACLF but its correlation with severity and prognosis needs to be further investigated (LoE- Moderate, Recommendation- strong)
    3.2.5. Need for mechanical ventilation, while the least common among extra-hepatic organ failures, is strongly associated with poor post-transplant survival and should be considered a relative contraindication to liver transplantation. (LoE- Moderate, Recommendation- strong).
  3.3. Renal dysfunction or failure: the course and implication in cirrhosis with predominant liver failure.
    3.3.1. The incidence of AKI in patients with ACLF is higher compared to those with decompensated cirrhosis. [LoE-High, Recommendation-Strong]
    3.3.2. The absolute values of serum creatinine should not be considered for the diagnosis or staging of the severity of renal dysfunction in ACLF patients. (LoE- High, Recommendation- Strong)
    3.3.3. AKI criteria incorporating urine output should be used for the diagnosis and staging of AKI in ACLF patients. (LoE- High, Recommendation- Strong)
    3.3.4. Kidney dysfunction should be defined in patients with stage 2 AKI and kidney failure as stage 3 AKI in ACLF patients as per ICA/ADQI. (LoE- Low, Recommendation- Weak).
  3.4 Circulatory and respiratory failure are terminal events in decompensated cirrhosis (ESLD) rather than the type of organ failure.
    3.4.1. Respiratory and circulatory failures in decompensated cirrhosis are associated with high mortality. (LoE- High, Recommendation- strong)

## Definition of ACLF

### Acute-on-chronic liver failure (ACLF) definition

ACLF is a severe syndrome marked by an acute decline in liver function decline and multi-system organ failure in patients with chronic liver disease. Various definitions have been proposed by major societies across the globe. Each definition recognizes the high mortality rate of ACLF, emphasizing the urgent need for timely, comprehensive intervention to enhance patient outcomes. All definitions stress the rapid onset of ACLF within weeks to months, emphasizing the crucial role of early recognition and intervention in reducing disease severity and enhancing patient survival. Existing ACLF definitions, drawn from varied populations with different etiologies, focus on clinical features linked to high short-term mortality and lack consideration of pathological aspects. To achieve a universal global ACLF definition, it is essential to identify a syndrome with distinctive hepatic pathological features and corresponding pathophysiology across different liver disease etiologies and predispositions.

APASL defines ACLF as acute hepatic insult manifesting as jaundice and coagulopathy complicated within 4 weeks by ascites and or encephalopathy in a patient with previously diagnosed or undiagnosed chronic liver disease with high short-term mortality. Among various definitions, the APASL definition is homogenous with a hepatic insult as an acute event and all patients having liver failure with or without other organ failures. (1) The strength of the APASL definition is that it does not exclude patients who fulfill the definition and, in addition, have extrahepatic organ failure. It does not mandate the presence of extrahepatic OF to define ACLF. Patients with previously decompensated liver disease are not included even with liver failure after acute hepatic insult, as they have a different natural history related to the ESLD stage.

While AARC and WGO definitions highlight acute hepatic insult, EASL/CLIF and NACSELD emphasize the progressive nature of organ failures. COSSH specifically addresses HBV-related ACLF. Recently, AASLD has brought significant clarity to the terminology of ACLF by including liver failure as an essential criterion to diagnose ACLF syndrome [6]. AASLD has proposed the minimum critical components for the definition of ACLF: (1) acute onset with rapid deterioration in clinical condition, (2) the presence of liver failure defined by elevated bilirubin and elevated INR in patients with chronic liver disease with or without cirrhosis, and (3) the presence of at least one extrahepatic (neurologic, circulatory, respiratory, or renal) organ failure.

Besides liver failure, there are differences between societies on whether to include chronic liver disease and decompensated cirrhosis in the ACLF definition. Some definitions include cirrhotic patients, while others recognize ACLF in individuals with chronic liver disease regardless of cirrhosis status, reflecting differences in disease severity and clinical manifestations within the ACLF syndrome. The absence of a consistent global consensus on ACLF definition is evident despite its recognition as a distinct clinical entity. Establishing a definitive ACLF definition requires clear distinctions between acute liver failure (ALF) and “acute decompensation,” understanding its pathophysiology, defining clinical indicators, and employing a validated severity scoring system for optimal therapy. This process demands extensive prospective data collection and validation, acknowledging the varied geographical contributions to ACLF. All these definitions agree that these patients are sick and merit early liver transplantation in the absence of which mortality is very high. There is a need for a unified definition to further progress in the management of these patients. There are several common grounds and overlap between APASL, COSSH, the Chinese Medical Association, and Japanese definitions of ACLF (Table 4).

**Table 4:** Essential features of different ACLF definitions

Essential feature	Liver failure	Extrahepatic organ failure	Both liver and extrahepatic failure	Hepatic insult	Time on onset defined	High mortality	Decompensated cirrhosis
AARC	Yes	No	No	Yes	Yes	Yes	No
EASL-CLIF	No	Yes	No	No	Yes	Yes	Yes
WGO	Yes	Yes	Yes	No	Yes	Yes	Yes
NACSELD	No	Yes	No	No	No	No	Yes
AASLD	Yes	Yes	Yes	No	No	No	Yes
Japanese	Yes	No	No	Yes	Yes	No	No
Chinese	Yes	No	No	Yes	No	No	Yes

*AARC* APASL ACLF Research Consortium, *EASL-CLIF* European Association for Study of Liver-Consortium for liver failure, *WGO* World Gastroenterology Organization, *NACSELD* North American Consortium for End-Stage Liver Disease, *AASLD* American Association for the Study of Liver Disease

Another area of debate is the timeline for defining ACLF, 4 weeks or 12 weeks. As per APASL, ACLF is a syndrome of hepatic decompensation, where the insult is primarily hepatic, leading to liver failure within 4 weeks, with jaundice and coagulopathy preceding the development of ascites [1]. This timeframe of 4 weeks was extrapolated from the definition of ALF. The time range was set at 4 weeks since the fundamental idea behind ACLF was to identify individuals with chronic liver disease or cirrhosis presenting with acute liver failure who are at risk of higher mortality [98]. While the APASL identifies ACLF and acute decompensation as two separate entities with different prognoses, EASL puts ACLF and acute decompensation in the same spectrum, with ACLF being the severest form of acute decompensation associated with organ failure [2]. Kim et al. analyzed data from 1861 patients with CLD and acute deterioration, among which the proportions of ACLF based on the AARC and CLIF-C definitions were 9.5% and 18.6%, respectively. The 28-day mortality (35.4% vs. 26.4%) and 90-day mortality (54.5% vs. 42.4%) rates were significantly higher in those fulfilling the CLIF-C criteria than the AARC criteria [99]. Rajoo et al. studied the outcome of patients with cirrhosis and acute decompensation of liver disease. The 90-day mortality was significantly higher for patients fulfilling both APASL and EASL criteria (71.4%), compared to either APASL alone (35.4%) or EASL alone (53.3%). Interestingly, events leading to acute deterioration were also different between the groups. The patients fulfilling EASL criteria were more likely to have had a bacterial infection as a trigger compared to APASL criteria, in which hepatitis B flare was the most common trigger [100]. Mahmud et al. analyzed the follow-up data of patients with compensated cirrhosis for the first episode of ACLF. The incidence rate of APASL ACLF was 5.7 (95% CI 5.4–6.0), and EASL ACLF was 20.1 (95% CI 19.5–20.6)/1000 person-years. The 28-day mortality (41.9% vs. 37.6%) and 90-day mortality (56.1% vs. 50.4%) were comparable between APASL and EASL ACLF [101]. Thus, the two ACLF definitions identify patients with different characteristics with different prognoses.

The AARC database was analyzed to assess whether a 2-week cutoff, as opposed to 4 weeks, would be a more accurate measure of mortality among patients with underlying cirrhosis who experienced jaundice followed by ascites. The analysis showed that patients developing ascites within 2 weeks of jaundice had a higher mortality compared to those who developed ascites within 2 to 4 weeks [2]. Thus, a shorter interval from jaundice to decompensation indicates a poorer prognosis. In the absence of similar data comparing jaundice to decompensation intervals of 4 weeks, 8 weeks, and 12 weeks, it may be assumed that the longer the interval from jaundice to decompensation, the better the short-term prognosis.

### Unifying pre-existing ACLF definitions- The Kyoto Consensus

The primary aim of the definition of ACLF should be a simple and clear recognition of the syndrome, highlighting potential reversibility with the possibility of timely intervention beyond emphasizing mortality rates. Identifying distinctive hepatic pathological features and corresponding pathophysiology for ACLF syndrome is the premier pathway to reach a universal global ACLF definition from different etiologies and predisposition to liver disease patients.

*Unifying all criteria:* APASL identifies three organ failures in its definition which are primarily related to severe liver dysfunction; these include liver, coagulation, and HE. Using this definition, APASL identifies patients before extrahepatic organ failure ensues. The same premise and basis have been used by the AASLD guidance on ACLF. Other definitions do not require liver failure but do require failure of one or more extrahepatic organ in a cirrhosis patient. Unifying the definitions needs mutual understanding and the Kyoto consensus with all global experts has set the ball rolling.

We had recently proposed ***ACLF be redefined to be inclusive of all global definitions*** as “ACLF is a distinct reversible syndrome characterized by rapid onset liver failure (bilirubin > 5 mg/dl and INR ≥ 1.5) followed by ascites and/or encephalopathy within 28 days associated with or without kidney dysfunction (serum creatinine > 1.5 mg/dl) in a patient with chronic liver disease or cirrhosis, and is associated with high mortality till 28 days”. Needless to say, we would need prospective data to support the utility of this definition over the existing definitions.

**Fig. 7:**
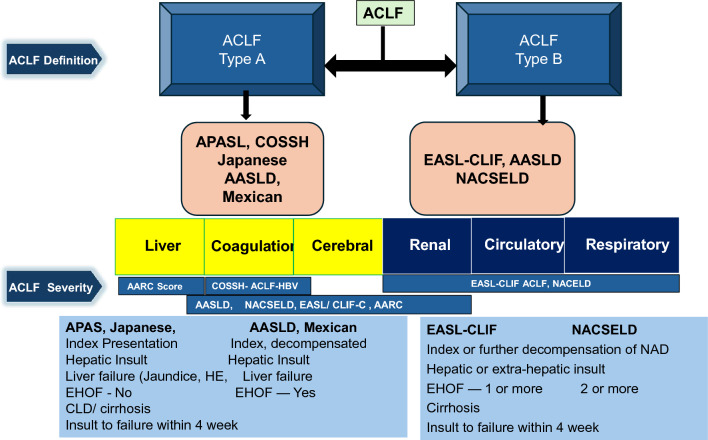
Proposal for Unified ACLF Definition (Kyoto Consensus)

Furthermore, a common path can be found to ***simplify classifications***. [102] APASL ACLF can be labelled as type A -ACLF and graded based on AARC score as grade AI, AII, AIII. EASL-CLIF ACLF can be labelled as type B and sub-grouped based on organ failures as B1, B2, and B3 (Fig. 7). *Type A-ACLF* will not include patients with previous decompensation or patients with extrahepatic insults and can be reversible and considers infection consequently more often than as a precipitant. Conversely, *Type B-ACLF* could include patients with previous decompensation and organ failure caused by both hepatic and extrahepatic insults, including infection, and is associated with elevated mortality. Type A -ACLF includes the Japanese definition of ACLF, and type B would be similar to the COSSH definition (liver failure) of ACLF. NACSELD labelled ACLF can be reclassified as B2 and B3 based on the number of organ failures. This will preserve the prior research findings documented in the literature, facilitating the efforts of scientists already engaged in this field.

4. **Recommendations: ACLF-Definition**
  4.1. ACLF definitions: similarities, differences, and steps for unifying
    4.1.1. The existing definitions of ACLF, derived from clinical evidence obtained from populations with different etiologies and predispositions, are based on clinical characteristics defining high short-term (28-/90-day) mortality without incorporating pathological features. (LoE -Moderate, Recommendations-Strong)
    4.1.2. It is proposed to classify ACLF for global acceptability into type A and type B. (LoE: Low; Recommendation: Strong).
    4.1.3. Type A ACLF includes the APASL and Japanese definitions, while type B includes the AASLD, EASL-CLIF, NACSELD, and COSSH definitions. (LoE: Low; Recommendation: Strong)
    4.1.4. ***Type A ACLF*** includes patients with first-time acute insult resulting in features of liver failure in a patient with underlying chronic liver disease or cirrhosis. It does not include patients with previous decompensation or extrahepatic insults. It can be reversible and considers infection as a consequence more often than a precipitant of ACLF and is associated with high (30–40%) 28-day mortality. (LoE: High; Recommendation: Strong)
    4.1.5. ***Type B ACLF*** can include patients with Type A as well as patients with previous decompensation and organ failure caused by both hepatic and extrahepatic insults, including infection, and is associated with high (40–50%) 28-day mortality. (LoE: High; Recommendation: Strong)
    4.1.6. For defining ACLF, a period of presentation within four weeks should be considered. (Evidence- High, Recommendation- Strong).
    4.1.7. To reach a universal global ACLF definition, a syndrome characterized by distinctive hepatic injury and pathological features and corresponding clinical phenotypes should be identified in different etiologies and with various severity of chronic liver disease/cirrhosis. (LoE-Low, Recommendations-Strong)

## Defining common acute insults in ACLF

### Alcohol-associated hepatitis in ACLF and jaundice in decompensated cirrhosis

Alcohol-associated hepatitis (AAH) in ACLF and Jaundice in decompensated cirrhosis are different with respect to their pathophysiology, prognosis, and management. In AAH within the context of ACLF, systemic inflammation emerges as a pivotal mechanism, precipitating tissue hypoperfusion, immune-mediated tissue injury, and mitochondrial dysfunction; however, there is a timeframe of occurring within a month. Moreover, immune dysregulation heightens susceptibility to infections, exacerbating the condition's severity and clinical course [103].

Jaundice in decompensated cirrhosis signifies the culmination of advanced liver disease, characterized by chronic inflammation and fibrosis, resulting in structural and functional liver impairment. There is no timeframe in time for decompensation. Various factors, such as worsening portal hypertension, hepatocellular dysfunction, impaired bile flow, infections, drugs, variceal bleeding, and the development of HCC, can contribute to its onset and progression [104].

The prognosis of ACLF in AAH can vary widely depending on the severity of the condition and the presence of complicating factors such as liver failure, renal failure, or infections. In general, severe cases of AH have a high short-term mortality rate, with estimates ranging from 20 to 50% within 30 days of diagnosis. Numerous scoring systems are present to characterize the severity and prognosis of AAH, including CTP, MELD, MDF, AARC, and CLIF-C ACLF scores. The prognosis of patients who develop jaundice in decompensated cirrhosis is poor. A landmark study by D'Amico et al. found that the development of jaundice was a strong predictor of mortality in patients with decompensated cirrhosis, with a median survival of only 3 months in those who developed jaundice compared to 12 months in those who did not [105].

### Drug-induced liver injury (drugs, CAM and HDS)

Drug-induced liver injury (DILI) leading to ACLF or drug-induced ACLF (DI-ACLF), is an increasingly recognized clinical entity globally. DI-ACLF is also increasingly reported in Asian countries. The majority of DI-ACLF cases are due to complementary and alternative medicines (CAM) and, to a lesser extent, by antituberculosis therapy (ATT), through either an idiosyncratic mechanism or a dose-dependent manner [106]. DI-ACLF inherits twin challenges of both DILI and ACLF, including a lack of a definitive diagnostic test for DILI and differing diagnostic criteria for ACLF. A simple definition of DI-ACLF is at least 2–threefold elevation in AST/ALT with or without twofold elevation in bilirubin from baseline levels, and new or acute hepatic decompensations with or without extrahepatic organ failures, and its association with high short-term risk of mortality. [107] Patients with cirrhosis, on the other hand, may not exhibit a significant increase in AST/ALT; instead, increased bilirubin combined with elevated INR or, in some instances, worsening of the MELD score may be features of DI-ACLF.

The criteria to diagnose DI-ACLF include (1) an acute hepatic insult from DILI as a precipitating event, (2) a strong temporal relationship between drug exposure and development of ACLF, (3) laboratory, serological, virological and imaging studies excluding competing etiologies, and (4) the presence of underlying liver disease [107].

**Fig. 8:**
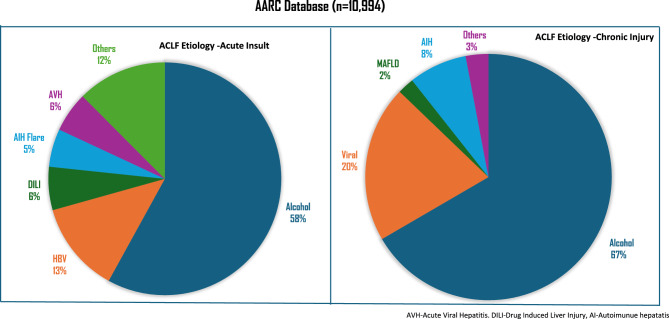
Etiology of ACLF in Asia–Pacific

In the DILI network registry study from the United States, 16% of 89 patients with pre-existing liver disease died compared to 5% of 810 patients with no underlying liver disease [108]. This finding was supported by results from the Spanish DILI Registry where 6.3% of total 843 patients with underlying CLD had a fourfold increased risk of liver-related death due to DILI [109]. In the large, prospective study of 3132 ACLF patients from the multinational APASL AARC database, 329 (10.5%) accounted for DI-ACLF [107]. ATT and CAMs constituted 27.3% and 71.7% of cases, respectively, with methotrexate and anti-epileptic drugs accounting for the remaining 1%. The most common underlying liver disease was alcohol (28.6%), followed by cryptogenic (25.5%) liver disease and NASH (16.7%). The overall 90-day mortality was greater in DI-ACLF (46.5%) than in non–DI–ACLF (38.8%) (*p* = 0.007) [107]. (Fig. 8).

There are increasing reports of CAM and body-building supplements-induced liver injury leading to ACLF, particularly in Asian countries [110, 111]. Identifying the precise ingredient in a multiherb preparation is challenging. However, in recent years, *Polygonum multiflorum* in China [112] and other Asian countries, as well as *Tinospora cordifolia* (Giloy) in India [113, 114] have emerged as the top two medications causing CAM-induced DILI and DI-ACLF. Tinospora cordifolia-induced DILI is associated with a high frequency of autoimmune markers (~ 50%) and mortality (~ 20%) [113], while *Polygonum multiforme* is linked to HLA-B*35:01. There are also emerging reports of other traditional medicinal substances, such as Ashwagandha and curcumin, producing DILI and DI-ACLF [115, 116]. The use of immune checkpoint inhibitors is on the rise. These drugs are known to precipitate liver failure either due to immunological activation or by viral reactivation. It is unknown whether these drugs can precipitate ACLF in patients with cirrhosis, which merits further research.

### Autoimmune liver disease

With a heterogenous presentation, autoimmune hepatitis (AIH) presenting as ACLF (AIH – ACLF) or acute severe AIH with encephalopathy (AIH – ALF) has been more frequently reported over the last few years both in adult and pediatric population from many parts of the world and constitute about 20% of cases of AIH. These patients present with jaundice, HE, and coagulopathy, with or without ascites. In the AARC database, it was noted that 43% did not have detectable autoantibodies, and hence a trans jugular liver biopsy was needed for diagnosis [117]. There are distinct histological features between AIH–ACLF and AIH–ALF. In a single-center study, Stravitz et al. identified histological features like lymphoid aggregates, perivenulitis, and massive hepatitis necrosis as suggestive of AIH – ALF, while advanced fibrosis (F3/F4), ductular reactions, and large areas of parenchymal collapse with lymphoplasmacytic inflammation are more common in AIH – ACLF [118, 119]. Interface hepatitis and lymphoplasmacytic infiltrates are seen in 80% of patients with AIH – ACLF from the AARC database [117]. In another single-center Japanese study, acute hepatitis with centrifugal, submassive, and massive necrosis was found on histology in 86% of cases with fulminant AIH [120]. The diagnosis of AIH is based on the simplified scoring system, and treatment with corticosteroids has an excellent response. [119]

### Vascular liver diseases

The disease burden, clinical picture, prognosis, and treatment strategies of Budd Chiari Syndrome (BCS) or Portal Vein Thrombosis (PVT) presenting as ACLF are largely unknown. A single-center retrospective study reported the incidence of ACLF among BCS patients to be 5% (28/553) [121]. Approximately 61% had no acute precipitant identified while stent thrombosis, acute Hepatic Vein thrombosis, acute viral hepatitis, and antituberculosis drug with hepatitis B virus reactivation were noted in 18%, 10.7%, 7.1%, and 3.6% respectively. The authors reported that endovascular intervention was associated with improved survival while infection and HE were associated with poor survival [121]. The 1- and 3-year transplant-free survival rates in the group that underwent intervention were 86.5% and 69%, respectively. The thrombophilic disorders in those with ACLF have not been evaluated but are unlikely to be different from those without ACLF [122]. The reduction of hepatic blood flow due to acute portal vein thrombosis (PVT) may lead to ischemic liver injury [123]. The diagnosis of acute or chronic BCS in the study by Langlet et al was based on the presence of both acute and chronic features, clinically and/or radiologically [124]. The entity of ACLF was not described at that time and it is unclear if any of these patients would have fulfilled the criteria of ACLF. However, it was reported that these patients had worse outcomes as compared to other patients with Budd-Chiari syndrome. The evaluation of thrombophilic disorders in patients with PVT or Hepatic Vin Outflow Obstruction (HVOTO) and ACLF should be similar to that of patients without ACLF. There is currently no evidence to suggest that non-cirrhotic portal fibrosis or Extra-hepatic Portal Vein Obstruction (EHPVO) may present as ACLF. The APASL guidelines on BCS have suggested classifying BCS-ACLF into three types, as shown in Table 5.

**Table 5:** Types of Budd-Chiari Syndrome presenting as acute-on-chronic liver failure

Type	Pathology	Management
A	Acute hepatic vein thrombosis or stent block precipitates ACLF in a BCS	Urgent recanalization as per anatomy Options: 1. Thrombectomy or thrombolysis with stenting 2. HV stenting 3. TIPS
B		B1: BCS treated successfully previously: Treat like any other ACLF B2: BCS untreated previously 1. Treatment of BCS after recovery from acute insult 2. Liver transplant
C		1. Thrombectomy or thrombolysis with stenting 2. HV stenting 3. TIPS 4. Liver transplant

*HV* Hepaic Vein, *TIPS* Transjugular Intrahepatic Portosystemic Shunt

### Variceal bleed, post-TACE or post-hepatectomy liver failure

AVB has been included as one of the events to define acute decompensation in the natural history of cirrhosis [23]. AVB in a patient with ACLF has been reported to be associated with increased mortality; however, AVB per se leading to ACLF is less frequent. If an individual develops liver failure (jaundice and coagulopathy) due to variceal bleeding, then an acute event is considered AVB. The liver failure in such patients is likely to be due to hepatic ischemia and subsequent bacterial infections [125, 126]. *N*-acetylcysteine has been reported to prevent ischemic hepatitis-related liver failure in patients with cirrhosis who present with severe AVB [127]. It is unknown whether pre-emptive TIPS in patients with cirrhosis and AVB can prevent the development of ACLF in a patient with compensated cirrhosis. Although such preventive measures are necessary to prevent ACLF, a patient with prior decompensation (ascites or HE) who presents with AVB-related ACLF cannot be included in the APASL ACLF criteria. Based on the data, it was unanimously agreed that AVB is not an acute hepatic insult in patients where it produces jaundice and coagulopathy, fulfilling the criteria of ACLF. Few cases of post-TIPS liver failure have been reported [128]. Liver failure is common in those with higher baseline CTP score and prior history of HE [129].

### Bacterial infections

Cirrhosis is associated with an increased incidence of bacterial and fungal infections resulting in hospitalization and complicates hospital admissions in up to 40% of cases [130]. In patients with cirrhosis, disruption of the immune system has been shown to be a common pathway and susceptibility to infection is one of the most common complications. Infection can lead to worsening liver synthetic function and precipitate complications including variceal bleeding, HE, AKI, and multi-organ failure, contributing to high mortality [131]. 37% of patients with decompensated cirrhosis will be re-admitted to the hospital within 30 days [79] and the prevalence of multidrug anti-microbial resistance (AMR) continues to rise with 34% of cultures growing multidrug-resistant organisms in a global study which caused higher mortality than non-AMR infections [132].

As per Western data starting from the seminal CANONIC Study, renal failure is the most common extra-hepatic organ failure [133]. In the multicentre, prospective, observational PREDICT study which included 1273 non-electively hospitalized patients with acute decompensation (No ACLF = 1071; ACLF = 202), almost all (96%) showed proven bacterial infection either alone or in combination with other events. 90-day mortality was paralleled with increasing levels of surrogates for systemic inflammation. Adequate first-line antibiotic treatment of proven bacterial infections was associated with a lower ACLF development rate and lower 90-day mortality [134].

It is evident that in these cohorts of patients, infection led to the development of organ failure and high mortality, though liver failure in a large proportion of patients was not there (only 14% in the canonic study). These patient scenarios are quite common. However, whether the organ failures form the natural course of a cirrhosis patient who develops an infection, or are a unique syndrome where liver failure leads to the development of infections and organ failure remains debatable. The recent AASLD guidelines include the presence of hepatic failure, as an integral part of the ACLF syndrome. As long as there is a concurrent presence of hepatic failure, the presence of infection, evident or not can be considered as part of the ACLF.

Antibiotics have been shown to reduce infections and mortality in acute variceal hemorrhage [135] and reduce infections, but not mortality, in severe alcoholic hepatitis and acute-on-chronicliver failure [136].

In the hospitalized acutely decompensated cirrhotic patients in the ATTIRE study, there was no overall reduction in hospital-acquired infection in patients prescribed antibiotics empirically at baseline in the absence of a clinical diagnosis of infection [137]. The median day of overall hospital-acquired infection diagnosis was day 6 with the most common infections being respiratory (39%), spontaneous bacterial infection (10%), urinary tract infection (8%), and skin/soft tissue (8%). Empirical antibiotic use in those admitted with decompensated cirrhosis overall had no beneficial impact on preventing hospital-acquired infection, nor renal dysfunction and no impact on survival. These data support a policy of prompt de-escalation or discontinuation of empirical antibiotics guided by culture sensitivities at 24–48 h after commencement if no infection and an improving patient [137]. However, infection as a precipitant for the development of ACLF, in the absence of primary hepatic failure remains a debatable point and many investigators from the US, do expect more data. The recent AASLD guidelines on ACLF, have also included liver failure as part of the ACLF definition and not as an extrahepatic organ failure.

5. **Recommendations: acute insult in ACLF**
  5.1. Alcohol-associated Hepatitis in ACLF and Jaundice in Decompensated Cirrhosis
    5.1.1. AAH, alcohol-associated ACLF, and jaundice in decompensated cirrhosis are distinct clinical entities based on pathophysiology, management, and prognosis. (LoE: Moderate; Recommendation: Strong)
    5.1.2. AAH and Alcohol associated ACLF are defined by significant alcohol intake within the last 8 weeks (LoE: Moderate; Recommendation: Strong)
    5.1.3. Development of ACLF in AAH is associated with higher mortality as compared to AAH and decompensated cirrhosis alone. (LoE: Moderate; Recommendation: Strong)
  5.2. Drug-induced liver injury (drugs, CAM and HDS)
    5.2.1 Diagnosis of DI-ACLF is challenging as liver disease-related fluctuations in the liver function tests may be part of the natural history of the disease and may confound the diagnosis. (LoE- High, Recommendation-Strong).
    5.2.2 Complementary and alternative medications (CAM), and antituberculosis drugs are common causes of DI-ACLF. Diagnosis is based on history and RUCAM scoring (exclusion of other causes). (LoE- High, Recommendation- Weak).
    5.2.3 The prognosis of severe DI-ACLF is poor in the absence of liver transplantation. (LoE- High, Recommendation- Strong).
    5.2.4 The risk of liver injury with anti-tuberculosis drugs is proportional to the number of hepatotoxic drugs. (LoE- Moderate, Recommendation- Strong).
    5.2.5 In patients with underlying chronic liver disease or cirrhosis, baseline liver biochemistry tests should be carried out along with HBsAg and anti-HCV screening before initiating antituberculosis drugs. Subsequent monitoring should include liver biochemistry and INR every one to two weeks. (LoE- Moderate, Recommendation- weak).
  5.3. Autoimmune liver disease
    5.3.1. AIH is not an uncommon cause of ACLF (LoE-Moderate; Recommendation-Strong)
    5.3.2. Corticosteroids are the drug of choice in patients with AIH and AIH ACLF (grade I and II), and have no contraindications. (LoE-High; Recommendation-Strong)
  5.4. Vascular liver diseases (PVT, BCS)
    5.4.1. Acute hepatic vein thrombosis or occlusion of TIPS or HV stent can precipitate ACLF. (LoE- Low, Recommendation- Strong).
    5.4.2. Acute portal vein thrombosis can precipitate ACLF in a patient with cirrhosis. (LoE—Low, Recommendation- Weak).
    5.4.3. Anticoagulation can be considered in patients with ACLF due to vascular etiology. (LoE—Low, Recommendation- Weak).
    5.4.4. Angioplasty, DIPS/TIPS can be performed in select patients with BCS-ACLF in the absence of LT. (LoE—Low, Recommendation- Weak).
  5.5. Variceal bleed, post-TACE or post-hepatectomy liver failure
    5.5.1. Variceal bleeding can lead to ACLF in a proportion of patients with compensated chronic liver disease. (LoE—Moderate, Recommendation- strong).
    5.5.2. Variceal bleeding in patients with ACLF is associated with high mortality. (LoE- Moderate, Recommendation- strong).
    5.5.3. TIPS is associated with a low risk of ACLF. (LoE-Moderate, Recommendation- Strong).
    5.5.4. Post-TACE ACLF is common in patients with advanced liver disease, cirrhosis. (LoE- Moderate, Recommendation- strong).
  5.6. Bacterial infections
    5.6.1 Patients with cirrhosis are highly susceptible to developing bacterial and fungal infections associated with a high likelihood of hospitalization and/or development of organ failure(s), the commonest being circulatory and renal. (LoE- Moderate, Recommendation- strong)
    5.6.2 As per EASL, survival in infection-related acute-on-chronic liver failure (ACLF) is determined by the development and number of extra-hepatic organ failures. (LoE-High, Recommendation- strong)
    5.6.3 Antibiotics should be discontinued in hospitalized patients with cirrhosis if no infection is identified (guided by culture sensitivities at 24–48 h after commencement) and the patient is improving. (LoE-High, Recommendation- strong)
    5.6.4 Patients hospitalized with cirrhosis have a high incidence of antimicrobial resistance (AMR) with 34% of cultures growing multidrug-resistant organisms in a global study which caused higher mortality than non-AMR infections. (LoE-High, Recommendation- strong)

## Defining the underlying chronic liver disease in ACLF

### CLD with or without cirrhosis

Patients with ACLF have underlying chronic liver disease (CLD) or cirrhosis. CLD is traditionally defined as the progressive deterioration of liver functions for more than six months, which includes the dysfunction in the clotting factors and other protein synthesis, impaired detoxification of harmful products of metabolism, and bile synthesis and excretion [17] (Fig. 1). CLD is a continuous process of inflammation, destruction, and regeneration of liver parenchyma, which leads to fibrosis and cirrhosis [17]. Compensated advanced chronic liver disease (cACLD), as defined by BAVENO, includes a spectrum of diseases ranging from advanced fibrosis and cirrhosis in asymptomatic patients who are at risk of developing clinically significant portal hypertension (CSPH) [138]. cACLD is defined based on the transient elastography of > 15 kPa while such patients developing ACLF are less known. Assessment of the degree of fibrosis in patients with previously unknown status during the episode of ACLF is challenging. A single-center study including patients with alcohol-related ACLF reported the mean LSM among 14 patients who underwent transient elastography at baseline was 71.01 ± 5.52 kPa. On follow-up after the reversal of ACLF, 34 patients underwent transient elastography, and the mean LSM was 46.41 ± 17.51 kPa. None of the patients had LSM less than 10 kPa [139]. Detailed history and general physical examination may help identify stigmata of cirrhosis/cACLD. Imaging modalities such as USG abdomen and CT scan may reveal the presence of cirrhosis. However, in patients with high-grade fibrosis in the setting of jaundice, ascites, and liver failure, non-invasive modalities like transient elastography and radio imaging may not be helpful in the assessment of the degree of fibrosis. Diagnosis of the etiology of chronic liver disease is important and has a large impact on the management and outcome of these patients. Viral etiologies may be diagnosed with the help of serologies and PCR tests. History of alcohol, obesity, diabetes, and dyslipidemia give clues to the presence of ALD and MASLD. Underlying AIH and Wilson are important differentials, especially when there are high transaminase, jaundice, hemolysis and absence of alcohol, viral, and metabolic risk factors. In these cases, liver biopsy is essential and due to the presence of coagulopathy and thrombocytopenia trans jugular liver biopsy is only feasible. Additionally, endoscopic ultrasound-guided liver biopsy is the upcoming modality in patients with liver diseases; however, at present, due to a lack of sufficient evidence, it may not be recommended in patients with ACLF.

### ‘Index’ presentation or ‘ACLF Again’

As discussed above reversal of ACLF is not uncommon. Once reversal is achieved, the liver stiffness may remain high indicating underlying cirrhosis. These patients can develop ACLF again if they relapse (alcohol) or stop NUCs or after withdrawal of immunosuppressants in AIH. Patients who develop ACLF again have higher mortality at 28 days than those who present as an index case [140].

### Impact of comorbidities and obesity

There is an exponential increase in the burden of MAFLD/MASLD globally [141]. Approximately 15–30% of patients with ACLF have diabetes mellitus, obesity, hypertension, and dyslipidemia, and these patients have severe liver disease. Mortality in such patients is higher compared to those without features of metabolic syndrome [142–145] (Table 6). MELD-Na and AARC scores at admission were higher in patients with MAFLD, i.e., 32 ± 6 and 10.4 ± 1.9, respectively. Furthermore, obesity (overweight) and diabetes mellitus are associated with higher mortality to 90 days in patients with ACLF [142, 143]. The presence of diabetes and precipitant to MELD-Na and AARC scores, the novel MAFLD-MELD-Na score (+ 12 for diabetes, + 12 for non-viral precipitant) and MAFLD-AARC score (+ 5 for each) outperformed the standard scores in predicting mortality in patients with ACLF.

**Table 6:** Impact of the presence of comorbidities on the course of ACLF

Author, Year (Ref.)	Type of study	No. of patients	Age, yrs	Co-morbidities	MELD score	Morbidity	Mortality
Duseja et al. 2021 [142]	Multi-centre, prospective cohort (2013)	1216	42.5 (± 9.4)	Obesity:154 (15%) DM: 142 (14%) HTN: 66 (7%) Dyslipidaemia:141(15%)	29.7(± 7)		90-day: 43%
Lai et al. 2021 [144]	Single centre Retrospective cohort 2013- 2020	38	56.3 (± 14.2)	DM:100% BMI: 24.99 (± 3.32)	20.89 (± 5)	Non-SBP infection: 44.74%	65.8%
Sundaram et al. 2018 [145]	Single centre Retrospective cohort 2015- 2016	64	44 (13–65)	NAFLD: 35.37%		Renal: 37 (57.8%) Liver: 45 (70.3%) Brain: 24 (37.5%) Circulatory:14 (21.9%) Coagulation:22 (34.4%) Mechanical ventilation: 25%	28-day: 43.75%
Selva Rajoo et al. 2017 [100]	Retrospective cohort, 2004–2014	14	53 (48–57)	DM:50%	27 (23–31)		During admission: 28.6% 90-day mortality: 35.7%

### Changing trends for the etiology of acute insult and chronic injury

The epidemiology of acute insult has changed significantly in the past 5 years. In the AARC, COSSH, and CLIF data, alcohol is now the most frequent etiology for acute insult as well as for the underlying chronic liver disease (41%), and HBV remains the second common etiology (27%) in the Asian regions (Fig. 8) [1, 5, 146, 147] DILI, autoimmune, and nonalcoholic steatohepatitis etiologies have shown an increasing trend; however, HAV/HEV/HCV showed a decreasing trend [101]. Targeted/immune therapies-induced ACLF is emerging [148]. HBV infection-induced ACLF, as well as HAV/HEV/HCV-induced ACLF, is now showing a decreasing trend over time, whereas alcohol and herbs, drugs, and supplements (HDS)-induced ACLF show an increasing trend. ACLF due to vascular diseases is being reported and studies on natural history and outcomes are awaited [122]. The unknown causes for acute insult and chronic injury constitute only 10–15% cases of ACLF in the East in contrast to the West, where these are seen in about 40% of ACLF patients.

6. **Recommendations: defining the underlying chronic liver disease in ACLF**
  6.1. CLD with or without cirrhosis
    6.1.1. Cirrhosis and non-cirrhotic chronic liver diseases qualify as chronic liver diseases. (LoE- High, Recommendation- strong).
    6.1.2. Chronic hepatitis and/or significant fibrosis without cirrhosis should be taken as a chronic liver disease if such a patient presents as ACLF. (LoE- High, Recommendation- Weak).
    6.1.3. MAFLD-related chronic hepatic injury; MASH, if associated with significant fibrosis, should be taken as a chronic liver disease in ACLF. (LoE- High, Recommendation- strong).
    6.1.4. Diagnosis of chronic liver disease and cirrhosis in the setting of ACLF is made by history, physical examination, laboratory, endoscopic, or radiological investigations. (LoE- High, Recommendation- strong).
    6.1.5. A liver biopsy may be helpful when the presence of underlying chronic liver disease and/or the cause of chronic liver disease and/or the acute insult is not clear. (LoE- High, Recommendation- weak).
    6.1.6. Patients with known previous decompensation with jaundice, HE, and ascites should be excluded. (LoE- High, Recommendation- weak).
  6.2. Index presentation or ‘ACLF Again’
    6.2.1. Patients who achieve reversal of ACLF can develop ‘ACLF again’. (LoE- Moderate, Recommendation- Strong).
    6.2.2. All subsequent episodes of ACLF should be evaluated and managed like that of patients with “index” ACLF. (LoE-Moderate, Recommendation- Strong).
  6.3. Impact of comorbidities and obesity
    6.3.1. Patients with ACLF and concomitant comorbidities present with advanced liver disease. (LoE- Moderate, Recommendation- Strong).
    6.3.2. The presence of diabetes mellitus or obesity increases mortality in patients with ACLF. (LoE- Moderate, Recommendation- strong).
    6.3.3. The presence of obesity in ACLF patients necessitates proper assessment and management of airway and ventilation as well as nutritional therapy. (LoE-Low, Recommendation- strong).
    6.3.4. The presence of diabetes mellitus or obesity in ACLF patients warrants additional pre-LT cardiac assessment and screening for malignancy. (LoE- Moderate, Recommendation- strong).
  6.4. Changing trends for the etiology of acute insult and chronic injury
    6.4.1. Alcohol is now the commonest etiology for acute hepatic insult as well as for the underlying chronic liver disease in the Asian continent. (LoE- High, Recommendation- strong).
    6.4.2. DILI, autoimmune, and nonalcoholic steatohepatitis etiologies have shown an increasing trend. (LoE- Moderate, Recommendation- moderate).
    6.4.3. HBV infection–reactivation of hepatitis B-induced ACLF as well as acute HAV/HEV/HCV-induced ACLF show a decreasing trend over time in certain regions, whereas alcohol and herbs, drugs, and supplements (HDS)-induced ACLF show an increasing trend. (LoE- High, Recommendation- strong).
    6.4.4. The unknown causes for acute insult and chronic injury constitute only 10–15% of cases of ACLF in the East in contrast to the West, where these are seen in about 40% of ACLF patients. (LoE- High, Recommendation- strong).

## ACLF and acute decompensation (AD) are distinct: differentiating AD and ACLF

### Natural history and outcome of ACLF

ACLF is a syndrome due to acute deterioration of underlying chronic liver diseases resulting from acute precipitating events, which may lead to multi-organ failure with high short-term mortality. The common precipitants include alcohol, viruses, and drugs.

In the West as well as the East, AAH constitutes the major cause of ACLF [149]. Infections as a precipitant are reported more often in ACLF from the West [3] while HBV reactivation remains a dominant cause of ACLF in some Asia–Pacific countries like China and Bangladesh. Both alcohol and HBV are common in many countries like India [86, 150, 151]. The overall mortality of ACLF patients is quite high, with 40–50% of the patients dying by 28 days and 50–60% by 90 days [86, 152, 153]. Liver transplantation is the definitive treatment.

The outcomes of ACLF are dependent on the stage of the ACLF and associated complications (like acute variceal bleeding) and their dynamic changes during follow-up. There are various staging systems for ACLF (discussed in a later section). In general, staging is dependent on the level of jaundice, INR derangement, cerebral involvement (HE), kidney involvement, circulatory system involvement, and lung involvement. In patients diagnosed using APASL ACLF criteria, the AARC score is a good etiology nonspecific staging system; and Tongji prognostic predictor model score, Chinese Group on the Study of Severe Hepatitis B–ACLF score (COSSH–ACLF score), and even the MELD score are good HBV-ACLF specific scores [5, 86, 150, 154]. Higher stages of ACLF (i.e., more number of extrahepatic organs getting involved and start failing) are associated with higher mortality. These staging systems can also be used sequentially to dynamically assess the responses to interventions. The development of acute variceal bleeding also confers poor outcomes in patients with ACLF with a high mortality [155]. In patients with alcohol-associated cirrhosis/AAH-bacterial infections as a cause of ACLF and extrahepatic organ failures are frequently present at initial presentation as ACLF, whereas, among patients with HBV-related ACLF, extra-hepatic organ failures are a late phenomenon [5]. Patients with hepatitis C or NAFLD/MAFLD as underlying chronic liver disease and developing ACLF have higher short-term mortality compared with patients with HBV-related ACLF and alcohol-related ACLF [101].

### Natural history and outcomes of acute decompensation

Acute decompensation (AD) of cirrhosis is a common cause of admission in patients with cirrhosis. It is traditionally defined as any of the following events: jaundice, ascites, HE (hepatic decompensating events), gastrointestinal bleeding, AKI, and bacterial infections (extra-hepatic decompensating events) [13]. Once patients with cirrhosis develop the first episode of AD, the median survival drops from 12 to less than 2 years.

The PREDICT study (which defined ACLF and AD as per EASL criteria) showed that AD could follow one of the three trajectories in the next 3 months- pre-ACLF (which evolves into ACLF), unstable decompensated cirrhosis (UDC) and stable decompensated cirrhosis (SDC) [156]. Pre-ACLF group patients developed ACLF (as per EASL criteria) and had 3-month and 1-year mortality rates of 53.7% and 67.4%, respectively [156]. Unstable patients required admission but did not develop ACLF and had mortality rates of 21.0% and 35.6%, respectively. Stable cirrhosis did not get readmitted, did not develop ACLF and had a 1-year mortality rate of only 9.5% [156]. The subclassification into these 3 categories was externally validated from a single center in Italy. The group of UDC (the group which most closely resembles the AD group of AARC), was associated with a 1-year mortality rate of about 46%. This group was distinct from SDC and pre-ACLF with a 1-year mortality of 21% and 65% respectively [157]. This was further confirmed in a cohort of HBV-related cirrhosis also. In this cohort, the authors also developed a new model based on precipitant systemic inflammation, which could predict the transition from AD to ACLF [158].

Another study from the PREDICT group (although containing heterogenous precipitants and a different definition of AD from AARC) included 1071 patients with AD-non ACLF and 202 ACLF [159]. They showed that the most common precipitants in AD were bacterial infections and severe alcoholic hepatitis, either alone or in combination. Other precipitants included gastrointestinal bleeding with shock and toxic encephalopathy. About half the patients may not have any identifiable precipitants. The clinical course of acute decompensation was found to be unrelated to the etiology but correlated to the markers of systemic inflammation like CRP and leukocyte count and the number of precipitants. Adequate antibiotic therapy in patients with proven bacterial infections decreased the cumulative incidence of developing ACLF in patients with AD (21.3% vs. 39.2%) and 90-day mortality in both AD (16.9% vs. 36.5%,) and ACLF patients (44.2% vs. 66.2%). Adequate antibiotic therapy in proven bacterial infections can lower organ failures and 90-day mortality [159].

### Differentiating AD from ACLF

ACLF is an entity distinct from acute decompensation. Acute Decompensation occurs in a patient with cirrhosis with or without prior decompensation and is often associated with a precipitant [135]. While AD does not include coagulopathy, ACLF definitions include this.

ACLF and AD can be differentiated by good clinical acumen and clear understanding (Table 2) [20, 160]. There are challenges in the definition of AD and ACLF. AD is excluded in the definition of AARC but included by EASL-CLIF and AASLD [2, 6]. It is very likely that the different societies are characterizing different stages of the same condition [102]. A thorough review of recent literature was conducted, and the experts shared pivotal findings from 2019 to 2023. The AARC database has included only ACLF patients and does not have information on cirrhosis patients presenting with acute decompensation. It is important to compile this data from countries in the Asia–Pacific region and maybe globally.

7. **Recommendations: ACLF and acute decompensation (AD) are distinct**
  7.1. Natural history and outcome of ACLF
    7.1.1. ACLF patients have high 28 and 90 days mortality. Liver transplantation can significantly improve the ACLF patient’s short-term and long-term survival (LoE-High; Recommendation-Strong).
    7.1.2. The outcomes in ACLF are dependent on the stage of the ACLF and associated complications and their dynamic changes during follow-up. (LoE-High; Recommendation-Strong).
    7.1.3. Etiology of ACLF may influence the outcomes. (LoE-Low; Recommendation- Weak).
  7.2. Natural history and outcomes of acute decompensation
    7.2.1. AD is the acute development of complications of cirrhosis like rapid development of ascites, gastrointestinal bleeding, and HE within four weeks of onset and requiring non-elective medical attention. (LoE: Low; Recommendation: Strong).
    7.2.2. Patients with acute decompensation have a mortality rate that is intermediate between patients with ACLF and stable decompensation. (LoE: Low; Recommendation: Strong).
    7.2.3. Systemic markers of inflammation correlate with subsequent development of organ failure. (LoE: Low; Recommendation: Weak).
    7.2.4. Early identification of precipitants and prediction scores can favorably impact the natural course of patients with AD. (LoE: Low; Recommendation: Strong).
  7.3. Differentiating AD from ACLF
    7.3.1. Patients with AD who have or progress to develop extrahepatic organ failure have high short-term mortality. (LoE: High; Recommendation: Strong)
    7.3.2. Any decompensation event preceding jaundice strongly favors AD. (LoE: Moderate; Recommendation: Strong)
    7.3.3. The absence of repeated episodes of decompensation differentiates ACLF as a unique syndrome. This has implications on the management decisions and prognostication, including reversibility of the syndrome. (LoE: Moderate, Recommendation: Strong).
    7.3.4. Long-term survival, reversal, and/or recompensation have been well documented with ACLF. (LoE: Moderate, Recommendation: Strong).

## Portal and systemic hemodynamics and the related complications in ACLF

### Portal, systemic and pulmonary hemodynamics in ACLF

ACLF is hallmarked by the development of acute portal hypertension (PH), a state driven by cytokine production, endothelial dysfunction, and impaired vasorelaxation in addition to the irreversible component of structural resistance. Apart from the structural changes constituting the irreversible component, the marked hepatic inflammation with the generation of reactive oxygen species, especially the superoxide dismutase, decreased nitric oxide bioavailability contributes to the dynamic component of portal hypertension. Increased portal pressure in ACLF, not only contributes to variceal bleeding but also to the development of rapid onset ascites and other systemic complications, including organ failures. ACLF patients have been shown to have a higher HVPG as compared to compensated cirrhosis [161]. It is thus likely that in patients with ACLF, the portal pressure gets acutely elevated and, after recovery, hepatic inflammation and cytokine levels decrease, which leads to improvement in hepatic as well as systemic hemodynamics [161]. Patients with acute onset PH have been shown to have remarkably higher HVPG, increasing risks of variceal hemorrhage and translating to increased 30- and 90-day mortality with the effects being more pronounced in those with alcohol associated-ACLF (AAH-ACLF) [161, 162]. Reduction in HVPG at 3 months leads to improved survival [161]. However, HVPG measurement is invasive and difficult in routine clinical practice. Therefore, non-invasive surrogates that correlate well with invasive HVPG measurements are urgently needed in patients with ACLF [163]. Recent data has focussed on the cardio dynamic state in ACLF wherein the baseline cardiac index is higher in patients with poor outcomes [164, 165]. Mortality in patients with hyperdynamic, hypodynamic, and thermodynamic states was 35%, 25%, and 14%, respectively. Point-of-care echocardiography can be used as a non-invasive test to determine the hemodynamic phenotype in ACLF, especially in the setting of severe sepsis [165]. In addition, remote monitoring of heart rate variability (HRV) shows that reduction in HRV is a surrogate for inflammation in decompensated cirrhosis and predicts progression to ACLF [164].

### Variceal progression, pre-emptive beta blocker, and management of acute variceal bleed

Considering the acute onset portal hypertension, ACLF patients may be more prone to rapid variceal progression and bleeding as sufficient collaterals may not form to decompress acute rise in portal pressure. Indeed, variceal bleed may be seen in up to 25% of them within 90 days with esophageal varices progressing from median grade 1(0–2) to grade 2(2–3) [166]. In the AARC database, of the 72 patients with ACLF who developed AVB, 6-week mortality was 70.8% compared to 54% in those without AVB [155]. The 6-week rebleeding rate was 23%. HVPG in these patients was 25 mmHg compared to 17 in patients who did not develop AVB. None of the patients were reported to undergo TIPS.

**Fig. 9:**
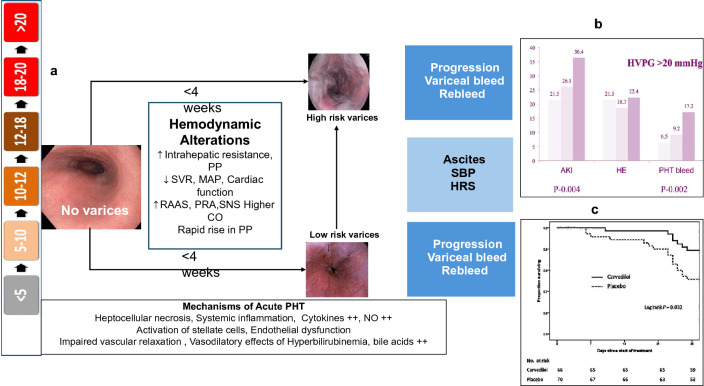
**a** Acute Portal Hypertension syndrome in ACLF, its progression, course and clinical outcomes ACLF. and its course in ACLF. **b** Clinical outcomes in ACLF based on high (> 20 mm Hg) HVPG levels. **c** Reduction in mortality with carvedilol compared to placebo in patients with ACLF. Abbreviations: PP- Portal Pressure, SVR- Systemic Vascular Resistance, MAP- Mean Arterial Pressure, RAAS- Renin Angiotensin Aldosterone System, PRA- Plasma Renin Activity, SNS- Sympathetic Nervous System, CO- Cardiac Output, PHT- Portal Hypertension

Data on the effects of NSBBs on variceal progression and AVB in ACLF is emerging. In a retrospective study by Mookerjee et al., analyzing the data from a subgroup of CANONIC study having ACLF, patients on NSBBs had lower grades of ACLF at presentation and higher resolution (complete or partial) and consequently lower short-term mortality [167]. In the only available randomized control trial in patients of ACLF with no varices or small varices and HVPG > 12 mmHg studying the efficacy and safety of carvedilol, the 28-day mortality was lower in the group receiving carvedilol compared to placebo (10.6% vs. 24.3%, *p* = 0.044). Similarly, variceal progression was seen in a lesser number of patients in the carvedilol group (5/45,11.1%) than in the placebo group (15/46, 32.6%) at the end of 3 months (*p* = 0.021). Moreover, the development of AKI and SBP was lower with carvedilol compared to placebo at 28 days (13.6% vs. 35.7%, *p* = 0.003 and 6.1% vs. 21.4%, *p* = 0.007, respectively) [166] (Fig. 9). Initiation and use of beta-blockers in ACLF have been shown to ameliorate systemic inflammation and reduce mortality in multiple diverse cohorts irrespective of impact on the prevention of variceal bleeding or infections [166, 167]. However, only one in four patients with ACLF are eligible for NSBB [30]. NSBBs should be withheld in patients with severe circulatory dysfunction as evidenced by systolic BP < 90 mm Hg, serum Na < 130 mEq/L, or AKI [168]. Considering the greater hypotensive effect of carvedilol, future studies with other nonselective beta-blockers, such as propranolol or nadolol, should be encouraged. Few studies have reported that the addition of midodrine in patients receiving beta-blockers can prevent variceal bleeding and lead to a reduction in HVPG, but the rationale has not yet been defined [169, 170]. Further studies are required to validate these findings, as midodrine is an alpha agonist that increases vascular tone and blood pressure, and beta blockers reduce mean arterial pressure.

Primary prevention of AVB by drugs or endotherapy for large or high-risk varices in ACLF patients still lacks consensus. Being sick, the NSBB tolerability and risk of post EVL ulcer bleed raise genuine concerns. Endoscopic therapy is the primary modality to achieve hemostasis. Pre-emptive TIPS has been shown to improve survival in ACLF with AVB [171]. Patients with ACLF have a higher risk of post-EVL ulcers [172]. SX-Ella Danis stent (DE stent) and rescue TIPS are the potential rescue options in refractory AVB or post-EVL ulcer bleed. In a recent propensity-matched analysis of patients with refractory VH, rescue TIPS was associated with lower six-week mortality than DE stent (20.6% vs 50%). In those undergoing TIPS, placement of TIPS within 8-h compared with 8–24 h was associated with lower 6-week (48.6% vs. 12.9%; *p* = 0.003) and 1-year mortality (62.9% vs. 16.1%, *p* = 0.001) despite comparable rebleed rates (2/31; 6.5% vs. 2/35;5.7%; *p* = 0.90) [173]. However, TIPS in patients with APASL-identified ACLF is challenging as these patients have liver failure and cannot be recommended in the absence of immediate transplant prospects.

### Management of ascites and paracentesis-induced circulatory dysfunction (PICD)

Ascites are one of the syndromes defining components and in the AARC study, about 91% of patients had ascites at presentation. The rapid development of ascites is reflective of the acute development of PH in ACLF. Compared to ascites of decompensated cirrhosis, those with ACLF tend to have limited flank fullness, reduced compliance of the abdominal cavity, and are likely to have an abdominal compartment syndrome-like effect.

Restriction of dietary salt and careful use of diuretics remain first-line therapy, but deranged systemic hemodynamics frequently lead to diuretic-induced complications in more than 50% of the patients [174]. The addition of midodrine has been shown to improve tolerability in a pilot study [174].

**Fig. 10:**
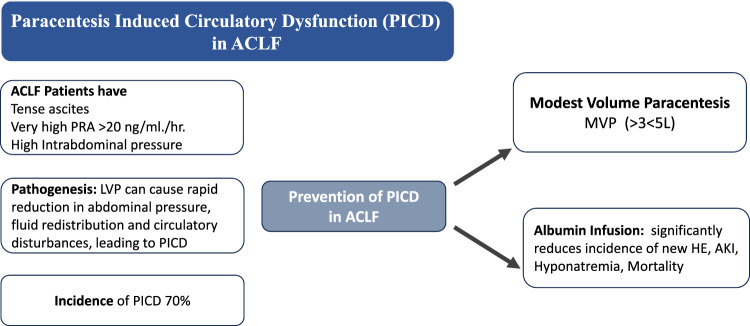
Paracentesis induced circulatory dysfunction (PICD) and relevance of Modest Volume Paracentesis in ACLF

About one-third of the patients presenting with ACLF do require paracentesis for severe-grade ascites [175]. Paracentesis is safe in ACLF with hemorrhagic complications in ≤ 3%, with lower fibrinogen levels (< 0.7 g/L) being a potential predictor [176]. Paracentesis-induced circulatory dysfunction (PICD) is common in ACLF, developing in almost three-fourths (70%) of those undergoing even less than 5 L of paracentesis [modest volume paracentesis (MVP)]. PICD is clinically manifested as faster re-accumulation of ascites, hyponatremia, renal impairment, and shorter survival. Laboratory diagnosis relies on an increase in Plasma Renin Activity (PRA) > 50% to a PRA of > 4 ng/ml/h on day 5 or 6 as compared to baseline. A high PRA level in ACLF patients reflects a state of severe systemic inflammation, high portal pressure and systemic circulatory dysfunction [175]. Development of PICD in ACLF is associated with very high mortality. Volume expansion with albumin can prevent PICD and improve survival, and is required even in those ACLF patients undergoing MVP (Fig. 10) [175]. A small pilot randomized study reported midodrine as an alternative to albumin to prevent PICD in patients with ACLF undergoing MVP with similar PICD rates (20% vs 16%, *p* = 0.85) [177].

### Hepatic encephalopathy: prevention, therapy and implication in natural history of ACLF

**Fig. 11:**
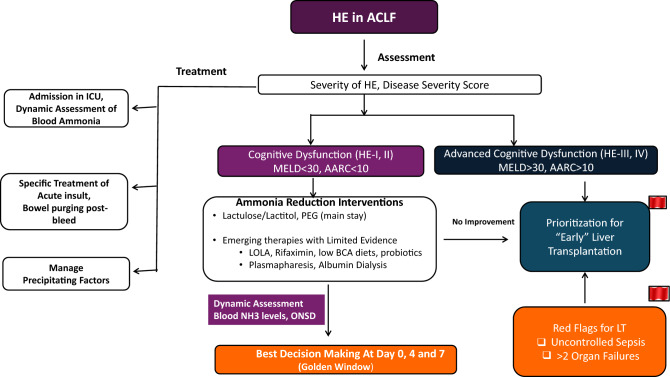
Algorhtmic approach to the management of hepatic encephalopathy in ACLF

Approximately 40% of patients with ACLF have HE [72]. Development of HE indicates poor prognosis in patients with ACLF. The presence of grade III-IV HE is associated with higher mortality in comparison to grade I-II. Further progression from grade I-II HE to grade III-IV HE is associated with increased mortality. The window period between the transition from no HE to HE and grade I-II to grade III-IV needs to be identified clearly from a treatment perspective. Management of grade III-IV HE in hospitalized patients requires admission to the ICU and includes—(1) identification and treatment of precipitating factors, including infections, and (2) specific measures for decreasing hyperammonemia and systemic inflammation. Inflammation plays a greater role in the pathogenesis of HE in patients with ACLF than in patients without ACLF and is associated with a cytokine storm. Newer modalities of treatment, like plasma-exchange therapy, or cytokine filters have shown improvement in outcome in acute liver failure outcomes, especially if instituted early in the course of treatment (grade I-II HE) (Fig. 11). A recent AARC data reported that plasma exchange (PE) improves systemic inflammation and lowers the development of MOF in patients with ACLF [178]. Further studies should be directed towards timing of PE, high volume versus low volume, and improvement or non-progression of HE in ACLF. Recently analyzed AARC data showed that ammonia was significantly and persistently high in patients with grade III and IV HE (*p* < 0.001). Further dynamic change in ammonia level correlates well with the clinical course of HE. However, ammonia-targeted therapy needs further trials and validation. In a recent randomized study, it was reported that the addition of rifaximin to ACLF patients on intravenous antibiotics and lactulose did not lead to a survival advantage [179]. Till further studies become available, lactulose remains the mainstay of therapy.

8. **Recommendations: portal and systemic hemodynamics in ACLF**
  8.1. Systemic, hepatic, and pulmonary hemodynamics in ACLF
    8.1.1 Baseline HVPG is an important predictor of mortality in ACLF. (LoE: Moderate; Recommendation: Strong)
    8.1.2 The reduction in HVPG significantly influences the outcome of ACLF patients. (LoE: Moderate; Recommendation: Weak)
    8.1.3 HVPG measurement is safe in ACLF. However, non-invasive surrogates of HVPG need to be investigated for serial assessment of portal pressure (LoE: Moderate; Recommendation: Strong)
    8.1.4 Point-of-care ultrasound (POCUS) guided hemodynamic and cardiac assessment is recommended for managing patients with ACLF and critical illness. (LoE: High; Recommendation: Strong)
  8.2. Variceal progression, pre-emptive BB therapy and managing acute variceal bleed
    8.2.1. Acute onset portal hypertension is a hallmark of ACLF leading to rapid growth of varices and increased risks of variceal hemorrhage and progression of ascites translating into detrimental downstream effects (LoE: Moderate; Recommendation: Strong).
    8.2.2. NSBBs like propranolol and carvedilol should be used in patients of ACLF with no or small varices with HVPG > 12 mm Hg or with evidence of clinically significant portal hypertension in the absence of any contraindications to NSBB. (Level of evidence: Strong, Grade of Recommendation: Strong).
    8.2.3. For acute variceal bleeding in ACLF, endoscopic therapy combined with vasoactive agents remains the primary modality. (Level of evidence: Strong, Grade of Recommendation: Strong).
    8.2.4. Pre-emptive TIPS can be considered in selected cases with failure to achieve hemostasis after first endoscopic therapy. (Level of evidence: Low, Grade of Recommendation: Weak).
    8.2.5. Rescue TIPS (within 8 h) may be preferred over Danis Ella stent for refractory variceal hemorrhage (LoE: Moderate; Grade of Recommendation: Weak)
    8.2.6. Nonselective beta-blockers improve outcomes in ACLF but should be used with caution. They should be avoided in the presence of severe circulatory dysfunction (systolic BP < 90 mm Hg, serum Na < 130 mEq/L or AKI). (LoE: Moderate; Recommendation: Strong).
  8.3. Management of ascites and PICD
    8.3.1 Dietary salt restriction and diuretics remain the first-line therapy in ascites management. The use of midodrine may improve the tolerability of diuretics. (LoE: Moderate; Recommendation: Weak)
    8.3.2 Paracentesis is safe in ACLF but is associated with a high incidence of PICD even with modest volume paracentesis (MVP). (LoE: Moderate; Recommendation: Strong)
    8.3.3 PICD is clinically manifested as faster re-accumulation of ascites, with development of hyponatremia, and renal impairment leading to a shorter survival. Laboratory diagnosis relies on an increase in PRA by > 50% to a PRA of > 4 ng/ml/h on day 5 or 6 as compared to baseline (LoE: High; Recommendation: Strong)
    8.3.4 Measurement of PRA is helpful in the management of ascites in clinical practice, but is not recommended for routine use. (LoE: Low; Recommendation: Strong)
    8.3.5 Albumin at a dose of 8 g/L of ascitic fluid tapped is recommended for patients undergoing ≥ 3 L of tapping. (LoE: High; Recommendation: Strong)
    8.3.6 Midodrine is as effective as albumin in preventing PICD in patients with ACLF. (LoE: High; Recommendation: Strong)
  8.4. Hepatic encephalopathy: prevention, therapy and implication in natural history of ACLF.
    8.4.1. About a third of patients with ACLF have HE at diagnosis. (LoE: Moderate; Recommendation: Strong).
    8.4.2. HE is associated with increased mortality independent of other organ failures; the mortality is higher in grades 3–4 (organ failure) than in grades 1–2 (organ dysfunction). (LoE: High; Recommendation: Strong).
    8.4.3. Inflammation and ammonia play a key role in the pathogenesis of HE in ACLF. (LoE: High; Recommendation: Strong).
    8.4.4. Blood ammonia level is elevated in HE in ACLF but its correlation with severity and prognosis needs to be further investigated (LoE: Moderate; Recommendation: Strong).
    8.4.5. Identify and treat underlying precipitant events. Lactulose is the only recommended drug for HE. (LoE: High; Recommendation: Strong).
    8.4.6. Patients on broad-spectrum antibiotics do not benefit from the addition of rifaximin. (LoE: Low; Recommendation: Weak).
    8.4.7. Advanced HE in patients with ACLF needs prioritization for liver transplant. ((LoE: Moderate; Recommendation: Weak).

## Role of liver histology in ACLF

### Diagnostic implications of liver biopsy in ACLF

Liver biopsy in ACLF can aid in accurate diagnosis. However, there is a paucity of evidence to support that it improves the overall outcome of patients. Liver biopsy in ACLF may be of help in differentiating drug-induced liver injury from autoimmune hepatitis and differentiating acute liver failure from ACLF. In addition, liver biopsy is needed for certain etiologies, especially alcohol-associated hepatitis (AH), severe autoimmune hepatitis (AIH), and flare of Wilson disease. In AIH, the biopsy is particularly helpful when antibodies and IgG are negative, but there is a high index of suspicion. In these recent studies, DILI as an acute and alcohol as acute and chronic underlying etiology are being increasingly reported in patients of ACLF which are preventable causes. In a recent study of 184 patients with AH, ductular bilirubinostasis was more frequent in patients of ACLF than in those without ACLF but without any prognostic significance [180]. In the AARC database, DILI was a common cause (10.5%), with a biopsy done in 27% of the patients with ACLF-DILI cases. In another study of 1,666 patients with cirrhosis, 68% had used complementary and alternative medicine (CAM), and 35.7% (*n* = 30/84) of patients presented with possible CAM-DILI-ACLF [107]. Portal-based neutrophilic, eosinophilic predominant mixed inflammation, hepatocyte ballooning, autoimmune-like features, and severe cholestasis were seen in liver biopsies of patients with CAM-induced ACLF [181].

The preferred route of liver biopsy in patients with ACLF is transjugular, as a percutaneous approach is not feasible due to coagulopathy and ascites, and the laparoscopic route is impractical and carries risks in sick patients. Noninvasive tools to measure liver stiffness and biomarkers may help in identifying patients with advanced fibrosis. Studies are needed to validate the performance of these tests in the setting of ACLF.

### Prognostic implications of liver biopsy in ACLF

The alcoholic hepatitis histologic score (AHHS) score, based on the histology, is helpful in the prognostication of alcoholic steatohepatitis cases [182]. Similarly, the high density of ballooning degeneration and Mallory-Denk bodies have been suggested to indicate poor response to steroid therapy [183]. In another study including 152 patients with the diagnosis of ACLF (EASL-CLIF criteria) due to alcohol, sepsis, hepatotropic viruses, and unknown causes reported severe hepatic necrosis, dense lobular inflammation including ceroid-laden macrophages, and lack of advanced Ishak fibrosis stages (Ishak stages 5–6) as predictors of poor prognosis compared to those with decompensated cirrhosis [184]. However, only dense lobular inflammation, including ceroid-laden macrophages, were independent predictors of short-term mortality [184, 185]. Further studies are required to validate these findings, as histologic scoring has not been reported to be superior to simple prognostic scores such as MELD and mDF [186].

9. **Recommendations: role of liver histology in ACLF**
  9.1. Diagnostic implications of liver biopsy in ACLF
    9.1.1. Liver biopsy plays an important role in distinguishing ACLF from ALF. (LoE- High, Recommendation-Strong).
    9.1.2. Liver biopsy is helpful in patients in whom the presence and stage of underlying chronic liver disease (CLD) and/or the cause of chronic liver disease are unclear. (LoE- High, Recommendation-Strong).
    9.1.3. A trans-jugular liver biopsy is helpful in diagnosing/confirming the cause of acute injury, especially in patients with unclear clinical diagnostic pointers in patients with ACLF. (LoE- High, Recommendation-Strong).
    9.1.4. Drug-induced liver injury and alcohol-associated hepatitis are emerging as the most common acute etiologies for ACLF, and both require liver biopsy-based diagnostic confirmation. (LoE- Moderate, Recommendation- Weak).
    9.1.5. The need for liver biopsy in ACLF should be individualized, especially in alcohol-associated hepatitis, severe autoimmune hepatitis, and flare of Wilson disease. (LoE- Moderate; Recommendation- strong).
    9.1.6. Standardization of liver biopsy assessment is essential for a uniform approach to the diagnosis and treatment of acute and chronic insult in ACLF (LoE: low, Recommendation: Weak).
  9.2. Prognostic implications of liver biopsy in ACLF
    9.2.1. Liver biopsy in ACLF especially in alcohol-associated hepatitis, may aid in prognostication. (LoE- Low, Recommendation-Weak).
    9.2.2. Histological features such as ductular bilirubinostasis, cirrhosis, necrosis, eosinophilic degeneration, and ballooning degeneration indicate relatively poor outcomes in ACLF patients. (LoE- Moderate, Recommendation- Weak).
    9.2.3. Liver histology in conjunction with clinical severity scores should be used for prognostication of patients with ACLF. (LoE—Moderate, Recommendation-Strong).

## Treatment of ACLF

**Fig. 12:**
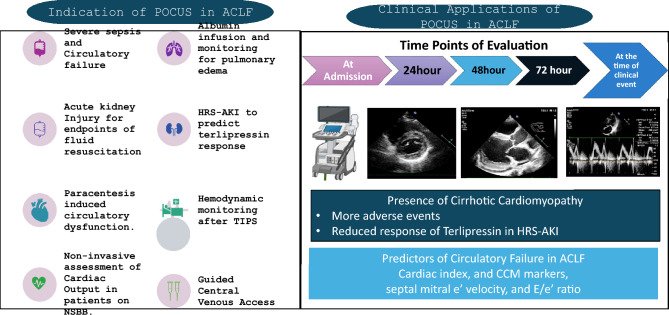
Point of Care Ultrasound (POCUS) in the management of ACLF patients

**Fig. 13:**
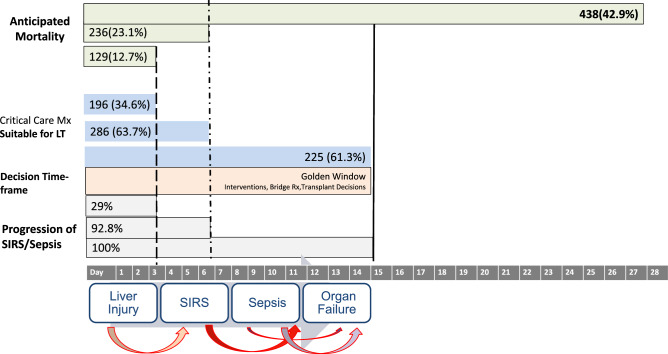
The time frame, Golden window for medical management and Liver Transplant Decision in ACLF

Patients with ACLF, generally require to be hospitalized and monitored carefully. A dedicated liver ICU with a multidisciplinary team is helpful for the management of these patients. A careful assessment of the degree of liver failure, extra-hepatic organ failure (s), infection, sepsis, fluid deficits, and requirements, should be assessed in the first six hours. Point of care ultrasound (POCUS) could be a very useful modality during this period (Fig. 12). During the first 24 h itself, the need for a liver transplant should also be assessed. The Golden Window for ACLF worsening, stabilization or reversal and the time frame for selection for liver transplant is seven days (Fig. 13). All efforts should be made to select patients who are likely to get the benefit of bridge therapy and the chances such therapies could provide for the success of non-transplant options and even improving the outcome of liver transplant. The ICU teams should be very cautious in the use of fluids, albumin, and vasopressors so as not to make a potential and good transplant candidate into a difficult and risky transplant.

### Nutritional assessment and interventions

Nutrition has a pivotal role in patients with ACLF. Patients with ACLF should be evaluated with the existing tools, i.e., Nutrition Risk Screening 2002 (NRS 2002), Royal Free Hospital Nutritional Prioritizing Tool (RFH-NPT) and Malnutritional Universal Screening Tool (MUST). Nutrition in Critical Ill score (NUTRIC) can be used for patients with ACLF admitted to the ICU. A study including 431 patients of HBV-related ACLF reported myosteatosis in 60% of patients and sarcopenia in 20.% of patients using non-contrast CT assessment at the L3 vertebra [187]. A skeletal muscle index of ≤ 40.2 cm/m^2^ in men and ≤ 31.6 cm/m^2^ in women is considered sarcopenia. Myosteatosis was defined as skeletal muscle radiation attenuation < 41 Hounsfield unit (HU) in patients with a BMI < 25 kg/m^2^ and SM-RA < 33 HU in patients with a BMI ≥ 25 kg/m^2^. The authors reported that on competing risk analysis (for LT), age, AARC score, and sarcopenia were associated with 90-day mortality.

Patients with liver failure should have a target daily intake of 30–35 kcal/kg body weight and daily protein intake of 1.2 to 1.5 g protein/kg body weight [188]. It is prudent to increase the intake to prevent refeeding syndrome gradually. A low caloric intake (< 21.5 kcal/kg body weight/day) is associated with increased mortality, especially in alcohol-related ACLF, and should be avoided [189]. Although enteral nutrition is preferred to maintain mucosal integrity and reduce the risk of bacterial translocation, patients on high-dose vasopressors should avoid enteral nutrition as it may precipitate digestive complications (vomiting, diarrhea, etc.) and starting parenteral nutrition to switch to enteral nutrition once shock improves is preferred [190].

An RCT including 90 patients with ACLF (mean AARC score 8) admitted to wards reported a significant reduction in systemic inflammation, endotoxemia, and sepsis with omega-3 fatty acid supplementation [191]. Omega-3 FAs led to a decrease in endotoxin levels, prevented an exacerbated rise in TNF-α and IL-6, and suppressed IL-1β, whereas omega-6 FAs prevented the rise in endotoxin levels and TNF-α. Furthermore, omega-3 led to an increase in expression of TLR2 and TLR4, which are well known to recognize lipopolysaccharide (endotoxin) as a pathogen-associated molecule. This led to a reduction in the incidence of sepsis in the omega-3 fatty acid group. The dose of omega-3 fatty acids used was 100 ml per day infused over a period of 6 h. Although the role of micronutrients in ACLF has not been investigated specifically, thiamine and zinc supplementation in alcohol-related ACLF should be ensured [192]. Branched-chain amino acids (BCAA) supplementation in ACLF has not been proven to be effective in patients with ACLF [193]. A carbohydrate-containing late-evening snack in patients with ACLF has been shown to reduce fat oxidation and improve carbohydrate oxidation and resting energy expenditure [194].

### Antiviral strategies in ACLF HBV reactivation

The World Health Organization has recently published the Hepatitis B guidelines [195]. The suggested inclusions for treatment include (a) patients with significant fibrosis (APRI > 0.5 or LSM > 8) irrespective of DNA levels, (b) DNA > 2,000 IU/ml and ALT > ULN; (c) all patients with coinfections, diabetes mellitus, MAFLD, (d) those with family history of hepatocellular carcinoma or cirrhosis; (e) all those on immunosuppression and (e) those with extrahepatic manifestations due to HBV (vasculitis or glomerulonephritis). With these expanded criteria, most patients with ACLF would require antivirals. The median HBV DNA levels in patients with HBV ACLF are usually higher and prompt treatment with antivirals would lead to improved survival [196]. Although the currently available drugs (TAF, TDF, Entecavir) are equally effective for HBV, a single-center trial reported TAF to be superior to TDF with lower renal dysfunction [197, 198]. A decline in HBV DNA levels by log 2 at two weeks has been reported to improve the severity scores and survival [196].

### Corticosteroids in alcohol-associated ACLF

**Fig. 14:**
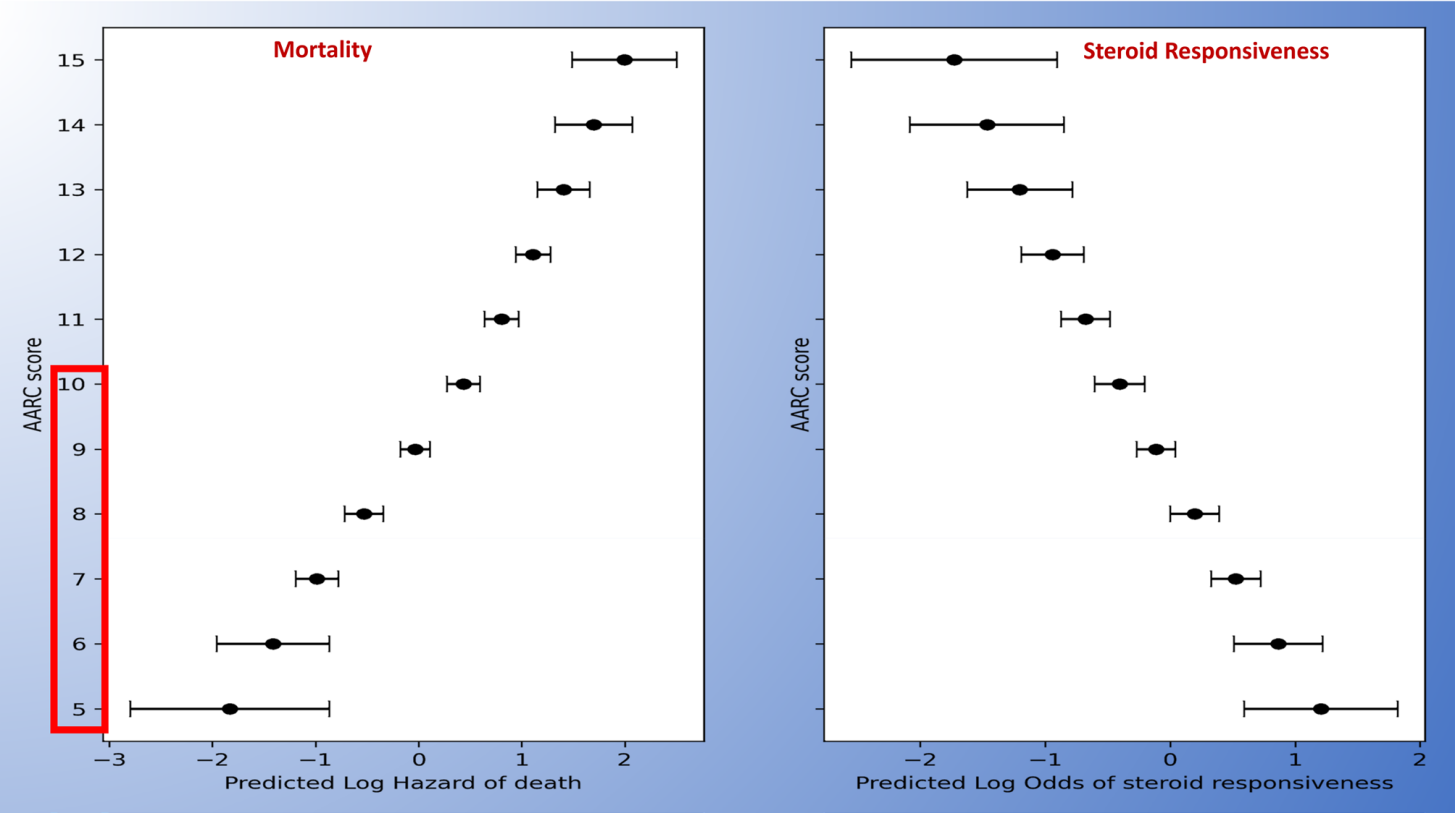
AARC score as a guide to assess predictor of steroid response and mortality in patients with alcohol-ACLF

AAH can be diagnosed based on NIAAA clinical criteria, and trans jugular liver biopsy is suggested in the presence of confounding factors for those presenting as ACLF [199]. Corticosteroids have been reported to improve short-term survival irrespective of the type of steroids used in patients with MELD scores between 25 and 39 [200]. However, the incidence of infections and extrahepatic organ failures is higher in patients with alcohol-related ACLF [201]. Of the 1249 patients with severe AAH with ACLF in the AARC database, only 211 (17%) could be considered for corticosteroid therapy. The cumulative response rate (Lille score) was 48%, and the response was inversely correlated with the ACLF grade, bacterial infections, and extrahepatic organ failures [202]. Patients with an AARC score above 10 at baseline were poor responders to corticosteroids and had higher mortality (Fig. 14). Similarly, a subgroup analysis of the STOPAH trial reported poorer response to corticosteroids in advanced ACLF grades [203]. Prophylactic antibiotics (norfloxacin and amoxicillin clavulanate) can reduce the incidence of bacterial infections but without survival benefit [136, 204].

### Corticosteroids in DILI and AIH-ACLF

Steroids are the first line of drugs for the treatment of patients with AIH. There is a paucity of data on the use of steroids in patients with AIH-ACLF. The largest dataset published on this is the AARC database, which included 82 patients with AIH-ACLF [119]. Among these patients, 28 (34%) received steroids, while the remaining 54 patients, half of whom had concomitant sepsis did not receive steroids. Subsequent analysis revealed that patients with advanced age, a MELD score > 27, the presence of HE, and hepatic fibrosis (≥ F3) had a poor response to steroids [119]. Another study with a limited sample size study of 29 AIH-ACLF patients suggested the potential benefits of steroids in those with no extrahepatic organ failure, with a MELD score < 24 and a CTP score < 11. Notably, 41% of these patients developed sepsis after the initiation of steroid therapy [205].

While steroids play a crucial role in managing autoimmune hepatitis, their utility in ACLF warrants careful consideration, especially given the challenges associated with patient selection, timing, and dosage [206]. Prospective studies are needed to establish guidance for steroid use in AIH-ACLF, considering various factors such as disease severity, patient characteristics, and the potential risks associated with steroid therapy.

The primary treatment of DILI includes withdrawal of the offending drug and supportive care. N-acetylcysteine (NAC) is the antidote for acetaminophen overdose. NAC may also be effective for non-acetaminophen-related ALF. A 3-day intravenous NAC has been shown to improve overall survival and transplant-free survival in patients with idiosyncratic DILI-induced ALF [207]. Another randomized study revealed NAC was associated with a shorter length of stay but no survival benefit in patients with ALF due to antitubercular DILI [208]. Despite the common use of steroids in DILI, existing studies are predominantly retrospective, with arbitrary inclusion and exclusion criteria, and show mixed results. Steroids may be beneficial in a small proportion of patients with biopsy-proven evidence of drug-induced AIH, especially with significant duct loss, and in those with immune checkpoint inhibitor-induced liver injury [107, 209, 210].

### Emerging therapies in ACLF-anti-inflammatory, immune-modulative, regenerative and others:

#### Fecal microbiota transplant (FMT)

ACLF is a condition associated with significant intestinal dysbiosis in the context of microbial composition, richness, diversity, and functional metabolism. Specific gut microbiota(GM) patterns are present in patients with ACLF, while characteristic microbial metabolites are associated with ACLF development [211]. Intestinal dysbiosis featuring increased relative abundance of Enterococcus was found to be associated with progression of hepatitis B (HBV)-related ACLF and initiation and progression of severe alcohol-related hepatitis (SAH) [212, 213]. Specific GM (e.g., Lachnobacterium with HE, Pediococcus with death post steroid therapy), their interactions (e.g., Enterococcus and Acinetobacter with AKI), and metabolites (e.g., glycerophospholipid-metabolism with death) were associated with complications of SAH and treatment outcomes [214]. In this context, fecal microbiota transplantation (FMT) aims to modulate dysbiotic GM through protocolized beneficially but non-standardized infusion of screened and processed fecal suspension from apparently healthy donors into the gastrointestinal tract through various routes (endoscopic, naso-duodenal or -jejunal feeding tube, colonoscopic or rectal enema). Interventions in SAH patients primarily drive our current understanding of outcomes related to FMT in ACLF. A pilot study on FMT compared to matched healthy controls in steroid-ineligible SAH demonstrated improved survival at one year in the former (87.5% vs 33.3%) [215]. A case–control study and another randomized controlled trial showed that liver-related clinical events and survival rates were better with FMT than with corticosteroid (90-day 75% vs 56.6%) or pentoxifylline (83% vs 56%) treatment [216, 217]. The use of FMT, specifically in SAH-ACLF, has shown promise in improving long-term survival, amelioration of portal hypertensive complications and life-threatening infections, and reducing alcohol misuse [218]. In all studies that examined the dynamics of GM, clinically evident benefits of FMT paralleled significant microbial changes. The pertinent changes included an increased relative abundance of Bacteroides, Bifidobacterium, and Lachnospira and a reduction in pathogenic Proteobacteria and Tenericutes. Similarly, the functional GM profiling among survivors revealed significant changes to short-chain fatty acid production, bile acid and lipopolysaccharide synthesis pathways, and amelioration of pro-inflammatory signaling pathways [219]. The clinical utility and outcomes of FMT in other etiologies of ACLF remain unproven.

#### Regenerative and cell-based therapy

Exponential increase in hepatocyte death without compensatory regeneration accounts for liver failure and sudden onset of hepatic decompensation in ACLF [220]. Loss of supportive regenerative niche due to depleted immune function for clearing injury, inflammation, and infection is evolving as the underlying cause of regeneration failure in ACLF [221]. Hence, promoting liver repair by modulating the liver microenvironment and/or restoring immune function can serve as a potential therapeutic approach for the management of ACLF (in which uncontrolled injury and inflammation contribute significantly to morbidity and mortality) [221]. The bone marrow (BM) and its progeny contain several candidate cell types for this application, including hematopoietic progenitor cells, MSCs, and macrophages. Therapeutic use of G-CSF mobilized BM stem cell therapy in ACLF (particularly ACLF patients satisfying APASL criteria) has shown to be promising in terms of improvement in overall survival, liver function, and the MELD score [222]. However, studies from the European cohort of ACLF did not demonstrate survival benefits, probably due to the inclusion of patients with sepsis and organ failures [223, 224]. Nevertheless, G-CSF theoretically can lead to the improved regenerative capacity of the liver, and further studies in combination with TLR4 inhibitors are being planned. [225] Mesenchymal stem cells (MSCs) have been reported to be safe in patients with ACLF in early phase trials; however, their efficacy in real-world studies is lacking, which merits further studies. [226, 227]

#### Liver dialysis, plasmapheresis and other artificial liver support system

Plasma exchange is a preferred modality of therapy for patients with ACLF, irrespective of etiology, especially in Asian centers. There have been several studies reporting therapeutic plasma exchange (TPE) as a bridging modality or a non-transplant measure to improve survival. TPE in patients with ACLF has been reported to reduce (or contain) systemic inflammation and improve the monocyte phagocytic function and mitochondrial respiration and survival compared to those who received standard medical therapy [178]. A systematic review and meta-analysis, including 20 studies and 5705 patients with ACLF, reported improved 30 (RR 1.36, 95% CI 1.22–1.52, *p* < 0.001) and 90-day (RR, 1.21, 95% CI 1.10–1.34, *p* < 0.001) survival but did not reach statistical significance on sensitivity analysis including only RCTs [228]. Nevertheless, TPE can provide a window of 30 days to bridge a patient with ACLF to LT [178, 228–236] (Table 7). Centrifugal TPE is preferred over membrane TPE. The number of sessions required is individualized and the role of high volume and standard volume needs to be compared in RCTs. The role of MARS and other artificial liver support systems in ACLF remains limited and needs further studies.

10. **Recommendations: treatment of ACLF**
  10.1. Nutritional assessment and interventions
    10.1.1 Conventional tools should be used for assessing the nutritional status of ACLF patients (LoE: Moderate; Recommendation-Strong)
    10.1.2 ACLF patients must receive 30–35 kcal/kg/day (actual body weight corrected for ascites) or 1.3*REE /Kg (IBW as per height) and 1.2–1.5 g/kg of protein per day (LoE- High; Recommendation-Strong), preferably through the oral route.
    10.1.3 Enteral nutrition through nasogastric/nasojejunal tube is suggested if the individual fails to achieve a caloric intake of 21 kcal/kg/day per oral (LoE- Moderate; Recommendation-Strong)
    10.1.4 Carbohydrate-rich, late-evening snack is suggested for patients with ACLF (LoE: Moderate; Recommendation-Strong)
    10.1.5 In critically ill patients’ data regarding low vs. high protein diet is limited with the absence of high-quality trials. (Low LoE; weak recommendation)
    10.1.6 Omega 3 fatty acid infusions may be used in patients with ACLF for the prevention of sepsis. (LoE; Moderate; Recommendation: Strong)
    10.1.7 In ICU patients, energy requirements should be assessed by an indirect calorimeter (IC). If IC is not available, then hypocaloric nutrition (< 70% of estimated needs) should be preferred for the first week of the ICU stay. (LoE: Moderate; weak recommendation)
  10.2 Antiviral strategies in ACLF-HBV
    10.2.1 Nucleos(t)ide analogs should be started immediately in all HBV-infected ACLF patients at presentation while waiting for confirmation by HBV DNA level. (LoE: High; Recommendation: Strong)
    10.2.2 Potent antiviral drugs, such as tenofovir alafenamide, tenofovir disoproxil fumarate or entecavir should be used. (LoE: High; Recommendation: Strong)
    10.2.3 A 2-log reduction in HBV DNA levels after 2 weeks of therapy reduction is associated with improved survival and should be performed whenever feasible. (LoE: Moderate; Recommendation: Strong)
  10.3. Corticosteroid in alcohol-associated ACLF
    10.3.1 Corticosteroids are recommended for alcohol-related ACLF who have evidence of alcohol-associated hepatitis in the absence of contraindications. (LoE- High, Recommendation- Strong).
    10.3.2 Patient selection for a short course of steroid therapy in alcohol-associated ACLF should be carefully made, using scoring systems like MELD and AARC to identify those who may benefit most in the setting of alcohol-associated hepatitis. (LoE- Moderate, Recommendation- Strong).
    10.3.3 Monitoring for and managing potential complications, such as infections and gastrointestinal bleeding, should be done when using steroids in patients with alcohol-related ACLF. Prophylactic antibiotics are not universally recommended. (LoE- High, Recommendation- Strong).
  10.4. Corticosteroid in DILI and AIH-ACLF
    10.4.1 Corticosteroids may be used in patients with AIH-related ACLF who have no contraindications for steroid therapy or those who do not have overt HE. (LoE- Moderate, Recommendation- Strong).
    10.4.2 Steroids may be used in patients with biopsy-proven drug-induced ACLF who have evidence of AIH. (LoE- Low, Recommendation- Weak)
    10.4.3 Close monitoring is suggested for patients who receive steroids in ACLF. (LoE- Low, Recommendation- Strong)
    10.4.4 A short course (of 4 weeks) of steroid therapy may be suggested, but the duration of therapy is based on clinical recovery and clinicians’ judgment. (LoE- Low, Recommendation- Weak)
11. Emerging therapies in ACLF-anti-inflammatory, immune-modulators, regenerative and others
  10.5.1. Fecal microbiota transplant
    10.5.1.1. FMT-based beneficial modulation of gut dysbiosis may improve short- and long-term clinical outcomes in patients with alcohol-associated ACLF, especially those who are deemed ineligible for standard care or curative liver transplantation. (LoE- Moderate, Recommendation- Weak).
    10.5.1.2. Currently, FMT is not recommended in the treatment of ACLF of any etiology other than alcohol-associated ACLF. Its role as a bridge to liver transplantation requires validation from well-designed, pragmatic methodology-driven prospective studies. (LoE- Moderate, Recommendation-Strong).
  10.5.2. Regenerative and cell-based therapy
    10.5.2.1. G-CSF and MSCs may be used as experimental therapies in patients who have no transplant prospects. (LoE—Moderate, Recommendation—Weak).
  10.5.3. Liver dialysis, plasmapheresis and other artificial liver support system
    10.5.3.1. Therapeutic Plasma exchange may be performed in patients with ACLF as a bridging modality to liver transplant. The number of sessions and type of plasma exchange should be based on clinical judgment. (LoE: Moderate; Recommendation: Strong).
    10.5.3.2. Currently available artificial liver support systems, including MARS, have not shown survival advantage and are hence not recommended in ACLF patients. (LoE: Low; Recommendation: Strong).
    10.5.3.3. Use of cytokine, bile acid, and bilirubin filters can be considered in special situations. More data is however required. (LoE: Low; Recommendation: Weak)

**Table 7:** Bridge therapies for ACLF patients

Studies	ACLF definition	Artificial liver support	Control	Type of study	Outcome
Kribben et al. 2012 [229]	EASL	FPSA (n-77)	SMT (n-68)	RCT	No difference in 28 days and 90 days mortality
RELIEF Trial, 2013 [230]	–	MARS (n-95)	SMT(n-94)	RCT	No difference in 28 days morality
Gerth et al. 2017 [231]	EASL	MARS(n-47)	SMT (n-54)	Retrospective	No difference in 28 days morality
Bañares et al. 2019 [232]	Multiple	MARS (n-91)	SMT (n-74)	IPD meta-analysis	No difference in 30 days mortality
Maiwall et al. 2021 [178]	APASL	TPE-94	SMT-89	PS matched Retrospective	Improved survival at 28 days but not at 90 days
Kumar et al. 2022 [233]	APASL	Low volume TPE-21	SMT-29	Retrospective	Improved survival at 28 days but not at 90 days
Ramakrishnan et al. 2022 [234]	EASL	TPE-14	SMT-14	Prospective non-randomized	Improved survival at 28 days but not at 90 days
Agarwal et al. 2023 [235]	EASL	DIALIVE (n-17)	SMT (n-15)	RCT	No difference in 28 days morality
Swaroop et al. 2023 [236]	EASL	TPE-38	SMT-38	PS matched Retrospective	Improved survival at 28 days but not at 90 days
Beran et al. 2024 [228]	Multiple Meta-analysis	TPE-2243	SMT-2210	Meta-analysis	Improved survival with TPE

## Prognostic models in ACLF

The outcome of patients with ACLF is often driven by the severity of organ failures, and severity scores such as MELD and MELD-Na may underestimate mortality among patients with ACLF [237]. Accurate prognostication in ACLF requires scores that truly capture the entire clinical course at the time of measurement, thus assessing not only hepatic (liver, coagulation, brain) but also extrahepatic organ failures (cardiovascular, respiratory, and renal), manifestations of portal hypertension (e.g. HE) and complementary variables that capture disease severity (e.g. lactate). Three general ACLF scores include the evaluation of extrahepatic organ failures. NACSELD ACLF score includes advanced extrahepatic organ failure defined by the need for organ support (mechanical ventilation, RRT, and vasopressors) [238]. CLIF-C ACLF score evaluates all six organ/systems. It includes the CLIF-C OF score, a largely validated adaptation of the SOFA score to the cirrhotic population, in addition to age and WBC count [91]. The AARC score includes serum bilirubin, serum creatinine (sCr), serum lactate, INR, and HE [86]. MELD-lactate combines MELD score and lactate [77]. In a study comparing several models at admission looking at patients in the APASL region, model performance characteristics (discrimination, C statistic) for AARC score, CLIF-C (ACLF score, and OF score), NACSELD-ACLF model and MELD-Lactate, were similar [29]. Further studies on calibration are needed across populations. At admission, both the AARC score and MELD-LA level increased in parallel to the subsequent development of organ failure [29, 239]. In other models, Lactate added to CLIF-C ACLF outperformed CLIF-C ACLF and MELD scores to predict early mortality [240]. All scores have been developed and validated in independent data sets and appear to have better diagnostic performance than MELD and MELD-Na as well as ICU-specific scores (e.g., Acute Physiology and Chronic Health Evaluation or Sequential Organ Failure Assessment [SOFA]). The differences between prognostic models are described in Table 8 [2, 75, 86, 91, 150, 241–244].

**Table 8:** Strengths and weaknesses of prognostic models for ACLF

Scoring system (Ref.)	Variables included	Specific to ACLF	Focuses on hepatic, renal and cerebral dysfunction	Applicability to multiple etiologies/regions	Assesses baseline as well as evolving parameters	Ease of use	Guidance for therapy
MELD Na [75]	Bilirubin, Creatinine, PT-INR, Sodium	No	No	Yes	No	Yes	Yes*
CTP score [241]	Albumin, bilirubin, PT-INR, ascites, HE grades	No	No	Yes	No	Yes	Yes*
SOFA [242]	P/F ratio, mechanical ventilation, platelets, GCS, Bilirubin, MAP/ need for inotropes, Creatinine	No	No	Yes	No	No	No
APACHE II [243]	History of severe organ failure, age, temperature, MAP, pH, pulse rate, respiratory rate, sodium, potassium, creatinine, acute renal failure, hematocrit, WBC count, GCS, FiO2	No	No	Yes	No	No	No
AARC score [86]	Bilirubin, HE grades, PT-INR, Lactate, creatinine	Yes	Yes	Yes	Yes	Yes	Yes
CLIF-C ACLF [91]	Age, WBC count, bilirubin, creatinine, HE grades, INR, MAP, P/F ratio	Yes	No	Yes	No	No	Yes
CLIF-C OF/ CLIF-SOFA [2]	Bilirubin, creatinine, HE grades, INR, MAP, P/F ratio	Yes	No	Yes	No	Yes	Yes
TPPM [150]	Bilirubin, PT-INR, HBV-DNA, complications	HBV-ACLF only	No	No	No	Yes	No
COSSH II [244]	PT-INR, HE, Bilrubin, Urea and Neutrophil count	HBV-ACLF only	Yes	No	No	No	Yes

*MELD and CTP scores have been validated for use in the setting of decompensated CLD, and hold some value in ACLF as well

The dynamic assessment of severity scores would capture the response or lack of response to therapy. The value of serial evaluation of scores to determine prognosis and accurately predict those patients who may need further support or in whom further treatment may be futile has been demonstrated in ACLF (ACLF grade, CLIF-C ACLF, and AARC scores) [29, 32, 55]. Sequential assessment is usually performed from 48 h to 3–7 days after diagnosis and initiation of therapy. The best reassessment time point has not been defined but will probably depend on the initial severity of ACLF. Future efforts should focus on improving the accuracy, reliability, and applicability of prognostic models through prospective validation studies, and incorporation of novel biomarkers using machine learning techniques. Whether increasing accuracy in prognostication would translate into better patient outcomes remains to be assessed.

**Recommendations: prognostic models in ACLF**

11.1.1 Prognostic models specific for ACLF predict mortality at days 30 and 90. (LoE: High, Recommendation: Strong)
11.1.2 Severity scores must be assessed on days 0, 3, 7, and 14 to identify individuals requiring urgent liver transplants. (LoE, Moderate, Recommendation: Strong)
11.1.3 An ideal prognostic model for ACLF should accurately predict the outcome, and distinguish organ failures of utility (hepatic, coagulopathy, cerebral) and organ failures of futility (circulatory, respiratory). It should also have broad applicability, adaptability, dynamicity, ease of use, and guide therapy effectively (LoE- Low, Recommendation- Weak)
11.1.4 AARC score is a validated prognostic model for ACLF. It is more accurate compared to MELD/MELD Na., CLIF-SOFA, and SOFA scores for patients with ACLF. (LoE- Moderate, Recommendation- Strong)
11.1.5 The combined application of MELD scoring and ACLF grading can better guide liver transplant allocation for ACLF patients but need more data (LoE- low, Recommendation- low)
11.1.6 The TPPM model may have superior predictive value for HBV ACLF outcomes than MELD and CLIF-SOFA models but needs further validation (LoE- Low, Recommendation- Weak)
11.1.7 There is evolving evidence for the role of machine learning/artificial intelligence-based tools for prognostication in ACLF, but such tools need further validation prior to clinical use (LoE-Low, Recommendation-Weak)

## Liver transplantation (LT) in ACLF

**Fig. 15:**
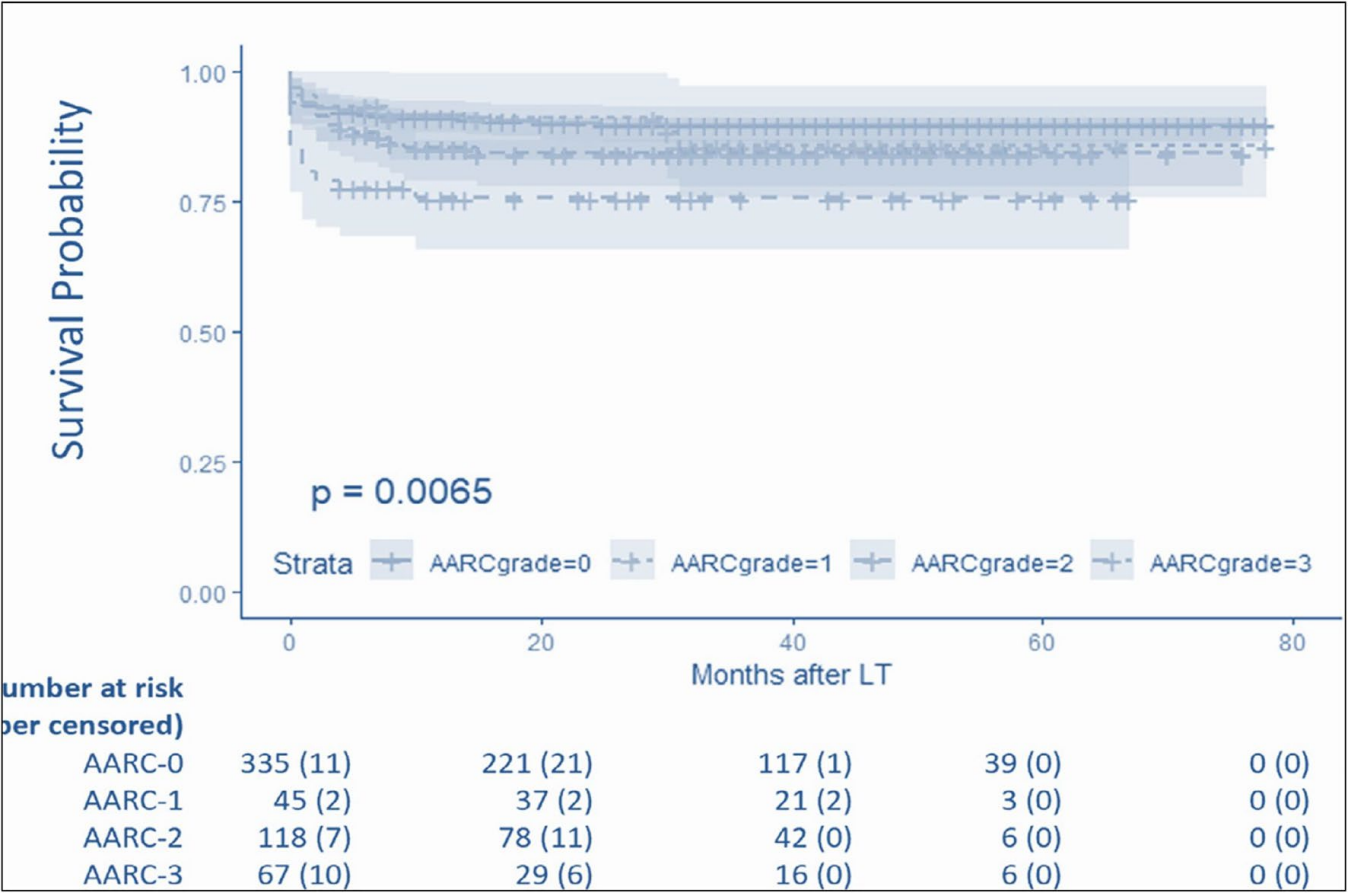
Liver Transplant outcomes of ACLF stratified by AARC score

ACLF is characterized by a rapid downhill course with the onset of extrahepatic organ failure(s) leading to high short- and medium-term mortality (34–51%) [1]. LT is the only potentially curative treatment option with excellent outcomes, irrespective of etiology. The survival in patients with ACLF and non-ACLF undergoing LT is comparable [32, 97, 245, 246]. However, post-transplant survival is dependent on the grade of ACLF [32, 247] (Fig. 15). Quick decisions and appropriate timing of LT in ACLF can be lifesaving. A sick patient who is deemed ineligible for medical management can be listed and prioritized for LT. While all patients should be aggressively managed with nutrition and other etiology-specific appropriate therapies, bridging therapies such as TPE can reverse the syndrome of ACLF in a few.

**Fig. 16:**
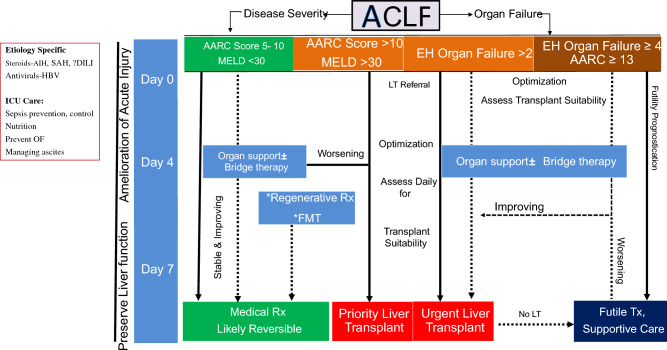
Algorithmic Management of ACLF, based on the use of AARC score in a dynamic fashion and presence or recovery from organ failure

An analysis of 1021 patients from the AARC cohort suggested that a higher proportion of patients would become eligible for LT by the end of the first week (Fig. 13) [152]. At baseline, only 35% would be eligible for LT, which increases to 61% by the end of one week. Survival is excellent, ranging from 80 to 95% in patients with ACLF who undergo LT at 5 years [96, 248]. A serial assessment of these patients in the first week of hospitalization is needed to consider LT as a priority and the AARC score provides a simple dynamic assessment for the selection of patients and the outcomes (Figs. 15 and 16). The ACLF patients are generally sick and often critically ill and admitted to ICUs; with rapid progression of liver failure and risk of multiorgan failure, transplantation is feasible only in a small proportion of patients [249]. The proportion of LTs performed for ACLF varies due to differing definitions and ranges between 10 and 25% [248, 250]. The LT waiting list mortality in ACLF patients is variable, with initial studies reporting up to 80% of patients being delisted for organ failures and sepsis; however, recent studies suggest delisting in only a quarter of patients due to improved medical management [251, 252]. Although timely LT can provide significant survival benefits, healthcare utilization is exponentially high in these patients, both pre-, and post-LT [253, 254].

### Liver transplant: transplant window and prioritization for LT

Liver transplantation is the only definitive therapy for ACLF, and several studies have reported improved survival with timely transplants [32, 97, 152, 246, 254–269] (Table 9).

**Table 9:** Profile and outcome of Liver transplantation for ACLF

S. no	Author name, Country, Definition used, Ref	Total number of transplants/Recipient Characteristics	Grade of ACLF at LT	LT type and donor characteristics	Time to LT or Duration of hospital stay prior to LT	Survival	Comments
1.	Sacleux et al. France. 2024. Canonic. Prospective single-center study [255]	50 of the 96 eligible ACLF patients underwent LT Total 200 admitted Age-55.2y M/F-148/52 Etiology-sepsis and alcohol MELD-32	Grade 0–1-40 Grade 2–50 Grade 3–110	All DDLTs	Time to transplant after listing -10 days (3–54)	~ 35% listed 1y survival 94% post-LT vs. 15% in non-LT ACLF	61% had sepsis at admission Mechanical ventilation and > 2 organ failures at day 3 associated with waitlist mortality (futility)
2.	Hernaez and Karvellas et al. USA and France. Canonic. Multicenter retrospective study [256]	521 ACLF Age-55y M/F-299/222 Etiology-alcohol Trigger-Extrahepatic MELD Na-39.5 Validated in 120 patients	Grade 2–237 Grade 3–284	All DDLTs Age-36y	Time to transplant after listing 7 (3–36) days	1y survival-80%	60% deaths within first 3 months SALT M Score predicts 1y mortality post-LT, ACLF LT LoS score predicts LoS after LT
3.	Alukal et al. USA. Canonic. UNOS database [257]	7607 ACLF 3 listed	All ACLF 3	All DDLTs	3498 underwent LT within 7 days (early LT) and 1308 between 8–28 days (late LT)	1y survival was 86.2%. 87.2% in early LT vs. 83.6% in late LT group	Age, diabetes, respiratory failure, DRI > 1.7, and late LT (> 7 days) associated with high mortality while high albumin reduced mortality
4.	Cronst et al. Brazil. 2023. Canonic. Single center retrospective	25 of 369 transplants were for ACLF Age-53y M/F-11/14 Acute etiology-SBP (44%) Chronic cause-HCV	ACLF-1–9 ACLF-2–8 ACLF-3–8 MELD-32	?DDLT	22 days	30 day survival-80% 1y,3y,5y and 7y survival-76%,60%,54% and 54%	-
5.	Kulkarni et al. India. 2023, APASL. Single center retrospective study [254]	55 of 73 listed underwent LT Age-38 Acute and chronic etiology-alcohol	AARC ACLF grade I-5.5% ACLF II-87.3% ACLF III-7.3% MELD Na-31	All LDLTs Age-34 M/F-31%/69% GRWR-1.1	60 days	1y survival-72.7%	AARC score > 10, CLIF-C ACLF > 44, and CLIF OF > 9 predicted survival benefit with LT
6.	Li and Liang et al. China. 2023. COSSH. Multicenter prospective database analysis [259]	368 underwent LT Age-46y M/F-317/51 HBV-100% MELD-26	Grade 1–33% Grade 2–33.7% Grade 3–33.4%	All DDLTs	Time to transplant-9 days	1y survival-77.2%	COSSH-ACLF II predicts waitlist and post LT mortality
7.	Michard and Artner et al. France. 2022. Canonic. Single center retrospective study [260]	100 ACLF patients Age-54–56 M/F-70/30 Precipitant-infection Etiology-alcohol MELD-	All ACLF 3	All DDLTs	Time from admission to LT-8 days	1y survival 66% from 2007–2015 patients and 86% in later cohort (2015–2019)	Adequate optimization with down staged TAM score associated with improved survival
8.	Kim et al. Korea. 2021, Canonic. Single center retrospective study [261]	76 of 110 ACLF underwent LT Age-51.1y M/F-43/33 MELD-33.6 Etiology-Unknown (58%)	Grade-1–10 Grade-2–34 Grade-3–32	DDLT-59 LDLT- 17	12 (5–20) days 63.2% underwent LT in 2 weeks and rest after 2 weeks	83% survival in LT vs. 17.6% in non-transplanted ACLF	ACLF grade progression prior to LT and MELD scores predicted post-LT mortality. DDLT and LDLT had similar outcomes
9.	Belli et al. Europe. 2021., Canonic Multicenter study ELTR database [269]	234 of 308 ACLF patients underwent LT Age-56y M/F-205/103 Etiology-alcohol	ACLF-1–68 ACLF-2–109 ACLF-3–131	All DDLTs	8 (3–19.5) days (20 days for ACLF-1; 8 days for ACLF-2 5 days for ACLF-3)	24% died on waitlist 84% survival	MDROs infection, arterial lactate and RRT pre-LT predicted mortality post-LT. LT for ACLF 2/3 varied across centers. Highest in France and Belgium and lowest in Spain and UK
10.	Choudhury et al. 2021, APASL [152]	41 LDLT vs. 191 no LDLT Age-41.7y M/F-21/20 MELD Na-27.3		All LDLT	18 (5–90) days	90 day mortality-12.2% in LT vs. 54.5% in no LT group	Eligibility of LT at baseline was 34.6% which increased to 61.3% by end of 1st week
11.	Sundaram et al. USA. 2020, Canonic. UNOS database [91, 264]	25,777 ACLF vs. 31,024 no ACLF Age- M/F-16,342/ 9435 Etiology-HCV MELD-33.6	Grade-1–8757 Grade-2–9039 Grade-3–7981	All DDLTs Age-38.9y M/F-15,505/15519	No ACLF-103 days ACLF-1–48 days ACLF-2–22 days ACLF-3- 12 days	5-year survival ACLF-3–67.7% vs. 75–79% in no ACLF, ACLF-1/2	Infection commonest cause of mortality. High mortality in first year. OFs predicted long term mortality
12.	Sundaram et al. USA. 2020, Canonic. UNOS database [263]	3,636 ACLF underwent LT Age-51.5y M/F-2307/1329 Etiology-alcohol MELD-34.3–41	Grade-0–2-892 Grade-3–2744	DDLTs	< 28 days	1y survival was 82.43% in ACLF-3 vs. 88–90% in ACLF-0–2	Grade improvement in ACLF-3 to 0–2 impacts survival post-LT
13.	Artzner et al. France and UK. 2019, Canonic Multicenter study [265]	152 ACLF-3 patients Age-53y M/F-109/43 Etiology-alcohol MELD-40	ACLF-3–100%	All DDLTs Age-59y	From admission to LT 8 (4–15) days	1y survival 67.1%	TAM score for prediction of post-LT survival
14.	Sundaram and Jalan et al. US. 2019. Canonic. UNOS database [262]	21,269 of 50,552 who underwent LT were for ACLF Age-53y M/F-13,576/ 7693 MELD-32.6	ACLF-1–7375 ACLF-2–7513 ACLF-3–6381	DDLT-98–99% Age-39.2y M/F-12708/ 8561	Time to transplant after listing- 106 days for no ACLF 48 days for ACLF-1, 23 for 2 and 12 for 3	Survival at 1 year:ACLF-1/2–90% ACLF-3–82%	Mechanical ventilation at LT associated with reduced survival post-LT. High mortality among ACLF-3 patients with MELD < 25
15.	O’Leary et al. USA. 2019, NACSELD. Multicenter prospective cohort study [266]	57 of 265 LT were for ACLF MELD-31.1	MELD-ACLF-31.1 No ACLF-27.3	DDLT-94%	Time to LT: 27 days in ACLF vs. 43.5 in no ACLF	1y survival 93% in ACLF and no ACLF groups	Despite higher creatinine and RRT in ACLF group renal recovery was similar among ACLF and no ACLF patients
16.	Thuluvath et al. USA. 2018, Canonic.UNOS database [267]	19,375 transplanted within 30 days Age-54.8y M/F-17,516/ 1859 Etiology-HCV MELD -25.3		DDLTs dominant	No OF-156 days One OF-26 days 2 OF-8 days 3–4 OF-5 days 5–6 OFs-4 days	90% survival in no OF vs. 81% in those with 5/6 OFs	Patients with MOFs died on waitlist. Poor survival in respiratory failure and those requiring life-support systems
17.	Bhatti et al. Pakistan. 2018, Canonic. Single center retrospective study [268]	60 of 119 transplants were for ACLF Age-45y M/F-48/12 Acute etiology-Unknown MELD-29	ACLF-1–43. ACLF-2–15 ACLF-3- 2	All LDLTs Right lobe grafts-96.7%	5.5 days after admission	1y survival- 91%	Survival better with LT. More ACLF-1,2 underwent LT
18.	Arturu et al.France. 2017, Canonic. Multicenter retrospective study [97]	73 of 1,303 transplant were for ACLF-3 Age-56.8y M/F-51/22 Etiology-Alcohol MELD-40 at LT	ACLF-1–133 ACLF-2–181 ACLF-3–73	DDLTs	Time to transplant after listing was 8 (3–24)	Survival at 1 year: No ACLF-90%; ACLF-1–82.3%; ACLF-2–86.2%; ACLF 3–83%.; Non-transplanted controls-8%	Bacterial infections in 80% of ACLF 3 post LT. All patients with ACLF-3 developed complications (100%)
19.	Gustot et al. 2015, Canonic. Prospective multicenter Canonic study [32]	35 of 388 patients with ACLF underwent LT	No ACLF-10 ACLF 1–5 ACLF 2–11 ACLF 3–9	All DDLTs	Time to transplant from diagnosis was 11 (1–28) days	1y survival No ACLF-90% ACLF-1–80% ACLF-2–71.6% ACLF-3–77.8% ~ 50% had resolution of ACLF	Grade of ACLF at day 3–7 determined the outcome Early LT (< 28 days) for patients with ACLF had excellent survival benefit
20.	Finkenstedt et al. Austria. 2013, APASL. Single center retrospective study [246]	33 of 144 ACLF underwent LT Age-57y M/F-95/49 Etiology-alcohol MELD-28		All DDLTs	24 (5–115) days	3 m, 1y, and 5y survival was 94%,87%, and 82% in ACLF compared to 98%,93% and 82% in non-ACLF group	Waitlist mortality 54%. Sepsis, mechanical ventilation, INR, sodium levels reduced survival while LT improved survival

Table modified from Kulkarni, and Reddy et al. with permission

SALT-M: Sundaram ACLF LT mortality score = P(death within 1 year after LT) = 1/[1 + exp(-(-3.412 + 0.366*(Age > 50) + 0.032*BMI + 0.414*one pressor + 1.192*two or more pressors + 0.599*respiratory failure + 0.417*diabetes mellitus))]*100%. SALT M score is dependent on age (50y), BMI, use of vasopressors, respiratory failure and diabetes mellitus and the score ranges between 0–60

ACLF LT LoS score ranges between 0 and 40 and is dependent on age, respiratory failure, BMI, MDRB/fungal infection, death within 90 days of LT, circulatory failure, WBC count, diabetes mellitus, and use of RRT. It can predict the length of stay post-LT

*ACLF* acute-on-chronic liver failure, *BMI* body mass index, *ECD* extended criteria donor, *LT* liver transplantation, *MELD* model for end-stage liver disease, *NA* sodium, *MDRB* multidrug-resistant bacteria, *ICU* intensive care unit, *RRT* renal replacement therapy, *OF* organ failure, *TAM* transplantation for ACLF-3 model, *LDLT* living donor liver transplantation, *SALT-M* Sundaram ACLF LT mortality score, *LoS* length of stay, *WBC* white blood cell count, *RRT* renal replacement therapy

ACLF is characterized by its severity and high heterogeneity, and it involves numerous factors that influence patient prognosis. These factors include patient-related variables such as age, comorbidities, and the extent of liver dysfunction. The course of ACLF assessed 3–7 days after diagnosis was an independent predictor of mortality regardless of initial ACLF grade [32]. Therefore, sequential assessment of disease severity score at these time points may be used to determine prognosis and accurately predict the need for further support, early LT, or a futile attempt. In addition, the prediction of 28-day or 90-day mortality at admission was confounded by regional variation (e.g. 90-day mortality in 41% in North America versus 68% in South Asia) and likely reflective of differences in a patient population at presentation [160]. The “golden window” to transplant these patients is in the first week of illness. For every 1-point rise in AARC score beyond 10, day 7 mortality increases by 20% [152].

### Unsuitability, contra-indications, and futility of LT in ACLF

Despite improved survival in ACLF patients with LT, a dilemma still exists over the prioritization of LT in these specific populations. The criteria indicating unsuitability for liver transplantation in ACLF include the presence of sepsis with two or more organ failures or uncontrolled sepsis, advanced azotemia having serum creatinine more than 4 mg/dl or a threefold rise in creatinine from baseline or the necessity for renal replacement therapy, HE necessitating ventilator support for over 72 h, active gastrointestinal bleeding, irreversible neurological, cardiopulmonary dysfunction, severe ARDS and patients requiring increasing doses of pressors. Patients with ACLF receiving marginal quality donors also do poorly—therefore, older donors, donation after cardiac death (DCD) donors, and steatotic livers should not be used. Patients with previous abdominal surgeries and portal hypertension present additional surgical risks and are less than optimal candidates for liver transplantation. In addition, frail and sarcopenic recipients have poorer outcomes and are not suitable for LT.

The contraindications of LT in ACLF patients encompass a spectrum of comorbidities and complications that may significantly impact post-transplant outcomes and are the same as for other cirrhosis patients. These include active malignancy, advanced hepatocellular carcinoma beyond transplant criteria, uncontrolled systemic infections, and severe extrahepatic comorbidities with limited life expectancy such as advanced heart failure or end-stage respiratory disease, severe malnutrition, and active substance abuse. A recent consensus developed by 35 international experts from North America and Europe has identified specific contraindications, including a PaO_2_/FiO_2_ ratio below 150 mmHg, a noradrenaline dose exceeding 1 µg/kg/min, and or serum lactate levels > 9 [270]. These criteria were, however, for critically ill cirrhosis patients. While liver transplantation represents the sole curative option for ACLF patients at this juncture, it is linked to elevated postoperative complications and prolonged stay in the ICU and hospital, surpassing those associated with other indications.

LT is futile in a patient who has a high probability of early mortality after transplant or has predictably an unacceptable quality of life and or/multiple complications post-LT [271]. A post-LT survival of < 3 months (or in-hospital mortality) is considered as a futile LT [272]. The futility of LT may be considered for patients with 4 or more organ failures or a CLIF-C ACLF score > 64 (at days 3–7) if they have other contraindications for LT [32]. Similarly, an AARC score > 13 indicates the futility of LT. Transplantation for ACLF-3 model (TAM) score, which is based on age (≥ 53 years), arterial lactate (≥ 4 mmol/L), mechanical ventilation with P/F ratio ≤ 200 mmHg and pre-LT leucocyte counts (≤ 10 G/L), can predict one-year survival post-LT [265]. A score > 2 predicted survival of 8% at one year compared to 84% in those with a score of ≤ 2. There are several other scores, such as futility risk score (> 8), clinical frailty score > 6, and SALT-M score > 30, which indicate futility but need to be validated in further studies [256] (Table 10). ACLF patients initially deemed unsuitable or futile for LT should undergo regular reassessment by a multidisciplinary team to evaluate for changes in clinical status, response to medical therapy, or availability of bridging therapies that may improve transplant candidacy. Whilst scoring systems help define timing for LT escalation and features suggesting futility, clinical and ethical challenges remain in the referral and activation of appropriate candidates. The American College of Gastroenterology (ACG) guidelines suggested that patients with cirrhosis and ACLF who continue to require mechanical ventilation because of adult respiratory distress syndrome or brain-related conditions despite optimal therapy should not be listed for LT [273]. LDLT is the better option once indicated in ACLF patients. It is well known that a delay in LT is associated with high healthcare resource utilization and financial burden [254].

**Table 10:** Factors associated with adverse outcomes after liver transplantation for ACLF

*Pre LT factors*	
Pulmonary	Ventilatory status: On ventilator, respiratory failure, ARDS
Hepatic	Lactate levels > 4 mmol/L
Kidney	Serum creatinine > 4mg/dL Increase in creatinine by 300% from baseline Need for renal replacement therapy
Infection	Sepsis or infections with MDRO, Fungal or nosocomial infection Septic shock requiring vasopressors for maintenance of MAP > 65 mmHg Uncontrolled Sepsis with 2 or more organ failures Low leukocytes count prior to transplant
Clinical course	ACLF grade and high MELD CLIF-C SOFA > 64 or Futility risk score > 8 or Clinical frailty score ≥ 7 or SALT M score > 30 or COSSH ACLF II < 7 or > 10 or TAM > 2
	Longer ICU stay before transplant Progression of ACLF 4 or more organ failures
Miscellaneous Critical care concern	HE requiring ventilatory support > 72 h Active GI bleed Active substance Use/multiple failed rehabilitation/Poor psycho-social support
Demographics	Advanced age
Malignancy	Hepatocellular carcinoma
*Transplant -related*	
	Center-related (multidisciplinary teams and expertise)
	High Donor Risk Index
	Intraoperative blood transfusion
*After transplantation*	
	Rejection Episodes
	Sepsis and multi organ failure

*MAP* mean arterial pressure, *HE* hepatic encephalopathy, *CLIF-C SOFA* Chronic Liver failure consortium Sequential organ failure assessment, *SALT-M* Sundaram ACLF Liver transplantation mortality, *COSSH* Chinese Group on the Study of Severe Hepatitis B, *TAM* transplantation for ACLF-3 model

### Prioritization for living and deceased donor liver transplantation

At present, patients with ACLF are not given a higher priority for organ allocation for deceased-donor liver transplantation [1, 273–275]. The ethical principles involved in organ allocation for transplantation are equity and utility. The expected 1-year and 5-year survival post liver transplantation are now 93% and 75%, respectively. ACLF patients should be selected for liver transplantation only if they are likely to achieve these levels of survival. It is unclear whether these survival outcomes are applicable to deceased-donor transplantation.

Various prognostic models/scores developed for ACLF are derived from the cohorts used to define ACLF [5, 86, 91, 238, 244]. These scores are used for prognostication of disease and prioritization of liver transplants. The allocation of organs in deceased donor liver transplant settings is based on the MELD scoring system, and MELD-based allocation is prone to significant disparity across all three major definitions of ACLF (APASL, EASL-CLIF, and NASCSELD) [237]. The 90-day mortality is significantly higher in patients with ACLF than the expected mortality based on the MELD Na scores. Patients with ACLF and lower MELD scores have higher waitlist mortality, probably due to a lack of capturing the extrahepatic organ failure scores [262, 276]. Furthermore, patients with ACLF may not benefit from the “Share 35” rule as most patients have MELD scores between 30 and 35 [237, 277]. Nevertheless, Share 35 has significantly increased the number of transplants done for ACLF and MELD > 35 due to regional sharing [278, 279]. There are no studies evaluating the role of these ACLF scores derived with respect to organ allocation, which needs to be assessed. Nevertheless, dynamic scores on days 3 and 7 predict the need for LT [86, 254].

Due to the lack of structured national/regional DDLT programs in Asian Countries, LDLT gives patients a ray of hope and, indeed, is the most frequent type of liver transplant conducted in Asia. With a focus on the Asian cohort, Choudhury et al. have proposed a concept of “ineligibility for LT and Liver Transplant Window” based on the analysis of a large AARC database. There are four basic principles of this concept: (i) As patients with ACLF have an unpredictable course and high mortality, nearly all should be evaluated for LT at the presentation, (ii) in the presence of ACLF transplant outcome may not be optimal, so resource utilization is a particular concern, (iii) optimizing patients with ACLF over one week, with close monitoring can provide differentiation into the best candidates for transplant or for futility, and (iv) high-grade ACLF should not be viewed as a contraindication, but at a certain time point the patient may be ineligible or unsuitable [280].

The benefits of LDLT in patients with ACLF can provide outcomes that are comparable to those of DDLT in cases of ACLF [248, 254, 281, 282]. However, the optimal criteria for LDLT need further evaluation. The benefit of LDLT in patients with ACLF is its ability to provide rapid transplantation to critically ill patients but LDLT can potentially have serious consequences for both the donor and the recipient.

There is a consensus across all societies that patients with ACLF grades 1 and 2 should be listed for LT. According to APASL, a baseline MELD score > 28, AARC Score > 8, and advanced HE in the absence of overt sepsis or multiorgan failure can be considered for early LT. Progressive and severe ACLF should be prioritized like Acute Liver Failure (ALF) as both are dynamic syndrome with SIRS as the driving factor. The clinical course of the syndrome and the likelihood of recovery without LT should be assessed on a regular basis and should determine the need for LT when the organ is available. United Kingdom has adopted a pilot program in 2021 with a new tier system for LT in ACLF (ACLFLT) just behind supra-urgent listing [87]. The program is designed to facilitate LT in sicker ACLF -2 and ACLF -3 patients and thereby reduce waitlist mortality.

### Right time for transplant in alcohol-related ACLF

Alcohol misuse is a common precipitating factor for ACLF. Approximately 50% of patients with recent alcohol abuse present as ACLF and such patients respond poorly to medical management including corticosteroids [149, 283]. Early LT (both LDLT and DDLT) is an excellent treatment for these patients with AAH without a pre-defined period of abstinence [284, 285]. LT is reserved for patients who have excellent psychosocial profiles and support. A double equipoise exists in ACLF where the disease and organ failure drive survival, which may lead to prioritizing those with poor psychosocial support (sick quitters) for LT. Although a significant proportion of patients can improve with alcohol abstinence, those with ACLF may not recover despite abstinence. While the futility markers (as discussed above) remain the same in alcohol-related ACLF, those with controlled sepsis, evidence of portal hypertension (large varices, variceal bleeding, gross ascites, HRS [recovered]), and advanced liver failure (small cirrhotic liver, high bilirubin and INR) should be prioritized for LT even in the absence of strict alcohol abstinence period. Factors that can influence drinking relapse are duration of drinking, continuous drinking after diagnosis of liver disease, young age, substance abuse, legal consequences of drinking, poor social support, and previous failed attempts [286]. However, the duration of abstinence before LT has not been consistently shown to predict alcohol relapse post-LT and therefore these patients with alcohol-related ACLF should undergo early LT in the absence of other contraindications.

### Innovations in technique and management for improved transplant outcomes in ACLF

The donor selection is the crucial step in LDLT. A minimum of 0.8 GRWR is suggested with age between 18 and 50 years and minimal (up to 15%) or no steatosis. Orchestrating retrieval of the graft with an explant of the cirrhotic liver in LDLT enables reduction of cold ischemia time, which, along with short warm ischemia times, reduces ischemia–reperfusion injury. Attention must be paid to minimize blood loss during hepatectomy. Expedient hepatectomy with portal and caval clamping reduces bleeding and hence transfusion requirement. Creating a portacaval shunt for sick recipients is also helpful. Complete caval cross-clamping is poorly tolerated in these sick recipients, and anastomosis can be achieved with partial cross-clamping. The biliary anastomosis should preferably be duct to duct as enteric anastomosis may introduce subsequent infective episodes or leaks. A delayed biliary reconstruction 24–48 h later can be considered in these patients once the bowel edema settles. This provides an additional opportunity for peritoneal lavage as intraabdominal infections and collections are common.

Intraoperatively, restrictive, or goal-directed fluid therapy is beneficial over liberal fluid therapy. During the dissection and anhepatic phases, the replacement of blood loss with packed red cell units and factor concentrates guided by point-of-care viscoelastic testing ensures a dry surgical field. Reserving correction of acidosis to prior to graft reperfusion prevents inadvertent fluctuations in sodium levels. Shortening anhepatic time, creating of porto-caval shunt and use a piggyback technique for implantation avoiding a complete caval clamping are important surgical innovations. The role of intraoperative continuous renal replacement therapy (iCRRT) in preventing hemodynamic alterations and managing electrolyte and acid–base disturbances needs to be evaluated in patients with ACLF [287]. In patients needing post-operative renal support, in the presence of hemodynamic stability, conversion of CRRT to intermittent SLED allows earlier mobilization.

### Immune-suppression post-LT for ACLF

Triple drug immunosuppression (steroids, calcineurin inhibitors and anti-metabolites) are commonly used in the post-transplant period and are reduced to dual or monotherapy once the patient’s liver function has adequately recovered. Patients with ACLF frequently have renal dysfunction and require a renal-sparing regimen. The use of low-dose tacrolimus or delayed introduction of CNIs, the use of anti-IL2 receptor antibodies, and the introduction of early mycophenolic acid can prevent renal dysfunction [288, 289]. Furthermore, maintaining tacrolimus trough levels between 4 and 8 mg/mL during the first month with intra-patient variability < 40%, use of basiliximab, and early introduction of mycophenolate mofetil (day 1) can prolong the survival in patients with ACLF [290]. Early everolimus introduction was neither associated with higher rejection rates nor with more surgical complications [4]. Rituximab is not an ideal drug in the pre-transplant period for patients with ACLF due to high MELD in patients planned for ABOi transplants. Such patients may benefit from plasmapheresis to decrease the antibody titers [5]. The long-term outcomes of ACLF post-LT are similar to non-ACLF patients concerning metabolic complications [2].

12. **Recommendations: Liver Transplant in ACLF**
  12.1. Liver Transplant: Prediction of the need, the Transplant window and patient optimization
    12.1.1. Liver transplantation is the only definitive therapy for grade II and III ACLF and such patients should be transplanted early. (LoE: High; Recommendation: Strong)
    12.1.2. Transplant benefit in ACLF should be assessed based on the severity of the acute liver injury, the reversibility of underlying liver disease, and the prognosis of associated extrahepatic organ failure (LoE: moderate; Recommendation: Strong).
    12.1.3. AARC score of 8 (Grade 2) is associated with a non-transplant mortality risk of 20% at 4 weeks, which can be considered as a minimal AARC score cut-off to ensure the benefit of early LT (LoE: Moderate; Recommendation: Weak).
    12.1.4. In LDLT dominant programs, counseling and live donor evaluation should be started early after the initial assessment to avoid delays (LoE: Low; Recommendation: Strong).
    12.1.5. Liver transplantation for ACLF provides outcomes similar to LT for non-ACLF but with a more challenging peri-operative course (LoE: Moderate; Recommendation: Strong).
  12.2. Unsuitability, Contra-indications, and futility of LT in ACLF
    12.2.1. Uncontrolled sepsis with or without 2 or more organ failures, advanced azotemia or increase in creatinine by 300% from baseline or the need for renal replacement therapy, PaO_2_/FiO_2_ ratio below 150 mmHg, a noradrenaline dose exceeding 1 µg/kg/min, and or serum lactate levels > 4 mol/l are considered as conditions for temporary unsuitability to proceed for LT (LoE: Moderate; Recommendation: Strong).
    12.2.2. Contraindications for liver transplant in ACLF are not different from the general contraindications for liver transplant (LoE: High; Recommendation: Strong).
    12.2.3. AARC > 13, COSSH ACLF II score of < 7 or > 10, CLIF-C > 64, TAM score > 1 indicate futility of LT in ACLF. (LoE: Moderate; Recommendation: Strong).
  12.3. Prioritization for living and deceased donor liver transplantation
    12.3.1. All patients with ACLF should be evaluated for liver transplantation in the absence of contraindications. (LoE-Moderate, Recommendation-Strong).
    12.3.2. Prioritization for organ allocation for liver transplantation in patients with ACLF should be based on prevailing scoring systems till such time a scoring system for ACLF is created that includes transplant benefit. (LoE-Moderate, Recommendation-Strong).
    12.3.3. Transplant outcome in LDLT and DDLT are comparable at 1 year (LoE: Moderate Recommendation: Strong)
    12.3.4. All ACLF patients should be considered for LT at presentation irrespective of LDLT and DDLT scenario as decided by the existing disease severity score, organ failure(s), and general condition (LoE: Low; Recommendation: Weak)
    12.3.5. Priority for severe ACLF in the DDLT setting needs to be considered as survival in Grade 3 ACLF is lower as compared to Grades 1 and 2. (LoE -Moderate. Recommendation-low)
    12.3.6. Currently available prognostic models of ACLF cannot aid in predicting post-transplant survival. (LoE: Low, Recommendation: Weak)
  12.4. Right time for transplant in alcohol-related ACLF
    12.4.1. Duration of abstinence should not be a criteria to select patients for LT in patients with alcohol-related ACLF (LoE: Moderate; Recommendation: Strong)
    12.4.2. Liver transplant should be considered in those patients who are non-responders to steroid therapy or are not the candidates for steroids because of contraindications (LoE: Moderate; Recommendation: Strong).
    12.4.3. Patients should have demonstrable psychological stability following a thorough psychosocial assessment before consideration for liver transplant (LoE: High; Recommendation: Strong).
  12.5. Innovations in technique and management for improved transplant outcomes in ACLF
    12.5.1. Donor selection and ideal GRWR in LDLT as well as graft steatosis are crucial for a better outcome of LT in ACLF (LoE: Low; Recommendation: Weak)
    12.5.2. To reduce the occurrence of sepsis, a consideration can be made to lower the dose of corticosteroid given at reperfusion. (LoE: Low; Recommendation: Weak)
    12.5.3. Intraoperatively restrictive or goal-directed fluid therapy is beneficial over liberal fluid therapy. (LoE: Low; Recommendation: Weak)
    12.5.4. Intraoperative CRRT may be used in patients with renal injury or those with metabolic complications (LoE: Low; Recommendation: Weak)
  12.6. Immune-suppression in ACLF patients Post-transplant
    12.6.1. In patients transplanted for ACLF and renal dysfunction, a CNI-sparing strategy, and early introduction of MMF and Everolimus after ruling out surgical complications predicts greater survival without increased risk of rejection. (LoE; Moderate, Recommendation: Strong)
    12.6.2. ABO-incompatible liver transplant in ACLF patients carries a higher risk and should be avoided. (LoE; Moderate, Recommendation: Strong)
    12.6.3. Long-term post-transplant complications of immunosuppressive medications are the same in ACLF and non-ACLF patients. (LoE; Moderate, Recommendation: Strong)

## Pediatric ACLF

### Definition

The 2019 AARC ACLF guidelines pioneered an attempt to define pediatric ACLF as “an acute hepatic insult manifesting as jaundice (total bilirubin ≥ 5 mg/dl) and coagulopathy (INR ≥ 1.5) complicated within 4 weeks by *clinical/ radiological* ascites or *HE* in a child with previously *known* or unknown underlying CLD” [1]. The pediatric modifications were (i) inclusion of radiological ascites as detection of clinical ascites could be unreliable particularly in younger children and (ii) recommending the use of a modified HE assessment scale in children up to 3 years of age [291]. Alternatively, pediatric studies from Europe and the US have utilized the EASL or NACSELD definitions with subtle modifications to describe pediatric ACLF in these settings [292, 293]. The rapid liver dysfunction present in several metabolic disorders without a concomitant significant rise in bilirubin suggests that bilirubin may be less important to define ACLF in a subset of pediatric patients. The experts unanimously concurred that a universal global definition is needed to prioritize this very sick group of patients for LT. In addition, the experts advocated collaborative data collection and analysis to broaden the pediatric ACLF definition amalgamating the key points from the three currently used definitions and lists the various studies of Pediatric ACLF using the various definitions described in the literature.

### Epidemiology

The divergent pediatric ACLF definitions make it difficult to accurately ascertain the burden of pediatric ACLF. The incidence of ACLF is lower in children than adults, varying from 13.4 to 25.7% [56, 294–296]. This may be attributable to (i) higher proportion of treatable etiologies of underlying CLD like Wilson disease (WD) and autoimmune hepatitis (AIH), (ii) lower incidence of co-morbidities, (iii) greater hepatic reserve, (iv) underreporting of pediatric ACLF due to lack of awareness in some parts of the world, and (v) limitations of the current pediatric ACLF definitions [297]. The etiologies of underlying cirrhosis in pediatric ACLF include WD (27.8–46.5%), AIH (22.2–63.6%), and BA (48.1–65%) (Table 11) [56, 292, 294–296, 298–302]. ACLF is often the first presentation of the underlying CLD, especially WD and AIH (75–86% of cases in Asia). In contrast, reports of ACLF in children with BA mostly come from the West using EASL/ NACSELD definition [292, 293, 298]. Children with BA and an unsuccessful outcome after Kasai hepatoportoenterostomy may never resolve their neonatal jaundice and often have ascites by 6–12 months of age, precluding them from meeting the AARC pediatric ACLF definition [297]. Nevertheless, it is important to both recognize and define ACLF in BA, which represents the most common etiology for CLD and pediatric LT. A sudden increase in jaundice and ascites in the presence of an obvious trigger in biliary atresia needs to be given due diligence to enable inclusion of this important group in the definition of ACLF.

**Table 11:** Summary of the various studies describing the characteristics and outcome of pediatric acute-on-chronic liver failure

Study, Country, Year	Sample Size	Prevalence	Underlying chronic liver diseases	Acute precipitating event	Extrahepatic Organ Failures	Outcome/ Time point
*Studies defining pediatric ACLF based on APASL definition*						
Alam et al., India, 2016 [294]	86	86/ 640 (13.4%)	Wilson Disease: 46.5% Autoimmune Hepatitis: 34.9% Chronic Hepatitis B: 5.8% Biliary Atresia: 3.5%	Viral hepatitis (Hepatitis A/E): 34.5% Flare (Wilson): 26.8% Flare (autoimmune): 21.4%	Kidneys: 22.6% Respiratory: 16.1% Circulatory: 16.1% 2 or more OF: 21.4%	NLS: 66.7% Death: 25% LT: 8.3% Day 90
Lal et al., India, 2018 [296]						
Lal et al., India, 2018[299]						
Lal et al., India, 2011[56]	31		Autoimmune Hepatitis: 41.9% Wilson Disease: 41.9% Hepatitis B reactivation: 6.5% Secondary Biliary Cirrhosis: 6.5% Indian Childhood Cirrhosis: 3.2%	Hepatitis A: 41.9% Hepatitis E: 9.7% Flare (autoimmune): 9.7% Hepatotoxic drugs: 6.5% Indeterminate: 22.6%		NLS: 80.6% Death: 19.4% Day 90
Sharma et al., India, 2020[295]	31	36/140 (25.7%)	Wilson Disease: 45.2% Autoimmune Hepatitis: 13% Indian Childhood cirrhosis: 9.6% Chronic cholestatic liver disease: 12.8%	Hepatitis A: 25.8% Hepatitis E: 12.9% Parvovirus B19: 16.1% Hepatotoxic drugs: 9.7% Indeterminate: 9.7%	Hematological: 83.9% Neurological: 58.1% Respiratory: 38.7% Circulatory: 25.8% Kidney: 6.5%	NLS: 45.2% Death: 54.8% Day 60
Islek et al., Turkey, 2021[301]	29		Autoimmune Hepatitis: 51.7% Wilson Disease: 27.6% Chronic hepatitis B: 6.9% PFIC3: 6.9%	Flare (autoimmune) including non-adherence: 48.3% Flare (Wilson) including non-adherence: 27.6% Viral Hepatitis: 13.8%		NLS: 75.9% LT: 24.1% Death: 0
Jagadisan et al., India, 2012[300]	17	17/36 (47.2%) of CLD presenting with an acute insult	Cryptogenic: 30.6% Wilson Disease: 27.8% Autoimmune Hepatitis 22.2% Primary sclerosing cholangitis: 8.3%	Hepatitis E: 75% Hepatitis A: 27.8% Hepatitis B: 5.6%	Bacterial sepsis: 25%	NLS: 29.4% Death: 58.8% Lost to follow-up: 11.8%
Studies defining pediatric ACLF based on organ failures (modified EASL/ NACSELD definitions)						
D’Souza et al., UK, 2019[292]	20	20/ 99 (20%)	Study only included children with biliary atresia	Sepsis Gastrointestinal Bleed	Respiratory: 50% Neurological: 40% Kidneys: 20%	LT: 65% Death: 20% High post LT mortality (35%)
Banc-Husu et al., US, 2020[298]	20	20/166 (12%)	Biliary Atresia: 55% PFIC/ Alagille: 15% Cryptogenic: 15%	Gastrointestinal bleed: 30% Cholangitis: 4% Other infections: 4%	Respiratory: 44% Cardiovascular: 67% Neurological: 33%	LT: 57% Death: 22% NLS: 21%
Godfrey E et al., US, 2021 (UNOS database)[293]	264	264/ 10,172 (2.6%)	Biliary Atresia: 48.1% TPN associated liver disease: 13.2% Genetic disease: 11%			Death: 46.6% Higher waitlist mortality than non-ACLF status 1B
Mataya et al., US, 2022[310]	478	478/1286 (37%) of all status 1B listing	Included children with Status 1B with CLD and PELD/ MELD > 25 Cholestatic liver disease: 74% Metabolic liver disease: 8%		Kidneys: 31% Cardio-respiratory: 34% Neurological: 13%	LT: 79% Death on waitlist: 21% Mortality comparable to PALF and higher than non-ACLF 1B
Banc-Husu et al., US, 2023 [312]	351	351/1044 (33.6%)	Included children with biliary atresia only	Cholangitis: 19% Gastrointestinal bleed: 15% Spontaneous Bacterial Peritonitis: 2%	Cardiovascular: 65% Neurological: 31% Respiratory: 25% Kidney: 3%	Post LT mortality comparable between those with ACLF and other status 1B
Studies based on other definitions (PALF study group definition for liver failure PLUS evidence of chronicity)						
Di Giorgio et al., Italy, 2019 [302]	22		Autoimmune hepatitis: 63.6% Wilson Disease: 27.3% Metabolic disorders: 9.1%			LT: 36% Death: 5% NLS: 59%

Abbreviations: *PALF* Pediatric Acute Liver Failure, *PFIC* Progressive Familial Intrahepatic Cholestasis, *TPN* Total Parenteral Nutrition;

Hepatotropic viral infections (hepatitis A, B and E) trigger 35–92% of pediatric ACLF [294, 296, 299]. while non-hepatotropic viruses like Epstein–Barr virus (EBV), cytomegalovirus (CMV), parvovirus B19, influenza, dengue, and coronavirus (SARS-CoV2) can infrequently precipitate ACLF [45, 56]. DILI accounts for 6–10% of acute events in pediatric ACLF. Instances where a viral or drug trigger or other acute precipitant of ACLF cannot be identified are commonly referred to as “flares of the underlying CLD” in WD and AIH [56, 294, 296]. Due to the lack of homogeneity in terminology as well as the extent of diagnostic evaluation, an acute event may be labeled as ‘indeterminate’ when a trigger is not identified in the diagnostic evaluation. Non-hepatic insults like bacterial infections and gastrointestinal bleeding are currently not identified as ACLF precipitants by AARC. However, sepsis (viral or bacterial), gastrointestinal bleeding, and cholangitis are common precipitants of ACLF in children with BA. Children with ACLF usually have lower mortality than adults owing to a lower incidence of multi-organ failure, higher proportion of treatable etiologies (WD, AIH), lower prevalence of comorbidities, and greater hepatic reserves [294, 297]. Table 12 lists the common differences in characteristics of pediatric and adult ACLF.

**Table 12:** Differences in the characteristics of acute-on-chronic liver failure (ACLF) in children and adults

	ACLF in adults	ACLF in children
Definition (APASL)	Only clinical ascites included HE assessment as per West Haven scale	Radiological and/or clinical ascites included HE assessment could be done using modified HE assessment scale in younger children
Epidemiology	Frequent, may affect up to 40% of CLD	Affects around 12 – 25% of those with underlying CLD
Etiologies of underlying cirrhosis	Alcoholic liver disease Chronic viral hepatitis (Hepatitis B and C)	Wilson disease and autoimmune hepatitis (AARC definition) Biliary atresia (EASL definition) ACLF rare in hepatitis B and C
Acute precipitants of ACLF	Acute alcoholic hepatitis Hepatotropic viruses (Hepatitis B most common) Drug-induced liver injury Flare of autoimmune hepatitis Acute portal vein or hepatic vein thrombosis Indeterminate (5 – 15%)	Hepatotropic viruses (hepatitis A most common) Drug-induced liver injury Flare of autoimmune hepatitis Acute Wilsonian crisis No acute event recognizable in about 1/4th of cases
Organ failures	Organ failures more common; Co-morbidities more frequent	OOrgan failures more common
Outcomes	Mortality: 33 – 51%	Mortality lower in pediatric ACLF studies based on AARC definition: 19.4 – 59%

Abbreviations: *ACLF* Acute on chronic liver failure, *APASL* Asia Pacific Association for the study of Liver, *AARC* APASL ACLF Research Consortium, *EASL* European Association for the Study of Liver, *HE* hepatic encephalopathy

### Infections in pediatric ACLF and impact on outcome

Bacterial infection (BI) can be an ACLF precipitant (20–48%) in the EASL and NACSELD cohorts, as well as a complication in the AARC ACLF cohorts [297, 303–305]. Common sites of infection include SBP, pneumonia, skin infections, and urinary tract infections (UTI). Adults with BI are more likely to progress to advanced stages of ACLF and have lower 90-day survival (49% vs 72.5%) than ACLF without BI [303, 305]. Timely initiation of appropriate empiric antibiotic treatment significantly improves both 28 and 90-day survival. Published literature on infections in pediatric ACLF, choice, and efficacy of antibiotics impact on outcome are scarce. A cause of concern is the increasing rates of multi-drug resistant (MDR) BI being reported in children with decompensated CLD [306].

### Management of pediatric ACLF

Treatment of ACLF is similar to adults as described above.

#### Therapeutic plasma exchange in pediatric ACLF

All extracorporeal liver support systems (ELSS) work by removing water and/or albumin-bound toxins and creating a regenerative environment for the liver. TPE prevents cellular damage from “cytokine storm” during SIRS in ACLF. TPE is particularly beneficial in pediatric ACLF caused by WD, which accounts for a major proportion of these cases. A literature search of case reports on the role of TPE showed that 17/37 (46%) of the waitlisted WD patients with NWI ≥ 11 recovered without transplantation [307]. Two recent studies report TFS rates of 47% in 19 cases from India and 81% in 11 cases of WD from China [307, 308]. Importantly, the survival benefit of TPE was evident in the subgroup of patients with early (grade 1–2) HE [309].

### Outcomes and prognostication in pediatric ACLF

Pediatric ACLF is associated with higher short-term (90-day) mortality than children with compensated CLD but may be reversible with early intervention. Children with ACLF listed as status 1B in the United Network for Organ Registry (UNOS) database had higher rates of multi-organ failure, lower LT rate, higher waitlist mortality, and longer waitlist time compared to non-ACLF children with status 1B listing [310]. Several prognostic models have been evaluated in children with ACLF, of which CLIF-SOFA and AARC-ACLF scores have found the maximum clinical application [299]. Several attempts at pediatric modification of these scores have been undertaken as the original scores use absolute serum creatinine which is significantly lower at baseline in children, while the percentage rise in creatinine gives a more accurate assessment of renal dysfunction. Although these pediatric modifications did not improve the sensitivity or specificity of these models to determine the risk of mortality, it is clinically sound to use these scores with their pediatric modifications. While the AARC-ACLF model may be more suitable for prognostication in pediatric ACLF identified using the AARC definition, the CLIF-SOFA score may be better suited for prognostication in the cohort of children identified using the EASL definition. A score of 11 or above in either CLIF-SOFA or AARC-ACLF predicts a risk of death or LT with 84% sensitivity and 90% specificity [299, 304]. The New Wilson Index (NWI) and AARC-ACLF scores have also been found to be effective in the prognostication of children with WD [311, 312]. Daily dynamic monitoring of these prognostic scores may guide overall management, including the need and timing of LT.

### Liver transplantation for pediatric ACLF

Pediatric ACLF is associated with high waitlist mortality both in Asia (30–59%) and the West (22–46%). Allocation of organs and prioritization of critically ill children with end-stage cirrhotic liver disease has been hampered by the lack of an established uniform definition of pediatric ACLF. However, it is clear that pre-LT mortality in children with ACLF (especially those defined as per EASL/ NACSELD) is high, and can be reduced by prioritization of organ allocation in respective national registries. All eligible patients must be listed in national and/or state-specific organ registry, wherever available, and the children should be managed at the well-equipped pediatric transplant center. Timing the LT is crucial as hastening the process before optimization of the host may lead to suboptimal outcomes, and at the same time, unnecessary delay in LT may risk further worsening of multi-organ dysfunction, which may negate LT candidacy, leading to mortality. Post-LT survival and outcomes are comparable in these children to the non-ACLF pediatric LT recipients [312]. Timing of LT is challenging, especially in patients treated with bridging therapies like TPE or albumin-assisted liver support systems. LT is the definitive treatment for children unresponsive to medical and bridge therapies with concurrent deterioration of prognostic scores (AARC-ACLF/CLIF-SOFA ≥ 11). In living donor LT setting, donor evaluation should be initiated early while the response to medical and bridging therapies is being evaluated. Patients should be evaluated daily, and consideration should be given to predictors of post-LT mortality and the potential for reversibility of extrahepatic organ failure before deciding whether to proceed with LT or palliative care.

### Recompensation in pediatric ACLF

There is a recent emphasis on hepatic recompensation, a term used to describe disease regression after an effective treatment or successful elimination of the cause of underlying liver cirrhosis. Standardized criteria for the definition of hepatic recompensation were introduced in 2021 at the Baveno VII consensus meeting [138]. Although the data on recompensation in pediatric ACLF is lacking, the experts agreed that the incidence of recompensation is likely to be higher in children due to a significant proportion of etiologies having specific therapies like WD, AIH, and chronic viral hepatitis.

13. **Recommendations**
  13.1. In the absence of a universally accepted definition, the current pediatric modifications of AARC, EASL, and NACSELD definitions should be used for the diagnosis of ACLF while all attempts should be made towards creating a global consensus definition. (LoE- High; Recommendation- Strong).
  13.2. Attempts should be made to rapidly diagnose and treat Wilson disease and autoimmune hepatitis which are the commonest causes of underlying CLD in the Asia–Pacific region. Biliary atresia is the most common etiology of cirrhosis in pediatric ACLF described in Europe and the USA. (LoE- High; Recommendation-Strong).
  13.3 All cases of pediatric ACLF should be screened for hepatotropic viral infections and hepatotoxic drugs as a precipitating factor. In the absence of these precipitants, children should be screened for non-hepatotropic viral infections**.** ACLF in children with biliary atresia is most often triggered by infections and GI bleeding. (LoE- High; Recommendation-Strong).
  13.4 An appropriate antibiotic stewardship policy needs to be implemented at all levels of medical care given the rising concerns of multi-drug-resistant organisms. (LoE- High; Recommendation- Strong).
  13.5 Therapeutic plasma exchange should be considered in Wilson disease presenting as pediatric ACLF with grade 1–2 HE, as it has been shown to improve native liver survival**.** (LoE- High; Recommendation- Weak).
  13.6 The role of TPE in other causes of pediatric ACLF is not well established and requires clinical decision-making by the treating team. (LoE- Moderate; Recommendation- Weak)
  13.7 Children with ACLF should be treated at centers with multi-disciplinary teams equipped to manage a critically ill child and with liver transplantation programs. (LoE- High, Recommendation- Strong).
  13.8 Pediatric modifications of either the AARC-ACLF or CLIF-SOFA score may be used serially to assess patient prognosis and inform the decision for LT. (LoE- High; Recommendation-Strong).
  13.9 All children with ACLF and organ failure (irrespective of the number of organs involved) should undergo rapid evaluation for LT since post-transplant survival is high and comparable to those without ACLF. (LoE- High; Recommendation- Weak).
  13.10 Recompensation in pediatric ACLF remains unexplored. The incidence is likely to be higher than in adults due to more prevalent treatable etiologies like Wilson disease, autoimmune liver disease, etc. (LoE- Moderate; Recommendation- Weak).

## Global convergence in ACLF

ACLF in the East and West has been defined using different cohorts of patients because of which, the ACLF diagnosis remains a contentious issue. Variations in diagnostic criteria among different guidelines have sparked debates, with significant discrepancies observed in defining severe liver dysfunction. APASL considers patients with chronic liver disease with or without cirrhosis, no prior episode of decompensation, who have severe liver dysfunction, and with acute hepatic insult and extrahepatic complication as a consequence rather than the cause like bacterial infection, GI bleeding, etc. The EASL-CLIF Consortium defines ACLF as the failure of one or more extrahepatic organs in a patient of cirrhosis who was admitted non-electively and often had acute decompensation, while the NACSELD requires two or more extrahepatic organ failures to be present to diagnose ACLF. These definitions are in a continuum with patients developing APASL ACLF (liver failure), then progressing to organ involvement (EASL-CLIF), and then lastly succumbing to multiorgan failures (NACSELD). The Japanese, Chinese, and Mexican hepatology societies define ACLF similarly to the APASL definition. Recently, the AASLD consensus statement defines ACLF as an acute onset condition with rapid deterioration in clinical condition with the presence of liver failure defined by elevated bilirubin and elevated INR in patients with chronic liver disease with or without cirrhosis, and the presence of at least one extrahepatic (neurologic, circulatory, respiratory, or renal) organ failure.

14. **Research agenda**.
  14.1 Timeline and sequence of reversal of ACLF
  14.2 Natural history of recompensated cirrhosis and impact on ACLF
  14.3 Relevance of pre-ACLF in various definitions or inclusion as part of a spectrum.
  14.4 Natural history and outcomes of ACLF and AD in patients with different etiologies.
  14.5 Role of portal hemodynamics in the development of organ failure and correlation of non-invasive tests with HVPG in ACLF patients
  14.6 Risks and benefits of albumin therapy in ACLF and need for replacement versus albumin exchange.
  14.7 Choice of diuretics based on PRA, modest volume paracentesis, and prevention and management of AKI in ACLF
  14.8 Safety and efficacy of non-selective beta-blockers in ACLF and utility of combining with midodrine and other vasoactive agents.
  14.9 The role of TIPS as a bridging modality in intractable ascites and variceal bleeding, in patients with ACLF.
  14.10 ACLF reversal, predictors, and outcome across all definitions.
  14.11 The role of NAC in preventing ACLF due to drug hepatotoxicity
  14.12 Nutritional needs, use as therapy and improvement in survival.
  14.13 Non-transplant novel therapeutic measures, bridge therapies, especially bio-filters and bio-artificial liver devices
  14.14 Role of current prognostic scores of ACLF in LT listing and prioritization in LDLT and DDLT settings and predicting post-transplant survival.

## Way forward for convergence

Consensuses need to emphasize, the need for harmonizing the existing ACLF diagnostic criteria globally for uniform and accurate patient identification (Fig. 7). Different definitions have common and overlapping grounds and highlight the high short-term mortality and the need for urgent LT. The reason for the differences in the definition is the population cohort included in each region is different from others, and the definitions remain relevant to that cohort. As proposed in this consensus, we suggest classifying the ACLF as types A and B, which could include all the definitions. A group of European investigators considers that this document mainly reports the Asian perspectives on the decompensation of cirrhosis and ACLF. These European investigators, although not in agreement with many of the observations and definitions reported in this document, strongly support the attempt to harmonize the existing diagnostic criteria of ACLF. In this perspective, they recommend planning a large international prospective longitudinal study with this specific aim. The protocol of such a study should follow the international recommendations for observational studies, including at least, clear prespecified definitions for organ system failures, data collected on an electronic CRF which has been properly designed, and continuous monitoring by skilled data managers. In addition, this kind of study should involve centers that have expertise in clinical studies.

The first step for harmonizing the definitions and management has been taken in the form of the ‘Kyoto Consensus’ on ACLF. Future accommodative and scientific initiatives could see a universally accepted definition of ACLF and pave the way for new treatment options for better outcomes for this lot of very sick patients.

## Supplementary Information

Below is the link to the electronic supplementary material.

Supplementary file1 (DOCX 358 KB)

Supplementary file2 (DOC 88 KB)

## Data Availability

NA.

## Abbreviations

ACLF: Acute-on-chronic liver failure
AD: Acute decompensation
AIH: Autoimmune hepatitis
AKI: Acute kidney injury
ALD: Alcohol-related liver disease
AVB: Acute variceal bleed
BCS: Budd–Chiari syndrome
CAM: Complementary and alternative medicine
CANONIC-CLIF: Acute-oN-ChrONic LIver failure in cirrhosis
CLIF: Chronic Liver Failure Consortium
CNI: Calcineurin inhibitor
DILI: Drug-induced liver injury
DDLT: Deceased donor liver transplant
DI-ACLF: Drug-induced ACLF
FMT: Fecal microbiota transplant
GRWR: Graft Recipient Weight Ratio
GM: Gut microbiota
HCC: Hepatocellular carcinoma
HDS: Herbs, drugs and supplements
HBV: Hepatitis B virus
HE: Hepatic encephalopathy
HRS: Hepato renal syndrome
HVPG: Hepatic venous pressure gradient
LoE: Level of evidence
LDLT: Living donor liver transplant
LT: Liver transplant
LVP: Large volume paracentesis
MAFLD: Metabolic-associated fatty liver disease
MARS: Molecular adsorbent recirculating system
MASLD: Metabolic-associated steatotic liver disease
MVP: Modest volume paracentesis
MSC: Mesenchymal stem cell therapy
NSBB: Non-selective beta blocker
NAD: Non-acute decompensation
NSBB: Non-selective beta-blockers
PVT: Portal vein thrombosis
PICD: Paracentesis-induced circulatory dysfunction
PRA: Plasma renin activity
RRT: Renal replacement therapy
SOFA: Sequential organ failure assessment
qSOFA: Quick sequential organ failure assessment
MELD: Model end-stage liver disease
SAH: Severe alcoholic hepatitis
TPPM: Tongji prognostic predictor model
TPE: Therapeutic plasma exchange
