# Supplementary material for: Acute-on-chronic liver failure (ACLF): the ‘Kyoto Consensus’—steps from Asia
Source: Hepatol Int. 2025 Feb 17;19(1):1–69. doi: 10.1007/s12072-024-10773-4 (PMC11846769; doi:10.1007/s12072-024-10773-4)
Supplement: Supplementary file 1 — Supplementary file1 (DOCX 358 KB) [file 12072_2024_10773_MOESM1_ESM.docx]

**
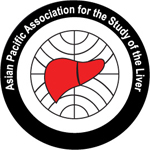
**   **
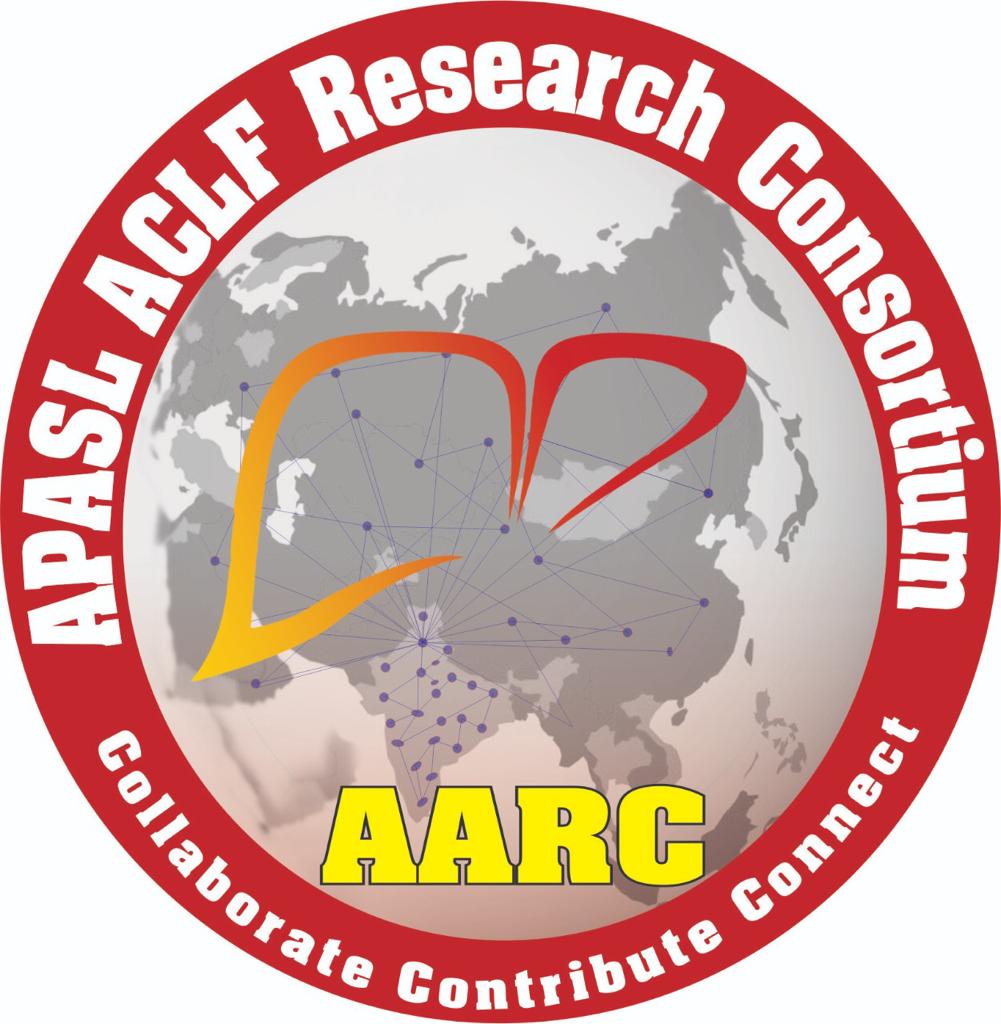
**

**6^th^ APASL- ACLF Consensus Meeting**

**14^TH^ Annual Meeting of APASL- ACLF Research Consortium (A.A.R.C)**

Theme: “**Acute-On-Chronic Liver Failure (ACLF) towards a Global Consensus**” `

**Date -March 29 (Friday), 2024.**

**Time: 8:00 AM To 6:00 PM**

**Venue: - Room 558, 5F, Kyoto International Conference Center, Japan**

**Hybrid Event**

**SCIENTIFIC PROGRAM**

**Theme: “Acute-On-Chronic Liver Failure (ACLF): Towards a Global Consensus”**

| **Section Sl.** | **Section Heading** | **Virtual Meeting Date** | **Virtual Meeting Time** |
| --- | --- | --- | --- |
| **Section-III** | **Infection and/or Sepsis in the Natural History of Chronic Liver Disease** | **07-03-2024 (Thursday)** | **04:30 PM (IST)** |
| **Section-IV & V** | **Organ Failure and its implication in patients with Liver Failure due to an Acute Insult   AND  Definition of ACLF** | **09-03-2024 (Saturday)** | **04:30 PM (IST)** |
| **Section-VI** | **Defining the Acute Insult in ACLF** | **10-03-2024 (Sunday)** | **10:30 AM (IST)** |
| **Section-VII-VIII** | **Defining the underlying chronic liver disease in ACLF  AND  ACLF and Acute Decompensation (AD) are Distinct: Differentiating AD and ACLF** | **12-03-2024 (Tuesday)** | **04:30 PM (IST)** |
| **Section-IX-X** | **Portal and Systemic hemodynamic in ACLF  AND Role of Liver histology in ACLF** | **14-03-2024 (Thursday)** | **04:30 PM (IST)** |
| **Section-XI** | **Treatment of ACLF** | **21-03-2024 (Thursday)** | **04:30 PM (IST)** |
| **XII, XII & XIV** | **Liver Transplant in ACLF   AND Prognostic Models in ACLF   AND Prognostic Models and Global Convergence in ACLF** | **24-03-2024 (Sunday)** | **10:30 AM (IST)** |

**Organized By**

**APASL ACLF Research Consortium (AARC)**

**
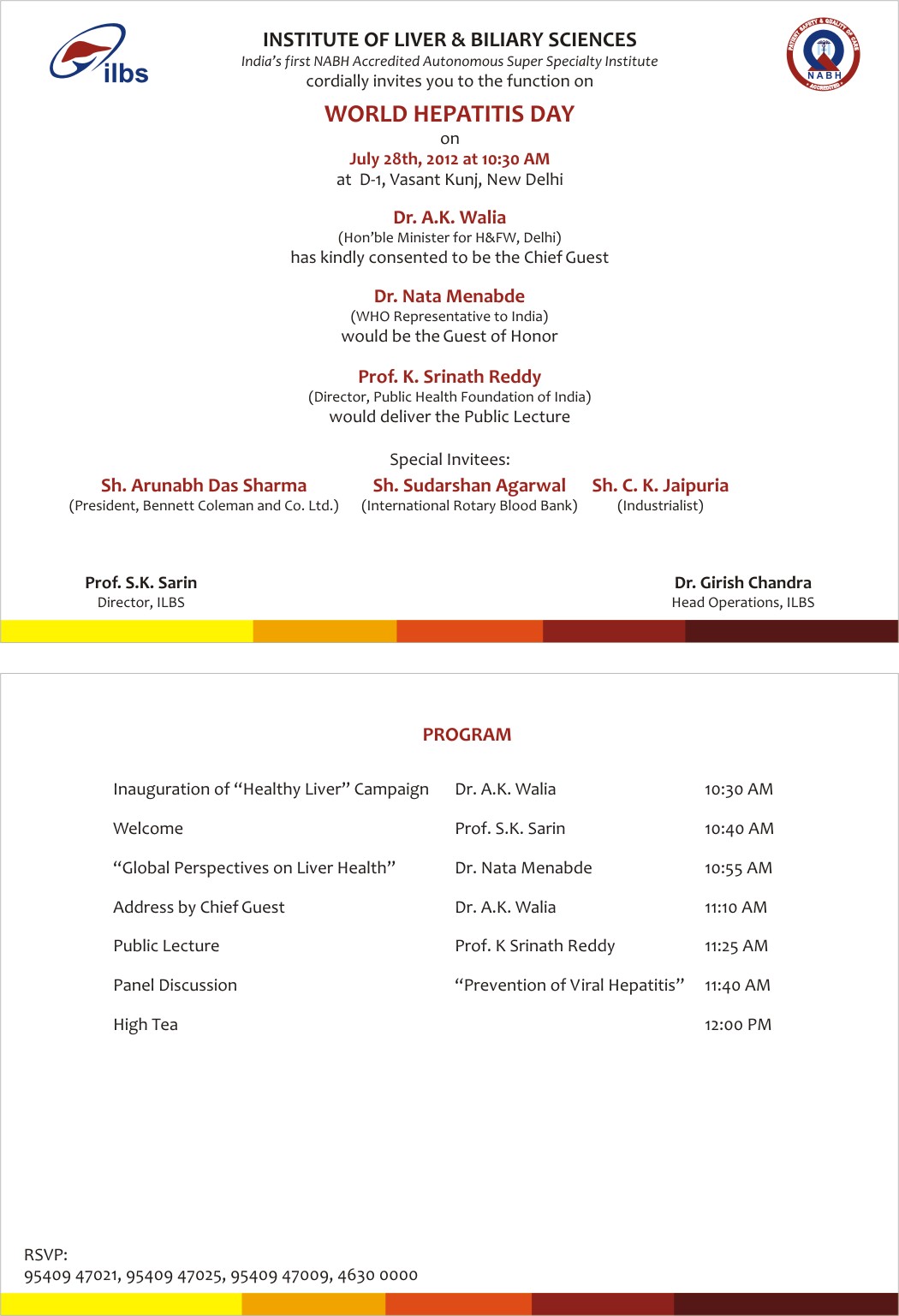
**

**Page-1**

**Agenda**

**Section-I:** **Introduction**.

| 1 | ACLF: towards a Global Consensus | **C.E Eapen** | **Sagnik Biswas** | **Jin Mo Yang** |
| --- | --- | --- | --- | --- |

**Section-II:** **The Concept of Hepatic Reserve and the Liver Failure**.

**Drafting in-Charge: D J Kim, Wasim Jafri, Jia Ji Dong**

| 2a | Natural history of Cirrhosis and Decompensation and causes of death | Gennaro D'Amico | Gupse Adali | Guan-Huei Lee |
| --- | --- | --- | --- | --- |
| 2b | Recompensation-The concept and implication for diagnosis of ACLF | Ashwani K. Singal | Nipun Verma | Hasmik Ghazinian |
| 2c | Further decompensation in a decompensated cirrhosis- defining AD, ACLF, ESLD | Jonel Trebicka | Apichat Kaewdech | Vinod Arora |
| 2d | Reversal of ACLF Syndrome- Timeline, predictor and Outcome | Zaigham Abbas | V Rajan | Soek Siam Tan |
| 2e | Reversal of Cirrhosis- the aetiology, time frame and implication for ACLF | K. Rajender Reddy | Babu Lal Meena | Ruveena Bhavani |
| 2f | Unified Prognostic Model for ACLF and Steps for Prioritization for LT in ACLF | Sumeet Asrani | Sargsyan Violeta | Sakkarin Chirapongsathorn |

*Repertoire- Omesh Goyal and Omkar*

**Section-III: Infection and/or Sepsis in the Natural History of Chronic Liver Disease**

**Drafting in-Charge: Ajay Duseja, A Kadir Dokmeci, Yoon Jun Kim**

| 3a | Infection leading to Liver Failure in Cirrhosis | Qin Ning | Rahul Kumar | Shantan Venishetty |
| --- | --- | --- | --- | --- |
| 3b | Infection leading to other OF followed by Liver Failure | Debbie Shawcross | Kemal Fariz Kalista | Pravin Rathi |
| 3c | Infection leading to other organ failure and death without liver failure. | Jasmohan Bajaj | Kessarin Thanapirom | Ameet Mandot |
| 3d | Lab and clinical Diagnosis of infection in Cirrhosis with or without decompensation. | Do Seon Song | Mark Dhinesh Muthiah | Ajay Kumar Patwa |
| 3e | Hepato tropic versus non-hepato tropic infections | Diana Alcantara-Payawal | Vinay Kumar B R | M Golam Azam |

*Repertoire-Sanjeev Sachdeva, Saurav* **Page-2**

**Section-IV: Organ Failure and its implication in patients with Liver Failure due to an Acute Insult**

**Drafting in-Charge: Saeed Hamid, George K Lau, Anand Kulkarni**

| 4a | Defining the Organ Dysfunction and Organ Failure | V M Dayal | Anand Kulkarni | Chien Hao Huang |
| --- | --- | --- | --- | --- |
| 4b | Hepatic vs. Extrahepatic OD/OF in a Decompensated cirrhosis | Jennifer C. Lai | Lubna Kamani | Sachin Palnitkar |
| 4c | Renal Dysfunction or Failure- the course and implication in cirrhosis with predominant liver failure | Rakhi Maiwall | Shahinul Alam | Sudhir Maharshi |
| 4d | Renal Dysfunction or Failure- the course and implication in cirrhosis with acute decompensation with other extra-hepatic complications/OF | Paolo Angeli | H. Janaka De Silva | Shiran Shetty |
| 4e | Circulatory and Respiratory failure are terminal events in ESLD rather than type of Organ Failure | Constantine J. Karvellas | Ananta Shrestha | Narendra Choudhary |

*Repertoire-Girish Kumar Pati, Ayush Jain*

**Section-V: Definition of ACLF**

**Drafting in-Charge: Sunil Dadhich, Gamal Shiha & Surender Singh**

| 5a | APASL Definition of ACLF | Omesh Goyal | Bui Huu Hoang | Punit Puri |
| --- | --- | --- | --- | --- |
| 5b | Other Existing definition for ACLF | Ajay Mishra | Vandana Midha | PN Rao and Sunil Dadhich |
| 5c | Similarities and differences in existing definitions. | Soe Thiha Maung | Karan Kumar | Hai Li |
| 5d | Gaps to be bridged for a global definition | Amrish Sahney | Dharmesh Kapoor | Joy Varghese |

*Repertoire- Girish Kumar Pati, Ayush Jain*

**Page-3**

**Section-VI: Defining the Acute Insult in ACLF**

**Drafting in-Charge: Akash Shukla, Krishnadas Devdasa & Eugene Wong Yu Jun**

| 6a | Alcoholic Hepatitis in ACLF and development of jaundice in decompensated Cirrhosis- Are they same? | Gyongyi Szabo | Sunil Taneja | Fazal Karim |
| --- | --- | --- | --- | --- |
| 6b | Hepatitis B Reactivation | Yu Shi | Jin Jun Chen | Chun Yen Lin |
| 6c | Acute Viral Hepatitis -Hepatotropic and non-hepatotropic CD Virus | Dibya L Praharaj | Jidong Jia | Teerha Piratvisuth |
| 6d | Drug Induced Liver Injury (Drugs, CAM & HDS) - with and without cirrhosis | Dhiraj Agrawal | Harshad Devarbhavi | Madunil A Niriella |
| 6e | Autoimmune Liver Disease – distinction in resentation as ACLF and ALF | Manasa Alla | Atsushi Tanaka | Jose D Sollano |
| 6f | Vascular diseases (PVT, HVOTO) | Pathik Parikh | Akash Shukla | Simone Strasser |
| 6g | Infection and Sepsis | Rosmawati Binti Mohamed and Jaideep Behari | Arun Valsan | Amna Subhan Butt |
| 6h | Variceal Bleed, post- TACE or post-hepatectomy liver failure | Aleksander Krag | Ashish Goel | Amar Mukund |
| 6i | Timeline of acute injury and Liver failure with or without other OF- implication for definition (4 weeks versus 12 weeks) | Nadim Mahmud | Suprabhat Giri | Sanjiv Saigal |

*Repertoire- Phool Chand*

**Section-VII: Defining the underlying chronic liver disease in ACLF**

**Drafting in-Charge: Rino Gani, C Rinaldi Lesmana, Mohd Eslam**

| 7a | CLD with or without Cirrhosis-implications in therapy and response | Tawesak Tanwandee | Tarana Gupta | Virendra Singh |
| --- | --- | --- | --- | --- |
| 7b | ‘Index’ presentation or ‘ACLF Again’ | Ashish Kumar | Manoj Sahu | Ashok Choudhury |
| 7c | Impact of Comorbidities and Obesity | Rohit Loomba | Jacob George | Hong Ling Yu and Anand Kulkarni |
| 7d | Changing trends for the aetiology of the acute insult and chronic injury | Jun Li | Tao Chen | Piyush Ranjan |

*Repertoire- Suguna Sree*

**Page-4**

**Section-VIII: ACLF and Acute Decompensation (AD) are Distinct: Differentiating AD and ACLF**

**Drafting in-Charge:** Goeff Mcgauhan, James Fung, Mamun Al Mehtab, Tawesak Tanwandee

| 8a | Natural history and outcome ACLF | James Fung | Manoj Kumar Sharma | A. Kadir Dokmeci |
| --- | --- | --- | --- | --- |
| 8b | Natural history and outcome of Acute Decompensation | Abhijeet Choudhary | Kaushal Madan | Hemamala Ilango |
| 8c | Acute Decompensation –differentiating from ACLF for clarity in definition | Khin Maung Win | Sombat Tressurepark | Irsan Hasan |
| 8d | Hepatic encephalopathy -Prevention, therapy and implication in natural history of ACLF | Necati Ormeci | Sanjeev Sachdeva | A C Anand |

*Repertoire- Suguna Sree*

**Section-IX: Portal and Systemic hemodynamic in ACLF**

**Drafting in-Charge:** Ankur Jindal, Harshvardhan Tevethia, Zaigham Abbas

| 9a | Acute Portal Hypertension in ACLF i.e. the Variceal bleed & Ascites | Xiaolong Qi | Akash Roy | Anil Arora |
| --- | --- | --- | --- | --- |
| 9b | Variceal progression in ACLF and role of preemptive BB therapy | Chitranshu Vasishtha | Mamun Al Mehtab | Ankur Jindal |
| 9c | PICD-incidence, presentation, diagnosis and management | Vinod Arora | Hari Kumar Nair | Saurabh Mukewar |
| 9d | Systemic, hepatic and pulmonary hemodynamics in ACLF | Jin Hua Hu | Madhumita P | Shobna Bhatia |

*Repertoire- Charles Panackel, Sudhir Verma*

**Section-X: Role of Liver histology in ACLF**

**Drafting in-Charge:** Mukul Vij, Amit Goel, Y K Chawla

| 10a | Diagnostic Implication | Archana Rastogi | K C Sudhamshu |
| --- | --- | --- | --- |
| 10b | Prognostic Implication | Chhagan Bihari | Chen Yu |
| 10c | Safety, feasibility and cost | Puja Sakhuja | Venera Rakhmetova |

*Repertoire- Charles Panackel,*

**Page-5**

**Section-XI: Treatment of ACLF**

**Drafting in-Charge:** Sombat T , A Tanaka , Shasthry S M, H C Lin

| 11a | Antiviral strategies in ACLF HBV Reactivation (2019) | Dr. Mohamed Elbasiony | M F Yuen | Cesar Yaghi |
| --- | --- | --- | --- | --- |
| 11b | Steroid in Alcoholic ACLF | Juan Pablo Arab | Mithun Sharma | Oidov Baatarkhuu  and  Subrat Acharya |
| 11c | Steroid in DILI and AIH -ACLF | Mithra Prasad | V G Mohan Prasad | Shalimar |
| 11d | Emerging therapies in ACLF-Anti-inflammatory, immune-modulators and others | Shyam Kotilil | Abraham Koshy | Ajay Kumar GB Pant |
| 11e | Fecal Microbiota Transplant | Ki Tae Suk | C Abby Phillips | Apurva Pande  And  Guru Prasad  Dhakal |
| 11f | Regenerative and cell-based Therapy | Ajit Sood | Anupam Kumar | Osamu Yokosuka |
| 11g | Liver dialysis, Plasmapheresis and other artificial liver support system | Santhosh E Kumar | Kaiser Raja | Meenu Bajpai |

*Repertoire- Mukesh Sharma Paudel, Amanjot Kaur*

**Section-XII: Liver Transplant in ACLF**

**Drafting in-Charge:** S G Lim, , Rohit Mehtani, Dong-Sik Kim, Mohd. Rela, Albert Chan

| 12a | Prediction of Liver Transplant-the need, Transplant window and optimization | Paul J Thuluvath | Chang Wook Kim and  Mettu Srinivas Reddy | Viniyendra Pamecha |
| --- | --- | --- | --- | --- |
| 12b | Unsuitability, contra-indications and futility of LT in ACLF | Kamal Kajal | Neeraj Saraf | Audrey Coilly |
| 12c | Prioritization for LT in live and cadaveric transplant | Francoiz Durand | Navin Kumar Marannan | Md. Rela |
| 12d | Right time for transplant in alcoholics with ACLF | D S Kim | Sanjiv Saigal | Sonal Asthana |
| 12e | Innovations in technique and management for improved transplant outcomes in ACLF | Ravi Mohanka | Prashant Bhangui | Subhash Gupta and Shweta A Singh |
| 12f | Immune-suppression and ACLF -are they same or different | Dinesh Jothimani | A S Soin | Satendra Pal Singh |
| 12g | Nutritional assessment and interventions | Manav Wadhawan | Jaya Benjamin | Anoop Saraya |

*Repertoire- Rahul Pathakd, Ananthu*

**Page-6**

**Section-XIII: Prognostic Models in ACLF**

**Drafting in-Charge:** Z Duan, Tao Chen, Manya Prasad

| 13a | ACLF patient in ICU- Prognostic score, AI MODELS | J Fernadez | Vivek Saraswat | Sargsyan Violeta |
| --- | --- | --- | --- | --- |
| 13b | Current Prognostic Models - Strength and Weaknesses | R K Dhiman | Samagra Agarwal | Amna Subhan Butt |

*Repertoire- Rahul Pathakd, Ananthu*

**Section-XIV: Prognostic Models and Global Convergence in ACLF**

**Drafting in-Charge:** Vincet Wong, Van Huy Vo, Qin Ning

| 14a | ACLF in East and West are different patients than a different definition: Steps to convergence | Richard Moreau | Chetan R Kalal | Chandan K N |
| --- | --- | --- | --- | --- |
| 14b | Convergence 2024 for an ACLF unified Definition | S.K. Sarin | Vincent Wong | Van Huy Vo |

*Repertoire- Rahul Pathakd, Ananthu*

**Title: Acute-on-chronic liver failure: Consensus recommendations of the Asian Pacific Association for the Study of the Liver (APASL): An update and Steps Towards a Global Consensus**

**Keywords:**Liver failure, ACLF, AARC, Chronic liver disease, Global Consensus.

**Page-7**

**Outline of the Consensus meeting**

**Phase-1: Preparation of Draft**

**Formation of section with Team of 2 to 3 experts as Draft Incharge**

Your role as an expert will involve presenting a Consensus Draft, comprising 1-2 paragraphs (approximately 300-500 words) with 5-6 references and 4-6 consensus statements following the GRADE system. The draft will be a focal point for discussion and approval by the panelists, as well as the speaker and moderator in a subsequent Hybrid presentation.

Data from AARC database is there, if you think of any analysis to be incorporated, our statistical team and AARC nodal Centre is happy to help.

**Phase-2: Virtual meeting for approval of the Draft**

Initial Section wise meeting of experts, Draft In charge as well as Invited faculties will be done virtually after the draft is received. This will make the first consensus draft.  The draft in-charge for the section will lead the meeting and the final section draft will be shared to us.

**Phase-3: Hybrid Meeting during APASL 2024 at Kyoto**

The approved draft and selected topics which are needed to be discussed as per theme will be presented at the hybrid consensus meeting of AARC in Kyoto during APASL 2024 ON 29^th^ March 2024.

**Guideline for the Consensus Draft**

·        Draft within 500 words.

·        4 or 5 consensus statements (as per GRADE, attached below).

·        Maximum of 5-6 references

·        Any conceptualized figure/table is most welcome.

**Page-8**

 Table 1 Level of Evidence and Grade of Recommendations (Adapted from Grading of Recommendations, Assessment, Development and Evaluations [GRADE] System with Minor Modifications^a^).

|  | **Level of evidence^b^** | **Confidence in the evidence** |
| --- | --- | --- |
| High | Information obtained from meta-analyses or systematic reviews, or from numerous randomized trials that have high quality data | It is improbable that additional research will significantly alter our level of confidence in the ssessment of potential benefits and risks |
| Moderate | Information obtained from either a singular randomized controlled trial (RCT) or various non-randomized studies | Additional research, if conducted, may potentially alter our estimation of the benefit and risk and have an impact on our level of confidence in the estimate. |
| Low | Studies of limited sample size, observational studies conducted retrospectively, and registries. | There is a degree of uncertainty associated with any estimate of the effect. |
|  | **Recommendations – Grade^c^** | **Wording associated with the grade of recommendation** |
| Strong | The strength of the recommendation was influenced by several factors, such as the quality of the evidence, the presumed outcomes that are important for the patient, and the cost implications | “must”, “should”, or “we recommend |
| Weak | The recommendation may be made with less certainty and may result in higher costs or resource consumption due to variability in preferences and values, or increased uncertainty | “can”, “may”, or “we suggest |

a-To make the GRADE system more objective, the type of studies from which the evidences are derived have been mentioned in the Level of Evidence

b-Level was graded down if there was a poor quality, strong bias or inconsistency between studies; level was graded up if there was a large effect size.

c-Recommendations reached by consensus of the members and included the quality of evidence, presumed patient-important outcomes and costs.

1.      Atkins D, Best D, Briss PA, et al. Grading quality of evidence and strength of recommendations. BMJ. 2004; 328:1490. <https://doi.org/10.1136/bmj.328.7454.1490>.

2.      Guyatt GH, Oxman AD, Vist GE, et al. GRADE: an emerging consensus on rating quality of evidence and strength of recommendations. BMJ. 2008; 336:924–926. <https://doi.org/10.1136/bmj.39489.470347.AD>.

**Kindly Email in-**[**aarc@aclf.in**](mailto:aarc@aclf.in)

**In case of any confusion, can reach us by email or WhatsApp Call/message**

Radhika- AARC Research Coordinator +91 9540947087

Dr Ashok Choudhury- Coordinator AARC. [Email-doctor.ashokchoudhury@gmail.com](mailto:Email-doctor.ashokchoudhury@gmail.com)

Dr Anand Kulkarni- Editorial Team. Email-[anandvk90@gmail.com](mailto:anandvk90@gmail.com)

Dr Vinod Arora-PI AARC. Email-[vinod_ucms@yahoo.com](mailto:vinod_ucms@yahoo.com)

**Page-9**
