# Supplementary material for: Acute-on-chronic liver failure (ACLF): the ‘Kyoto Consensus’—steps from Asia
Source: Hepatol Int. 2025 Feb 17;19(1):1–69. doi: 10.1007/s12072-024-10773-4 (PMC11846769; doi:10.1007/s12072-024-10773-4)
Supplement: Supplementary file 2 — Supplementary file2 (DOC 88 KB) [file 12072_2024_10773_MOESM2_ESM.doc]

| Sl no | Site/Centre | Country | Data |
| --- | --- | --- | --- |
|  | 302 Hospital Beijing | China | 105 |
|  | Aga Khan University Hospital, Karachi, Pakistan | Pakistan | 96 |
|  | AIG, Hyderabad | India | 387 |
|  | AIIMS, New Delhi | India | 53 |
|  | Alka Hospital | Nepal | 28 |
|  | Amrita Hospital, Kochi | India | 210 |
|  | Ankara University, Turkey | Turkey | 11 |
|  | Apollo Hospital Kolkata | India | 28 |
|  | Apollo Hospital, Gandhi Nagar | India | 10 |
|  | Aster Medcity | India | 10 |
|  | Bangabandhu Sheikh Mujib Medical university | Bangladesh | 410 |
|  | SMNC | India | 133 |
|  | Cardinal Santos Medical Center | Philippines | 19 |
|  | Chiba University | Japan | 2 |
|  | Chulalongkorn, Bangkok | Thailand | 108 |
|  | Chuncheon Sacred Heart Hospital | Korea | 73 |
|  | Cipto Gen Hospital, Indonesia | Indonesia | 118 |
|  | CMC Vellore | India | 210 |
|  | DMC & Hospital, Ludhiana | India | 235 |
|  | Dr. B R Ambedkar Institute, Mohali | India | 10 |
|  | Dr. Rela Institute, Chennai | India | 215 |
|  | Egyptian Liver Research Institute And Hospital (ELRIAH), Egypt | Egypt | 43 |
|  | FACULTY OF MEDICINE, KELANIYA | Indonesia | 10 |
|  | G.B. Pant Hospital | India | 91 |
|  | Gleneagles Global, Chennai | India | 170 |
|  | GLOBAL HOSPITAL,MUMBAI | India | 42 |
|  | Global Hospitals, Hyderabad | India | 22 |
|  | Hallym University Medical Center Chuncheon Sacred Heart Hospital | Korea | 398 |
|  | Hebei Medical University | China | 17 |
|  | Humanity & Health Medical Center | Hongkong | 14 |
|  | IGIMS, Patna | India | 94 |
|  | IMS & SUM Hospital, Bhubaneswar | India | 247 |
|  | Institute of liver and biliary sciences | India | 3989 |
|  | Kem Hospital Mumbai | India | 255 |
|  | KGMC, Lucknow | India | 24 |
|  | Lakeshore Hospital. Kochi | India | 48 |
|  | Liaquat National Hospital-Pakistan | Pakistan | 8 |
|  | LTMMC, Mumbai | India | 81 |
|  | Mansoura University, Egypt | Egypt | 10 |
|  | Max Super Speciallity Hospital, Saket | India | 10 |
|  | Medanta Hospital, Gurgaon | India | 421 |
|  | Medical School of Chinese PLA, Beijing | China | 26 |
|  | Medistra Hospital | Indonesia | 86 |
|  | Midas Hospital, Nagpur | India | 89 |
|  | Nanavati Max Super speciality Hospital,Mumbai | India | 8 |
|  | Nork Inf. Hospital, Armenia | Armenia | 138 |
|  | National University Health System | Singapore | 89 |
|  | Post Graduate Institute of Medical and Science Research, Chandigarh | India | 290 |
|  | SCB Medical College | India | 24 |
|  | Selang Hospital, Kepong | Malaysia | 221 |
|  | SGPGI, Lucknow | India | 10 |
|  | Sir Ganga Ram Hospital, | India | 165 |
|  | Sir Salimullah Medical College Hospital | Bangladesh | 133 |
|  | SIUT, Pakistan | Pakistan | 16 |
|  | SMS Jaipur | India | 46 |
|  | St.John’s Medical College and Hosp, Bangalore | India | 221 |
|  | SUM Ultimate Medicare, Bhubandeshwar | India | 47 |
|  | Tianjin Institute of Hepatobiliary Disease, Tianjin | China | 35 |
|  | TN Medical College and BYL Nair Hospital, Mumbai | India | 18 |
|  | Tongji Hospital, Wuhan | China | 262 |
|  | You'an Hospital, Beijing | China | 52 |
|  | University of Santo Tomas Hospital, Manila, Philippines | Philippines | 37 |
|  | University of Malaya Medical Centre, Malaysia | Malaysia | 88 |
|  | VGM Gastro Centre, Coimbatore | India | 131 |
|  | Ziauddin Hospital Karachi | Pakistan | 42 |
|  | Zydus Hospital Ahmedabad | India | 7 |
|  | Violeta Medical Center | Armenia | 136 |
|  | Pandit Bhagwat Dayal Sharma Post Graduate Institute of Medical Sciences, Rohtak | India | 10 |
|  | Kalinga Institute of Medical Sciences (KIMS), Bhubaneshwar | India | 2 |
|  | Punjab Institute of Liver and Biliary Sciences, Mohali | India | 7 |
|  | Aster MIMS, Kannur, Kerala | India | 62 |
|  | Kasturba Medical College, Manipal | India | 5 |
|  | Istanbul Umraniye Training and Research Hospital, Turkey | Turkey | 13 |
|  | Government Medical College, Trivandrum | India | 13 |
|  | Total |  | 10,994 |
